# Improvement of Clinical Practice Guideline Appraisal by Human Experts and AI Agents by Using Structured Guidance: Systematic Review, Meta-Analysis, and Validation Study

**DOI:** 10.2196/96002

**Published:** 2026-08-26

**Authors:** Xingrun Mao, Junhao Wang, Zezhang Wang, Yuwei Zhang, Shiyu Qiu, Jingyu Ye, Shiyan He, Chongyang Wang, Yong Xia, Chengqi He, Ke Li, Siyi Zhu

**Affiliations:** 1 Rehabilitation Medicine Center and Institute of Rehabilitation Medicine West China Hospital, Sichuan University Chengdu, Sichuan China; 2 Key Laboratory of Rehabilitation Medicine in Sichuan Province, West China Hospital, Sichuan University Chengdu, Sichuan China; 3 Center of Statistical Research, School of Statistics and Data Science, Southwestern University of Finance and Economics Chengdu, Sichuan China; 4 Joint Lab of Data Science and Business Intelligence, School of Statistics and Data Science, Southwestern University of Finance and Economics Chengdu, Sichuan China; 5 West China School of Medicine, West China Hospital, Sichuan University Chengdu, Sichuan China

**Keywords:** AGREE II, large language models, practice guideline, rehabilitation, RIGHT

## Abstract

**Background:**

Rehabilitation clinical practice guidelines (CPGs) have increased rapidly, but inconsistent methodological quality limits their implementation. Although Appraisal of Guidelines for Research and Evaluation II (AGREE II) and Reporting Items for Practice Guidelines in Health Care (RIGHT) provide standardized appraisal frameworks, their application is time-consuming. Large language model (LLM)–based AI agents may offer a scalable alternative with uncertain reliability.

**Objective:**

We evaluated rehabilitation CPGs’ methodological and reporting quality and determined whether structured guidance improves human expert–AI agent agreement.

**Methods:**

We systematically reviewed English- and Chinese-language rehabilitation CPGs from Embase, Scopus, PubMed, China National Knowledge Infrastructure, Wanfang Data, National Institute for Health and Care Excellence, Scottish Intercollegiate Guidelines Network, and Guidelines International Network up to June 2026. Methodological and reporting quality were assessed using AGREE II and the RIGHT checklist. Factors associated with guideline quality were examined using regression and subgroup analyses. Two AI agents were compared with human consensus with and without a structured guideline appraisal workbook, followed by external validation using 6 anterior cruciate ligament reconstruction CPGs.

**Results:**

We included 227 CPGs (163 English-language, 64 Chinese-language). After introducing a structured guideline appraisal workbook, agreement among human experts improved markedly—mean intraclass correlation coefficients (ICCs) increased from –0.09 to 0.66 to 0.84-0.92 across AGREE II domains. Overall guideline quality remained low, with 35.9% (SD 18,8%) applicability and 52% (SD 17.2%) stakeholder involvement. English-language guidelines outperformed Chinese-language guidelines in scope and purpose (mean 74.64, SD 15.4 vs mean 68.88, SD 13.9; *P*=.004) and applicability (mean 39.14, SD 18.6 vs mean 27.54, SD 16.7; *P*<.001). Backward-elimination logistic regression revealed external review as an associated process characteristic (odds ratio 20.39, 95% CI 4.66-89.27; *P*<.001). RIGHT assessments showed consistent reliability (ICC=0.80-0.88). Reporting was highest for basic information (70.5%) and lowest for funding, declaration, and management of interests (44.1%). Meta-analysis of RIGHT reporting rates showed lower reporting among Chinese-language than English-language guidelines (risk difference [RD] –0.07, 95% CI –0.13 to –0.02, 95% prediction interval [PI] –0.39 to 0.24) and among guidelines published before vs after RIGHT release (RD –0.19, 95% CI –0.26 to –0.13, 95% PI –0.56 to 0.17). Without additional guidance, agent-human agreement was moderate (ICC=0.608-0.629). The workbook improved agreement for both models, with DeepSeek-R1’s increasing from 0.613 to 0.709 and o1-mini’s from 0.629 to 0.687. In validation beyond rehabilitation, DeepSeek-R1 maintained stable agreement (ICC=0.711) and completed appraisals in 5.44 minutes compared to 11.18 minutes for humans.

**Conclusions:**

Rehabilitation CPGs, particularly Chinese-language CPGs, continue showing deficiencies in applicability and stakeholder involvement. LLM-based appraisal without structured guidance provides insufficient agreement. Structured guidance improved agent-human agreement, supporting AI-assisted guideline appraisal under human oversight. Although further validation across additional clinical specialties is needed, AI agents can serve as efficient assistants in guideline appraisal instead of replacing humans. Future synthesis requires human-AI integration guided by structured, expert-defined principles.

**Trial Registration:**

PROSPERO CRD420251270676; https://www.crd.york.ac.uk/PROSPERO/view/CRD420251270676

## Introduction

### Rationale

The World Health Organization (WHO) estimates that over 1 billion people worldwide need rehabilitation services to improve quality of life and functioning [1], with demand rising due to population aging, improved childhood survival, and increasing chronic diseases. Most of those in need live in low-resource settings with limited access [2]. In response, WHO launched Rehabilitation 2030: A Call for Action in 2017, emphasizing universal access to high-quality rehabilitation as a core health service alongside promotive, preventive, curative, and palliative care [3-6].

Evidence-based practice now underpins modern rehabilitation; yet, despite 3 decades of research, ineffective practices remain common, contributing to suboptimal outcomes [7]. Clinicians and researchers also face practical barriers—including limited time, resources, and methodological expertise—that restrict the translation of research into routine care [8]. Clinical practice guidelines (CPGs) address these challenges by promoting standardized, evidence-informed care tailored to specific conditions. Since CPGs are typically developed through systematic reviews and structured consensus processes by expert panels [9,10], their value depends on methodological quality, internal validity, and relevance to end users. When evidence synthesis lacks rigor, recommendations may become biased, leading to overestimation or underestimation of treatment effects [11,12].

Developing guidelines requires substantial resources, prompting the creation of multiple appraisal instruments. Among 40 tools identified by Siering et al [13], the Appraisal of Guidelines for Research and Evaluation II (AGREE II) has gained the widest acceptance and is regarded as the most suitable for evaluating guideline quality [13,14]. First introduced in 2003 and revised in 2011, AGREE II comprises 23 items across 6 domains plus 2 overall assessment items [15]. AGREE-based syntheses have consistently reported low guideline quality. Grilli et al [16] found 18% to 38% of guidelines not recommended for use, largely due to poor applicability. These shortcomings are particularly relevant to rehabilitation. Across 544 guidelines in 40 reviews, only 36% were recommended without modification, with the largest deficiencies in applicability and rigor of development, the strongest predictor of overall quality [17,18].

Methodological quality alone does not ensure trustworthiness. Clear and transparent reporting is also necessary for dissemination and implementation [19]. To address reporting deficiencies, the Reporting Items for Practice Guidelines in Health Care (RIGHT) Working Group introduced the RIGHT checklist in 2017 [20]. This checklist includes 35 items across 7 domains and has since become a widely used reporting standard. Its “Explanation and Elaboration Statement” provides detailed guidance and examples, complementing AGREE II by adding item-level clarification [21]. An overview assessing 16 rehabilitation guidelines in English and Chinese using the RIGHT checklist reported an average reporting rate of 44.8%. Reporting was highest for basic information (57.3%) and lowest for evidence and other information (31.3%). Reporting differences were associated with country of origin, guideline version, and initiating organization [22].

However, previous reviews have mostly relied on a single appraisal instrument—evaluating methodological quality with AGREE II alone [17,18] or reporting quality with RIGHT alone [22]. A combined assessment of methodological and reporting quality across rehabilitation guidelines is still lacking. Existing reviews have also been largely descriptive, with limited analysis of factors associated with variation in guideline quality or potential targets for improvement. One 2020 report identified 84 rehabilitation guidelines and predicted continued growth in subsequent years, although the total number remained lower than in many other clinical specialties [23]. A clearer understanding of current guideline quality is needed to inform both clinical implementation and future guideline development. To our knowledge, no study has comprehensively evaluated rehabilitation CPGs published in English and Chinese using both AGREE II and RIGHT while also examining determinants of quality.

Guideline appraisal itself presents additional challenges: structured guidance for AGREE II and RIGHT is limited, manual appraisal is labor-intensive and prone to oversight, and language barriers complicate cross-regional assessment [24]. Advances in large language models (LLMs) offer new possibilities for text-intensive tasks like guideline appraisal, enabling efficient processing and structured information extraction [25]. At the same time, limitations persist in complex semantic judgments—a pilot study using LLMs for medical imaging guidelines reported 77% average accuracy, with poor performance on interpretive tasks [24], while another study found GPT-4o consistently generated higher scores than human raters when applying AGREE II and RIGHT [26]. These discrepancies highlight a key uncertainty: whether structured guidance can improve the reliability of guideline appraisal for both human experts and LLM-based AI agents.

### Objectives

To address these gaps, we conducted this study with two objectives: (1) to systematically review rehabilitation CPGs published in English and Chinese, evaluate their methodological and reporting quality using AGREE II and RIGHT with structured guidance, and identify factors associated with guideline quality; and (2) to determine whether the same structured guidance used by human experts could improve agreement between LLM-based AI agents and human expert appraisal.

## Methods

### Protocol and Registration

This systematic review was conducted in accordance with the PRISMA (Preferred Reporting Items for Systematic Reviews and Meta-Analyses) guidelines. The complete PRISMA 2020 expanded checklist, the PRISMA-S (Preferred Reporting Items for Systematic Reviews and Meta-Analyses Literature Search Extension [27]) and the PRISMA 2020 extension for Abstracts are provided in Multimedia Appendices 1-3. AI agent–assisted appraisal followed the Transparent Reporting of a Multivariable Model for Individual Prognosis or Diagnosis-LLM (TRIPOD-LLM) framework (Multimedia Appendix 4) [28]. The protocol was registered in PROSPERO (International Prospective Register of Systematic Reviews; registration number CRD420251270676). The conduct of this review was consistent with the registered protocol, with no deviations.

### Eligibility Criteria

We included documents that met all of the following criteria: (1) constituted a complete CPG; (2) addressed rehabilitation medicine, defined as interventions delivered or prescribed by rehabilitation professionals to optimize functioning, promote independence, and manage health conditions [29]; (3) were published in English or Chinese; and (4) publication year was unrestricted.

We excluded consensus statements without systematic evidence support, systematic reviews, abstracts, commentaries, interpretations, duplicate publications, and translated versions of existing guidelines.

### Information Sources

We carried out a comprehensive search to identify rehabilitation CPGs without publication-date restrictions. The initial search took place in February 2024, was updated in May 2025 and June 2026 by rerunning it. We searched Embase, Scopus, PubMed, China National Knowledge Infrastructure, and Wanfang Data. To ensure coverage of established guidance, we also screened major guideline repositories, including the National Institute for Health and Care Excellence (NICE), the Scottish Intercollegiate Guidelines Network, and the Guidelines International Network (GIN). To ensure a comprehensive search, we screened gray literature, unpublished materials, and reference lists of eligible CPGs, and contacted study authors, sponsors, and experts to clarify methods or obtain missing data.

### Search Strategy

Full search strategies and terms, including controlled vocabulary terms (eg, MeSH and Emtree) such as “Rehabilitation” with relevant free-text terms (eg, “rehabilitation,” “Habilitation,” “physical therapy,” “exercise,” “assistive technology,” “electric stimulation therapy,” “occupational therapy,” and “speech therapy”) and “guidelines” with relevant free-text terms (eg, “practice guideline,” “clinical practice guideline,” “recommendation,” “guidance,” and “guide*”) are reported in the Multimedia Appendix 5. The search strategy was developed in consultation with an experienced librarian. No published search filters were used. No previously published search strategies were adopted or adapted.

### Selection Process

Search results were imported into EndNote (version 20; Clarivate) and deduplicated. Two reviewers independently screened titles and abstracts to identify potentially eligible guidelines. Full texts were retrieved and assessed against the inclusion and exclusion criteria. Disagreements were resolved through discussion, with a third reviewer consulted if consensus was not reached.

### Data Collection Process

Data collection followed a standardized form. One reviewer extracted guideline characteristics, and a second reviewer checked all entries for accuracy. Discrepancies were resolved by rereviewing the full text. We collected data narratively in 3 steps. First, extracted characteristics were compiled. Second, 2 reviewers confirmed the classification of these characteristics, resolving disagreements with a third reviewer. Third, characteristics were grouped and summarized according to the prespecified analytical framework.

### Data Items

Extracted variables included title, authorship, country or region of origin, publication journal and year, developing institution, funding, target condition, guideline type, panel size, evidence grading system, development methods, recommendation formulation methods, number of recommendations, reference to quality tools, update policy, external review, version, and language.

### Quality and Reporting Appraisal (by Human)

#### AGREE II Instrument

We assessed methodological quality using the AGREE II instrument, which includes 23 items across 6 domains: scope and purpose, stakeholder involvement, rigor of development, clarity of presentation, applicability, and editorial independence. Four appraisers independently appraised each guideline on a 7-point Likert scale from 1 (strongly disagree) to 7 (strongly agree). Domain scores were calculated by summing item scores and scaling them as percentages of the maximum possible score (0%-100%).

**Figure 1:**
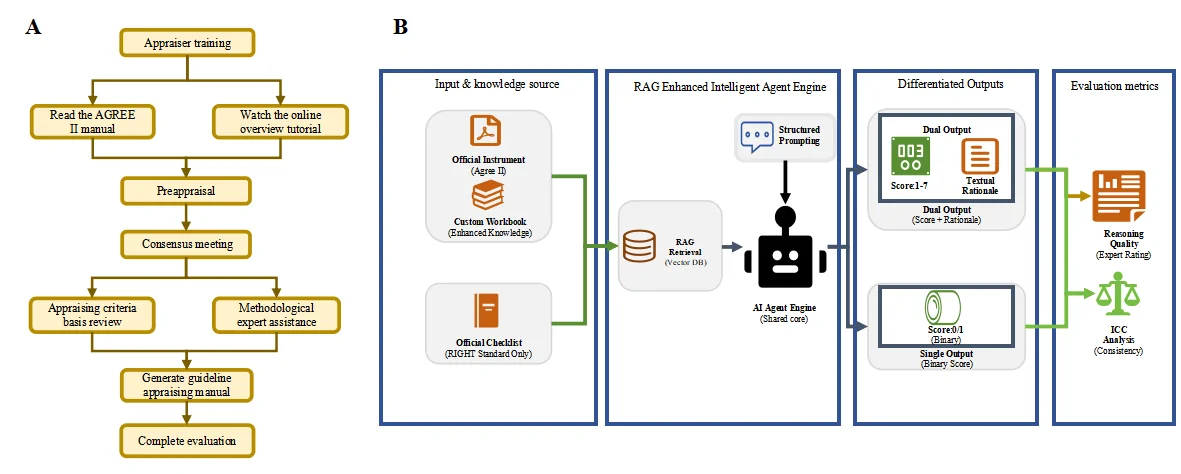
Workflow of clinical practice guidelines appraisal. (A) Human consensus process and the generation of a structured guideline appraisal workbook. (B) The large language model–based agent-assisted appraisal experiment. AGREE II: Appraisal of Guidelines for Research and Evaluation II; ICC: intraclass correlation coefficient; RAG: retrieval-augmented generation; RIGHT: Reporting Items for Practice Guidelines in Health Care.

Before formal appraisal, all appraisers completed calibration training using the AGREE II manual and tutorial. A pilot assessment was followed by consensus meetings with methodological experts to reduce interpretive variation. The appraisal workflow is shown in Figure 1A. The formal process included 3 rounds. In the first round, appraisers scored guidelines independently. After the second round, we identified substantial interrater variation. To improve consistency, particularly given that internal consistency may be affected in smaller samples [30], we developed a structured guideline appraisal workbook (hereafter, workbook). Based on methods described by Brouwers et al [31], the workbook translated the 7-point scale into 3 performance levels (high, low, and very low) with item-specific examples. The workbook was finalized through expert consensus (Document 1 in Multimedia Appendix 5). In the third round, appraisers reapplied the AGREE II tool using the workbook.

Following established criteria [30], we primarily classified a guideline as “high quality” if it achieved scores of at least 60% in 5 or more domains, excluding applicability. All others were classified as “low quality.” In addition to overall classification, we defined domain-level thresholds (high, low, and very low) based on the workbook [31]. These thresholds, detailed in Table S1 in Multimedia Appendix 5, supported focused analysis of individual domains.

#### RIGHT Checklist

We evaluated reporting quality with the RIGHT checklist, which contains 35 items across 7 domains: basic information, background, evidence, recommendations, review and quality assurance, funding and declaration or management of interests, and other information. Three experts assessed each guideline. Items were marked as “Reported” when information was partially or fully present and “Not Reported” when absent.

All experts completed training with the RIGHT Explanation and Elaboration document [20]. A pilot review was followed by consensus discussions with methodological experts. The formal assessment involved 2 rounds, and final results were audited by a methodological expert once agreement was reached.

### Guidelines Appraisal by LLM-Based AI Agents

#### Agent Architecture and Rationale

To support scalable quantitative appraisal of CPGs, we developed an AI agent rather than relying on unguided LLM outputs. The agent used an LLM as its core reasoning engine within a retrieval-augmented generation, retrieving relevant appraisal criteria from a structured knowledge base before each assessment (Figure 1B). This approach grounded model responses in validated reference materials, improving consistency, traceability, and alignment with established appraisal standards.

For AGREE II appraisal, the knowledge base combined the official instrument with the workbook that provides detailed scoring criteria, examples, and explanatory notes. The workbook was originally developed through expert consensus to standardize human appraisal. It first demonstrated its value by improving interrater agreement among human experts, with intraclass correlation coefficients (ICCs) increasing from poor-to-moderate to very good levels. Building on this finding, we examined whether the same structured guidance could similarly improve agreement between AI agents and human experts. The agent was implemented using the LangChain framework, and all reference materials were indexed into a searchable knowledge base using the text-embedding-ada-002 model. During inference, the agent retrieved relevant passages to support each appraisal. Prompts included role definition, stepwise instructions, and in-context examples. Each assessment generated a numerical score (1-7), a confidence rating, and a written justification. For the RIGHT appraisal, we adapted the same framework using the official RIGHT Explanation and Elaboration document as the primary knowledge source. Prompt instructions were iteratively refined to align the agent’s reasoning process with that of human experts.

#### Study Design and Data Source

We tested 2 AI agents against human consensus under 2 conditions: without additional guidance and with a structured guideline appraisal workbook. This design evaluated whether the workbook improved agreement between AI agents and human experts. The 2 experimental factors were the underlying LLM engine (DeepSeek-R1 vs o1-mini) and the knowledge configuration (AGREE II criteria alone vs AGREE II combined with the workbook). Two human experts independently appraised all guidelines, and their interrater agreement served as the human comparison.

To examine whether the effect of structured guidance extended beyond AGREE II, we also evaluated reporting quality using the RIGHT checklist. A baseline AI appraisal arm was constructed using the official RIGHT Explanation and Elaboration document as the sole knowledge source. This comparison allowed us to distinguish instrument-specific effects from broader limitations of LLM-based appraisal and to evaluate the contribution of structured guidance beyond authoritative reference materials alone.

#### AI Agents Assisted Appraisal Framework

The primary outcome was agreement between AI agents and human experts, measured using the ICC (2-way random-effects model, absolute agreement, multiple raters). Cohen κ was calculated as a secondary measure of agreement. To assess explainability, 2 human experts independently evaluated the accuracy and logical consistency of AI-generated justifications using a 5-point Likert scale, ranging from 1 (completely inaccurate) to 5 (fully accurate and logical). Comparisons across experimental conditions focused on the effect of the guideline appraisal workbook.

#### Generalizability and Efficiency Validation

To evaluate whether the AGREE II plus workbook framework generalized beyond rehabilitation and to reduce the possibility of domain-specific overfitting, we performed an independent external validation using 6 CPGs on anterior cruciate ligament (ACL) reconstruction identified from a previous systematic review [32]. Both DeepSeek-R1 and o1-mini appraised these guidelines under the same workbook-supported conditions.

Original AGREE II scores reported in the reference study were used for comparison. AI-generated scores were compared with those of 2 independent human experts and with the published appraisal results. The human experts had methodological expertise but no prior clinical experience in ACL reconstruction and completed independent appraisals without consensus discussion. We calculated ICCs to compare agreement between AI agents and human experts in this unfamiliar clinical setting. We also recorded the time required to complete each AGREE II appraisal, from initial review to final scoring, and compared the mean appraisal time of the AI agents with that of the human experts to quantify efficiency gains. Technical details of the AI pipeline are provided in Multimedia Appendix 6.

### Statistical Analysis

#### Descriptive Analysis

We summarized the number and characteristics of rehabilitation CPGs using counts, percentages, means, and SDs. Data extracted with the standardized template are presented in Table S2 in Multimedia Appendix 5. Geographic distribution was mapped with OpenStreetMap. We also examined trends in covered conditions and publication volume over time. Primary descriptive outcomes were AGREE II domain scores and RIGHT reporting rates.

#### Reliability Analysis

We calculated ICCs to assess interrater reliability for AGREE II and RIGHT evaluations. Standard ICC methods assume interval-level ratings with equal spacing between categories. AGREE II scores, however, often show skewed and asymmetric distributions. To address this, we applied a modified ICC that better reflects the observed score distribution by mathematically mapping the original 7-point ordinal ratings onto a weighted continuous scale. Specifically, this transformation accounts for the unequal psychological distances between rating categories and applies domain-specific penalties for different levels of disagreement before computing the correlation (Document 2 in Multimedia Appendix 5). Agreement was interpreted using established thresholds: ICC ≤0.20 indicated poor agreement; 0.21-0.60 moderate agreement; 0.61-0.80 good agreement; and ≥0.80 very good agreement [33].

#### Univariate Analysis

We explored factors associated with guideline quality through univariate analyses. Variables included publication year, region, institution, funding, target condition, guideline type, publication platform, author number, panel size, evidence level, development method, recommendation formulation method, number of recommendations, use of quality tools, update policy, timing relative to AGREE II publication, external review, version, language, and guideline quality. We applied independent-samples *t* test or the Mann-Whitney *U* test for 2-group comparisons and the Kruskal-Wallis test or 1-way ANOVA for comparisons involving 3 or more groups. All tests were 2-tailed, with *P*<.05 considered statistically significant.

#### Logistic Regression

We used event-constrained multivariable logistic regression to identify characteristics associated with high-quality guidelines. The binary outcome was guideline quality, defined as high quality or not high quality. High-quality guidelines were defined as scoring at least 60% in AGREE II domains 1, 2, 3, 4, and 6; the applicability domain was excluded from this definition. Candidate predictors were first screened using univariate likelihood ratio tests, and variables with *P*<.25 were retained for multivariable analysis (15 of 19 variables). To reduce data sparsity, categories with small sample sizes were combined before model fitting. Because the number of high-quality guidelines was limited relative to the number of candidate predictors, we applied backward elimination at the domain level. At each step, the variable whose removal resulted in the smallest reduction in model fit (likelihood ratio tests *P*>.10) was removed. The final inferential model was restricted to 2 methodologically relevant process variables, recommendation formulation method and external review, yielding 3 model parameters and an events-per-variable ratio of 10.67. A full model including all 19 candidate variables would have required 43 design-matrix parameters for only 32 high-quality events (events per parameter=0.74) and was therefore considered exploratory. Results from the full model are presented in Multimedia Appendix 5 (Table S13). Because only 2 of the 32 high-quality guidelines lacked external review, we additionally fitted the same 3-parameter model using Firth-penalized logistic regression and reported profile-likelihood 95% CIs and penalized likelihood-ratio *P* values. We also performed a sensitivity analysis using the same criteria with a lower quality threshold of 50% [34].

#### Meta-Analysis of Reporting Rates

We carried out meta-analyses to examine how factors such as language and publication timing relative to the RIGHT checklist affected reporting rates. To prevent double counting, we strictly ensured that each included CPG represented an independent development process by excluding translated and duplicate versions. No 2 guidelines represented the same specific version for the same target population, thus ensuring the independence of each “subject” (guideline) in our models. We calculated risk differences (RDs), expressed as proportion differences, and 95% CIs using a random-effects model with the Hartung-Knapp-Sidik-Jonkman (HKSJ) adjustment [35] to provide more robust estimations of the pooled average effects and reduce false positives. Heterogeneity was assessed using the *I*^2^ and τ^2^ statistics. Furthermore, to illustrate the distribution of true effects across different settings, 95% prediction intervals (PIs) were calculated and displayed alongside the CIs in all forest plots. Additionally, small study effects were evaluated visually via funnel plots and quantitatively using the Egger linear regression test, with a predefined significance threshold of *P*<.10.

We did not assess certainty (confidence) in the body of evidence using GRADE (Grading of Recommendations Assessment, Development, and Evaluation) or similar frameworks because this review evaluated the methodological and reporting quality of CPGs rather than the effects of clinical interventions. Therefore, certainty-of-evidence ratings were not applicable to the primary objectives of this review.

All analyses were completed with SPSS (version 25.0; IBM Corp), Python (Python Software Foundation), and R software (R Foundation for Statistical Computing). Statistical significance was assessed using 2-sided *P* values where applicable; for logistic regression, the null value for odds ratios (ORs) was 1, whereas for meta-analyses of RDs/proportion differences, the null value was 0.

## Results

### Study Selection

**Figure 2:**
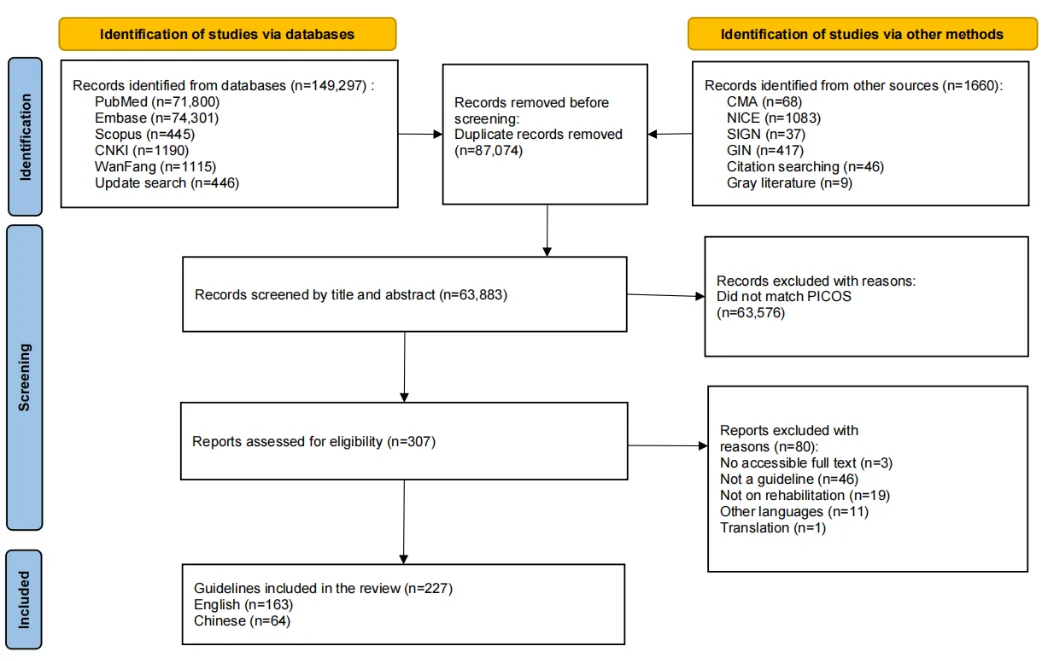
Flowchart of rehabilitation clinical practice guidelines search and selection. CMA: Chinese Medical Association; CNKI: China National Knowledge Infrastructure; GIN: Guidelines International Network; NICE: National Institute for Health and Care Excellence; PICOS: participants, intervention, comparison, outcome, and study design; SIGN: Scottish Intercollegiate Guidelines Network.

The search identified 150,957 records, including 149,297 from electronic databases and 1660 from other sources. After removal of 87,074 duplicates, 63,883 records remained for title and abstract screening. Of these, 63,576 did not meet the PICOS (participants, intervention, comparison, outcome, and study design) criteria and were excluded. We assessed 307 reports in full text. A total of 80 records were excluded at this stage (Table S3 in Multimedia Appendix 5), most often because they were not CPGs (n=46), were unrelated to rehabilitation (n=19), or were published in languages outside the eligibility criteria (n=11). The final review included 227 CPGs, comprising 163 English-language and 64 Chinese-language documents (Figure 2).

### Study Characteristics

The 227 included guidelines varied widely in format, development processes, and geographic origin (Table 1). Most were issued as standard guidelines and were disseminated through both journal and online platforms. Professional societies were responsible for nearly half of the guidelines (n=124, 54.6%), typically focused on specific clinical areas. Government agencies funded 67 (29.5%) guidelines, while guideline societies supported 60 (26.4%). Evidence synthesis was common. A total of 177 (78%) guidelines reported reliance on systematic reviews, and formal consensus (n=78, 34.4%) was reported more often than informal consensus (n=60, 26.4%). GRADE was the most frequently applied evidence grading system (n=93, 41% of guidelines). Regarding scope, 88 (38.8%) guidelines issued 10 or fewer recommendations. Methodological support tools were infrequently cited; only 40 (17.6%) guidelines referenced AGREE II. External review was reported in 106 (46.7%) guidelines, predefined update timelines in 78 (34.4%), and 22 (9.7%) were identified as earlier versions that had been updated.

**Figure 3:**
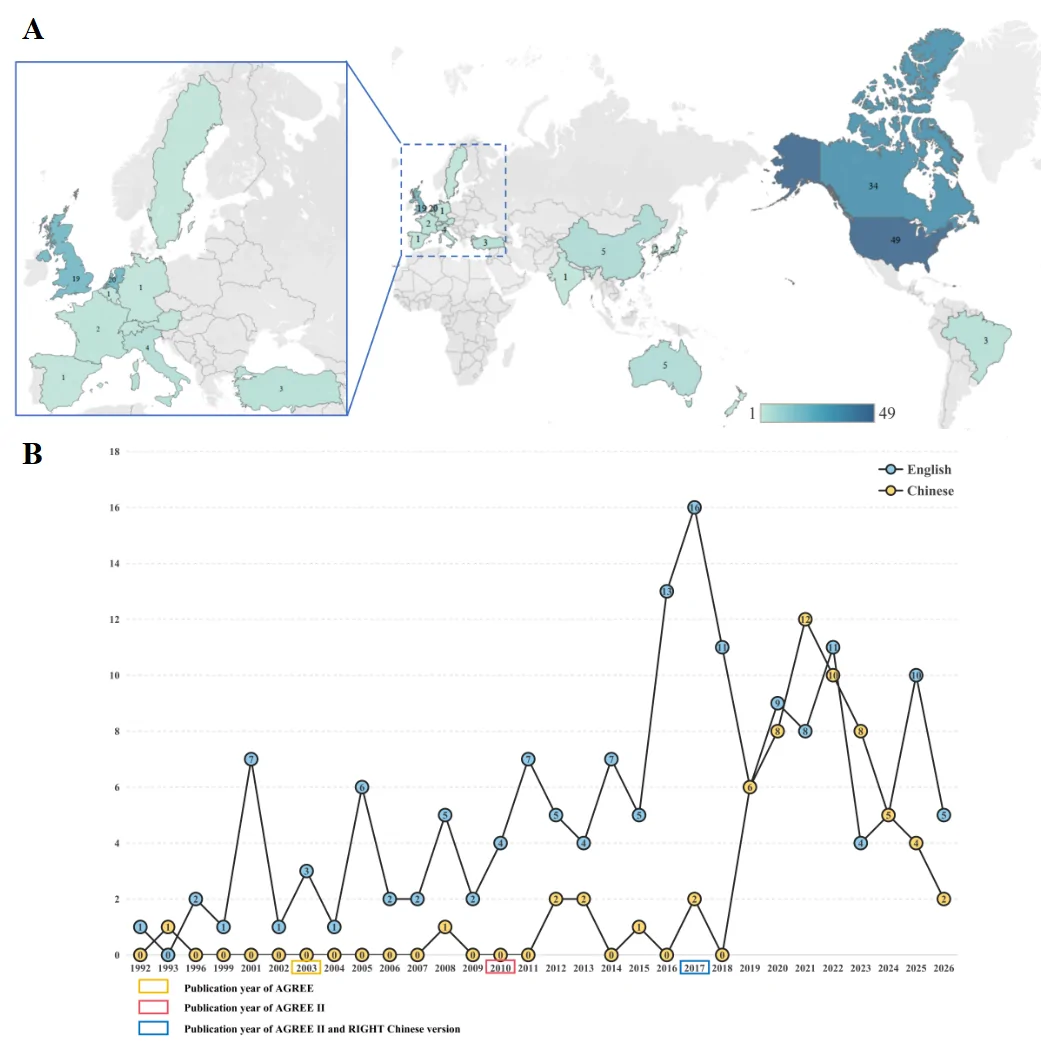
Geographic and temporal distribution of 227 rehabilitation clinical practice guidelines (CPGs). (A) World map of English-language CPGs by country. (B) Annual publication counts for English-language and Chinese-language CPGs. AGREE: Appraisal of Guidelines for Research and Evaluation; RIGHT: Reporting Items for Practice Guidelines in Health Care.

Clear geographic patterns emerged (Figure 3A). English-language guidelines were produced mainly in the United States (n=49), Canada (n=34), the Netherlands (n=20), and England (n=19). Italy (n=4), English-language guidelines from China (n=5), Turkey (n=3), Brazil (n=3), and Australia and New Zealand combined (n=5) each contributed 3 to 5 guidelines. France, Spain, Belgium, Germany, South Korea, Japan, and India each contributed 2 or fewer. A total of 11 guidelines resulted from multinational collaborations.

Temporal trends are shown in Figure 3B. The earliest rehabilitation guideline dated to 1992, followed by the first Chinese guideline in 1993. English-language publications increased gradually over time, while no Chinese guidelines appeared between 1994 and 2007. Chinese-language guideline output remained limited until 2018, followed by a marked increase from 2019 onward. English-language publications also increased during this period, reaching a peak in 2017 (n=16), whereas Chinese-language guidelines peaked in 2021 (n=12).

**Table 1:** Characteristics of the 227 clinical practice guidelines.

Categories and characteristics			Guidelines, n (%)
**Year of publication**			
	1992-2010	39 (17.2)	
	2011-2016	46 (20.3)	
	2017-2020	58 (25.6)	
	2021-2026	84 (37)	
**Region/language**			
	North America	83 (36.6)	
	United States	49 (21.6)	
	Canada	34 (15)	
	Europe	49 (21.6)	
	England	19 (8.4)	
	Netherlands	20 (8.8)	
	Italy	4 (1.8)	
	France	2 (0.9)	
	Spain	1 (0.4)	
	Belgium	1 (0.4)	
	Germany	1 (0.4)	
	Multinational	1 (0.4)	
	Oceania	5 (2.2)	
	Australia and New Zealand	5 (2.2)	
	Asia	74 (32.6)	
	China (English)	5 (2.2)	
	China (Chinese)	64 (28.2)	
	Korea	2 (0.9)	
	Japan	2 (0.9)	
	India	1 (0.4)	
	Transcontinental	3 (1.3)	
	Turkey	3 (1.3)	
	South America	3 (1.3)	
	Brazil	3 (1.3)	
	Combination	10 (4.4)	
**Type of institution [36]**			
	Discipline- or disorder-focused professional organization	124 (54.6)	
	University or hospital	32 (14.1)	
	CPG^a^ creating specialist entity	24 (10.6)	
	Provincial, state, or national health department	9 (4.0)	
	Not stated, combination, other, unclear	38 (16.7)	
**Funding [37]**			
	Government	67 (29.5)	
	Industry	2 (0.9)	
	Guideline society	60 (26.4)	
	Combination	9 (4)	
	Not reported	89 (39.2)	
**Type of guideline [38]**			
	Standard	222 (97.8)	
	Consolidated	1 (0.4)	
	Guidelines produced in response to an emergency or urgent need	4 (1.8)	
	Adapted	0 (0)	
**Publication platform [39]**			
	Journal only	0 (0)	
	online only	19 (8.4)	
	Journal and online	208 (91.6)	
**Number of authors**			
	>20	40 (17.6)	
	11-20	37 (16.3)	
	6-10	51 (22.5)	
	≤5	99 (43.6)	
**Number of panel members**			
	>60	14 (6.2)	
	31-60	47 (20.7)	
	11-30	32 (14.1)	
	≤10	33 (14.5)	
	Not listed	101 (44.5)	
**Disease topic**			
	Cardiac disorders	19 (8.4)	
	Pulmonary disorders	19 (8.4)	
	Spinal cord injury	12 (5.3)	
	Stroke	32 (14.1)	
	Arthritis	24 (10.6)	
	Others	121 (53.3)	
**Evidence grading system [37]**			
	GRADE^b^ system	93 (41)	
	Oxford system	10 (4.4)	
	Other system	77 (33.9)	
	None	47 (20.7)	
**Method for guideline development [34]**			
	Systematic review	177 (78)	
	Consensus or narrative review	11 (4.8)	
	Not mentioned	38 (16.7)	
	Adapted or adopted	1 (0.4)	
**Recommendation methods [34]**			
	Informal consensus	60 (26.4)	
	Formal consensus	78 (34.4)	
	Not mentioned	89 (39.2)	
**Number of recommendations**			
	1-10	88 (38.8)	
	11-20	74 (32.6)	
	21-30	29 (12.8)	
	31-40	12 (5.3)	
	≥41	24 (10.6)	
**Quality tool referral**			
	AGREE II^c^	40 (17.6)	
	RIGHT^d^	5 (2.2)	
	Combination	10 (4.4)	
	Not reported	172 (75.8)	
**External review**			
	Yes	106 (46.7)	
	No	121 (53.3)	
**Defined time to update**			
	Yes	78 (34.4)	
	No	149 (65.6)	
**Language**			
	English	163 (71.8)	
	Chinese	64 (28.2)	
**Version**			
	Old	22 (9.7)	
	New	205 (90.3)	

^a^CPG: clinical practice guideline.

^b^GRADE: Grading of Recommendations Assessment, Development, and Evaluation.

^c^AGREE II: Appraisal of Guidelines for Research and Evaluation II.

^d^RIGHT: Reporting Items for Practice Guidelines in Health Care.

### Quality Assessment

#### Consistency of Appraisal

We assessed interrater reliability using ICCs. As shown in Table S4 in Multimedia Appendix 5, AGREE II scores demonstrated high agreement, with mean ICCs ranging from 0.84 to 0.92. Most domains showed good reliability, while domain 2 (stakeholder involvement) and domain 4 (clarity of presentation) reached excellent reliability. RIGHT checklist assessments showed similar consistency, with ICCs between 0.80 and 0.88 across domains (Table S5 in Multimedia Appendix 5).

**Figure 4:**
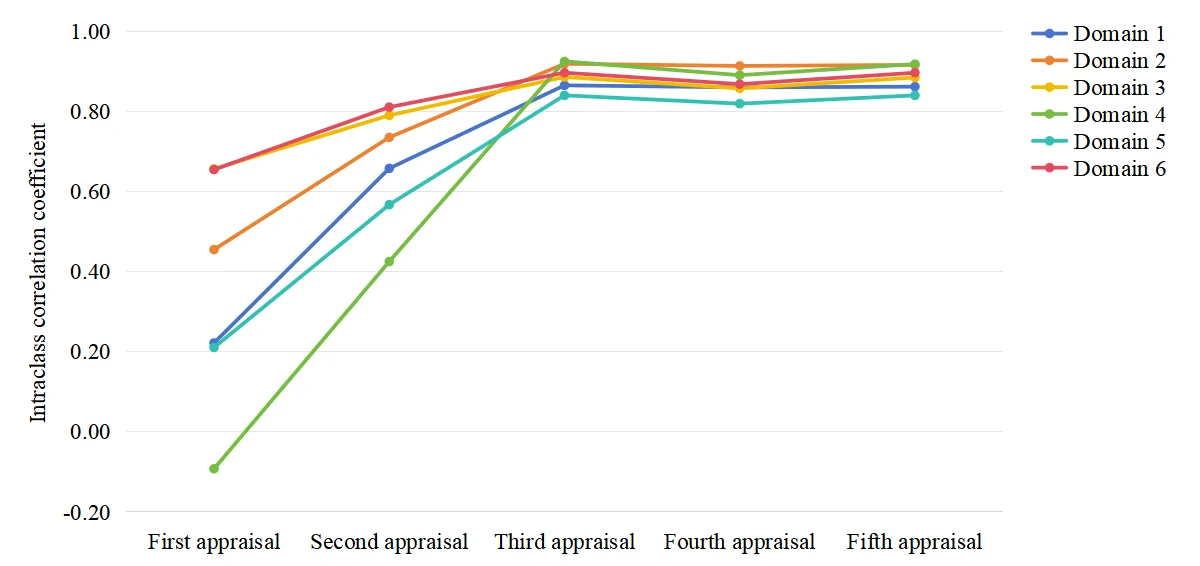
Intraclass correlation coefficient of 5 rounds of appraisal using the Appraisal of Guidelines for Research and Evaluation II instrument.

Figure 4 and Table S6 in Multimedia Appendix 5 show changes in AGREE II agreement across appraisal rounds. Initial ICCs ranged from –0.09 to 0.66, with domain 4 yielding negative values that reflected agreement below chance. After the first consensus meeting, ICCs increased to 0.43-0.81, though several domains remained only moderately consistent. Following implementation of the workbook, ICCs rose to 0.84-0.93 in the third round, with pronounced improvement in domains 1, 4, and 5. These levels of agreement were sustained during the updated search and reassessment phases, with ICCs ranging from 0.84 to 0.92 in the final round.

#### Overall Quality of CPGs

**Figure 5:**
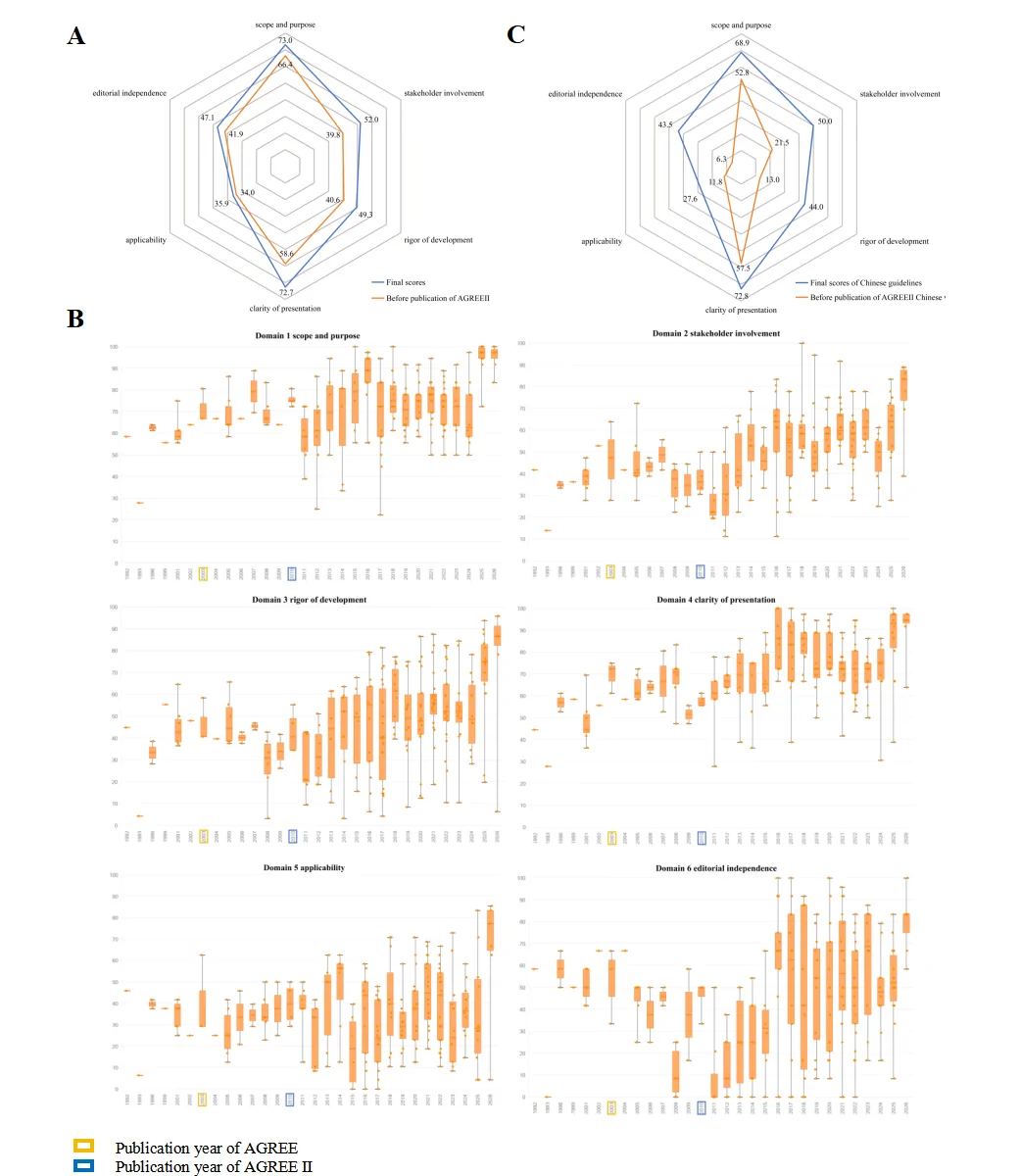
Appraisal of Guidelines for Research and Evaluation (AGREE) II domain scores for 227 rehabilitation clinical practice guidelines (CPGs). (A) Final domain scores. (B) Domain scores for CPGs over the years. (C) Domain scores for Chinese-language guidelines, with stratification by publication before vs after release of the AGREE II Chinese version.

AGREE II domain scores are summarized in Table 2, Table S7 in Multimedia Appendix 5, and Figure 5A. Mean scores were below 60% in 4 domains: stakeholder involvement (mean 52%, SD 17.2%), rigor of development (mean 49.3%, SD 21%), applicability (mean 35.9%, SD 18.8%), and editorial independence (mean 47.1%, SD 27.9%). Higher scores were observed for clarity of presentation (mean 72.7%, SD 15.5%) and scope and purpose (mean 73%, SD 15.2%). Overall, 195 (85.9%) guidelines were classified as low quality, and 32 (14.1%) met criteria for high quality.

Temporal patterns are illustrated in Figure 5B. Guidelines published before the introduction of AGREE II scored lower, with stakeholder involvement at a mean of 39.8% (SD 11%) and applicability at a mean of 34% (SD 11.4%). Figure 5C focuses on Chinese guidelines. Mean scores were below 50% for all domains except clarity of presentation (mean 72.8%, SD 12.6%) and scope and purpose (mean 68.9%, SD 13.9%). The lowest scores appeared in applicability (mean 27.6%, SD 16.7%), followed by rigor of development (mean 44%, SD 20.4%) and editorial independence (mean 43.5%, SD 26.5%). Chinese guidelines published before the release of the Chinese AGREE II version performed particularly poorly, with mean scores below 20% in rigor of development (mean 13%, SD 11.3%), applicability (mean 11.8%, SD 7.7%), and editorial independence (mean 6.3%, SD 12.5%).

**Table 2:** Appraisal of Guidelines for Research and Evaluation II domain scores of included clinical practice guidelines.

	Score (%), minimum-maximum	Score (%), mean (SD)	Score (%), median (IQR)
Domain 1: scope and purpose	22-100	73.0 (15.2)	75 (61-83)
Domain 2: stakeholder involvement	11-100	52.0 (17.2)	53 (39-64)
Domain 3: rigor of development	3-96	49.3 (21.0)	49 (38-64)
Domain 4: clarity of presentation	28-100	72.7 (15.5)	72 (64-83)
Domain 5: applicability	0-85	35.9 (18.8)	38 (23-50)
Domain 6: editorial independence	0-100	47.1 (27.9)	50 (25-67)

#### Reporting Quality of CPGs

**Figure 6:**
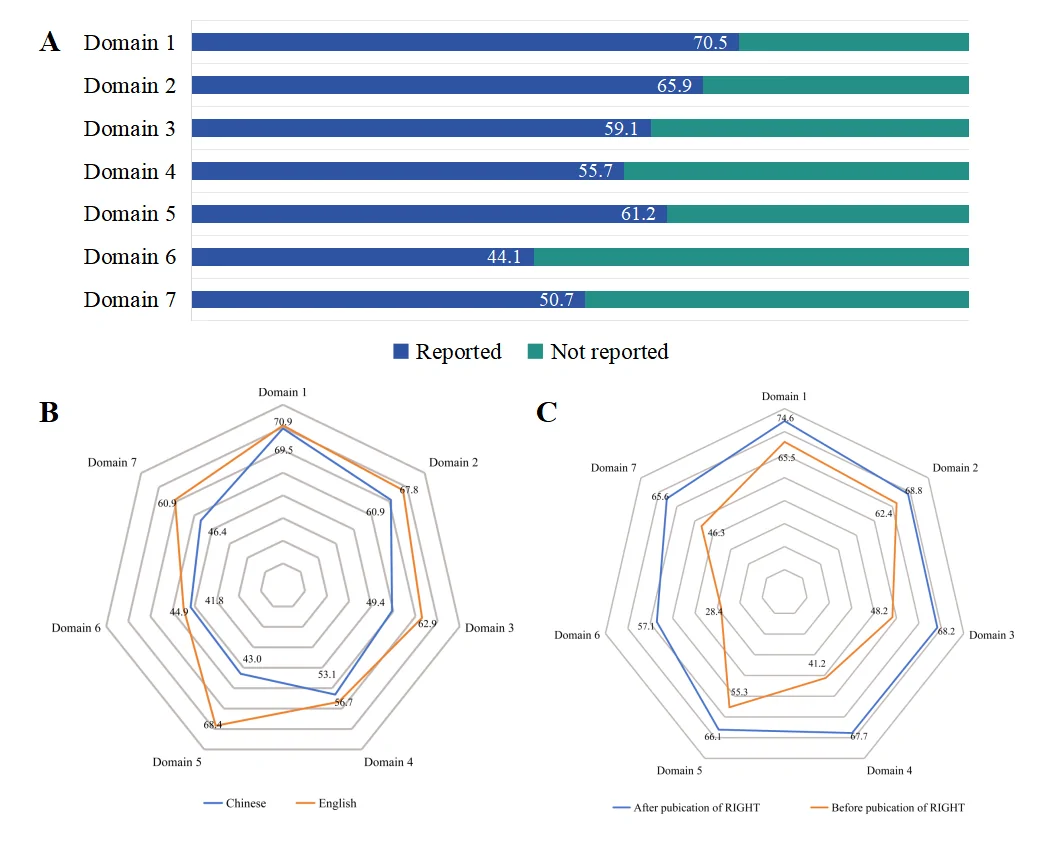
Reporting Items for Practice Guidelines in Health Care (RIGHT) checklist reporting rates for 227 rehabilitation clinical practice guidelines (CPGs). (A) Final domain rates. (B) Language-based comparison of domain reporting rates between English-language and Chinese-language. (C) Temporal comparison of domain reporting rates for CPGs published before vs after the RIGHT checklist release (2017).

RIGHT checklist reporting rates are shown in Tables S8-S9 in Multimedia Appendix 5 and Figure 6. At the item level, reporting was highest for guideline focus (item 1c: 220/227, 96.9%), identification (item 1a: 217/227, 95.6%), and objectives (item 6: 214/227, 94.3%). Reporting was lowest for subgroup descriptions (item 7b: 41/227, 18.1%), publication year (item 1b: 47/227, 20.7%), and intended settings (item 8b: 64/227, 28.2%). Domain-level analysis showed the highest reporting rate in domain 1 (basic information: 70.5%) and the lowest in domain 6 (funding, declaration, and management of interests: 44.1%; Figure 6A).

Language- and time-based subgroup analyses revealed distinct patterns. As shown in Figure 6B, the largest difference between Chinese and English guidelines occurred in domain 5 (review and quality assurance: 25.4%), followed by domain 7 (14.6%) and domain 3 (13.6%). Differences were smallest in domains 1 and 4. Figure 6C shows comparisons before and after release of the RIGHT checklist. Reporting improved most in domains 6 (28.7%), followed by domain 4 (26.5%) and 3 (20.1%), while domains 1 and 2 changed little.

### Factors Associated With Guideline Quality

#### Overview

Table S10 in Multimedia Appendix 5 summarizes guideline characteristics by quality category. High-quality guidelines were more frequent among those published after 2020 (20/84, 23.8%), developed in the Americas (23/86, 26.7%), and supported by systematic reviews (32/177, 18.1%). Higher proportions of high quality were also observed in guidelines that used formal consensus methods (18/78, 23.1%), applied both AGREE II and RIGHT during development (4/10, 40%), underwent external review (30/106, 28.3%), or defined an update schedule (22/78, 28.2%). More high-quality guidelines were published after the release of AGREE II (32/188, 17%).

#### Univariate Analysis

Associations between guideline characteristics and AGREE II domain scores are presented in Table S11 in Multimedia Appendix 5. English-language guidelines scored higher than Chinese-language guidelines in scope and purpose (mean 74.64, SD 15.4 vs mean 68.88, SD 13.9; *P*=.004) and applicability (mean 39.14, SD 18.6 vs mean 27.54, SD 16.7; *P*<.001). Guidelines published between 2021 and 2026 showed higher scores in scope and purpose (*P*=.002), stakeholder involvement (*P*<.001), rigor of development (*P*<.001), clarity of presentation (*P*<.001), and editorial independence (*P*<.001). Regional differences were evident for stakeholder involvement, rigor of development, applicability, and editorial independence. Institutional type was associated with applicability and editorial independence.

Methodological features showed consistent relationships with quality. Guidelines based on systematic reviews scored higher in all domains (*P*<.01). Use of consensus methods was also associated with higher scores in all domains (*P*<.005) except applicability. Reference to AGREE II or RIGHT correlated with higher scores in all domains (*P*<.01). Defined update schedules were linked to higher scores in clarity of presentation (*P*=.006) and all other domains (*P*<.001). External review was associated with higher scores in all domains (*P*<.001). Publication after release of AGREE II was associated with higher scores in scope and purpose, stakeholder involvement, rigor of development, and clarity of presentation.

#### Logistic Regression

Multivariable logistic regression results are shown in Table S12 in Multimedia Appendix 5. Under the primary 60% threshold, 32 of 227 guidelines met the high-quality definition. The parsimonious model included 3 parameters (recommendation method and external review), yielding an events-per-variable ratio of 10.67 and a 5-fold cross-validated area under the curve of 0.75. External review was an associated process characteristic (OR 20.39, 95% CI 4.66-89.27; *P*<.001). This association remained statistically significant in the Firth-penalized sensitivity analysis (OR 16.24, profile-likelihood 95% CI 5.08-82.10; penalized likelihood-ratio *P*<.001). Formal consensus (OR 1.78, 95% CI 0.65-4.87; *P*=.26) and informal consensus (OR 1.17, 95% CI 0.36-3.82; *P*=.80) were not statistically significant in the primary model. The complete 19-domain likelihood-ratio screening table is provided in Table S13 in Multimedia Appendix 5. In the 50% sensitivity analysis, 68 guidelines met the lower-threshold definition and yielded consistent results (Table S14 in Multimedia Appendix 5).

#### Subgroup Analyses

**Figure 7:**
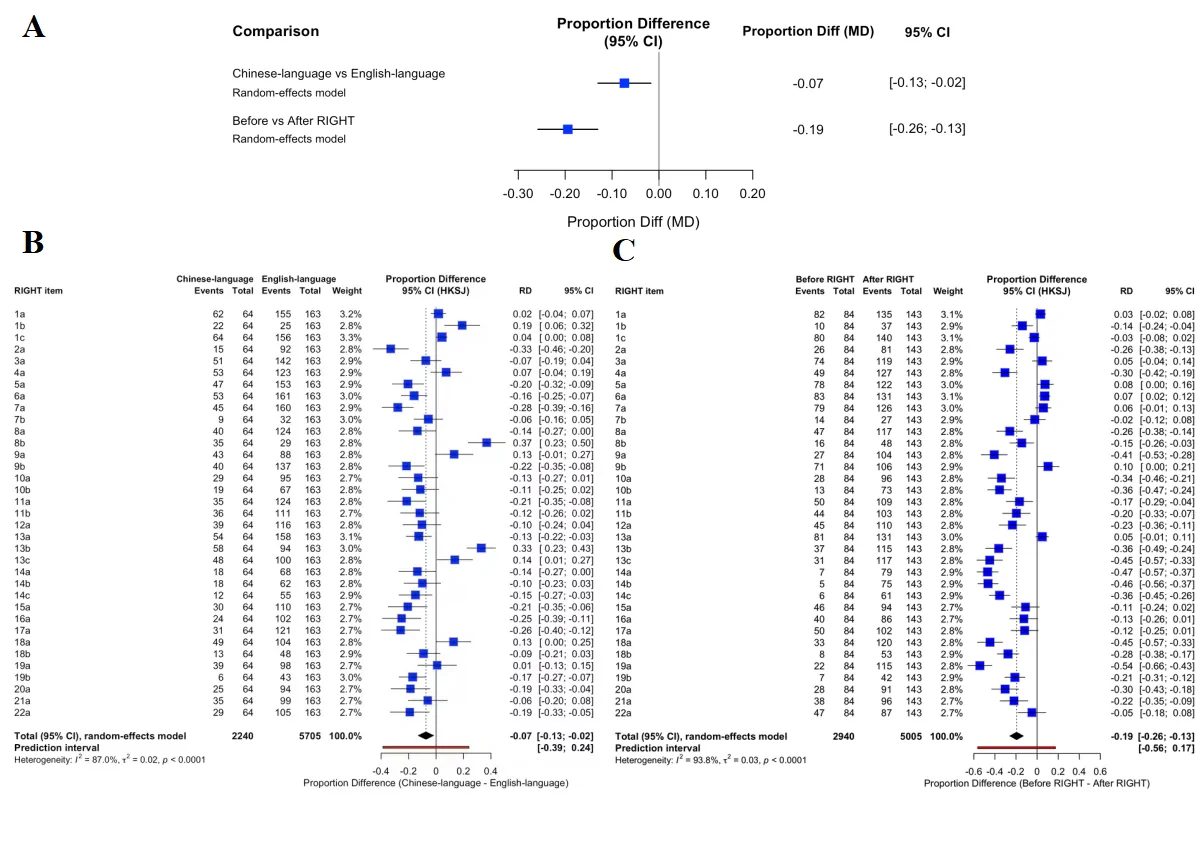
Forest plots of meta-analyses of Reporting Items for Practice Guidelines in Health Care (RIGHT) checklist reporting rates for 227 rehabilitation clinical practice guidelines (CPGs). (A) Overall reporting quality comparison by language (Chinese vs English) and publication timing (before vs after the RIGHT checklist release). (B) Item-level comparisons between Chinese-language and English-language CPGs. (C) Item-level comparisons for CPGs published before vs after the RIGHT checklist release. HKSJ: Hartung-Knapp-Sidik-Jonkman.

Figure 7 shows subgroup comparisons of reporting quality. For the overall comparison between Chinese-language and English-language CPGs, the typical direction across all items indicated a lower reporting rate in Chinese-language guidelines (RD –0.07, 95% HKSJ-adjusted CI –0.13 to –0.02; τ^2^=0.02), while the 95% PI, which illustrates the range of true effects expected in any single future comparison, was wide and crossed the null value (95% PI –0.39 to 0.24; Figure 7B). Egger test suggested possible small study effects in this comparison (Egger test, *P*=.04; Figure S1a in Multimedia Appendix 5).

Similarly, for the comparison before and after the release of the RIGHT checklist, the typical direction across all items showed an improvement (RD –0.19, 95% HKSJ-adjusted CI –0.26 to –0.13; τ^2^=0.03), with the wide PI (95% PI –0.56 to 0.17; Figure 7C). Similarly, the Egger test revealed significant asymmetry in the funnel plot (Egger test; *P*<.001), indicating the presence of small study effects in the temporal comparison (Figure S1b in Multimedia Appendix 5).

### AI Agent Assisted Appraisal Results

#### Workbook Effects on Human-Agent Agreement

**Figure 8:**
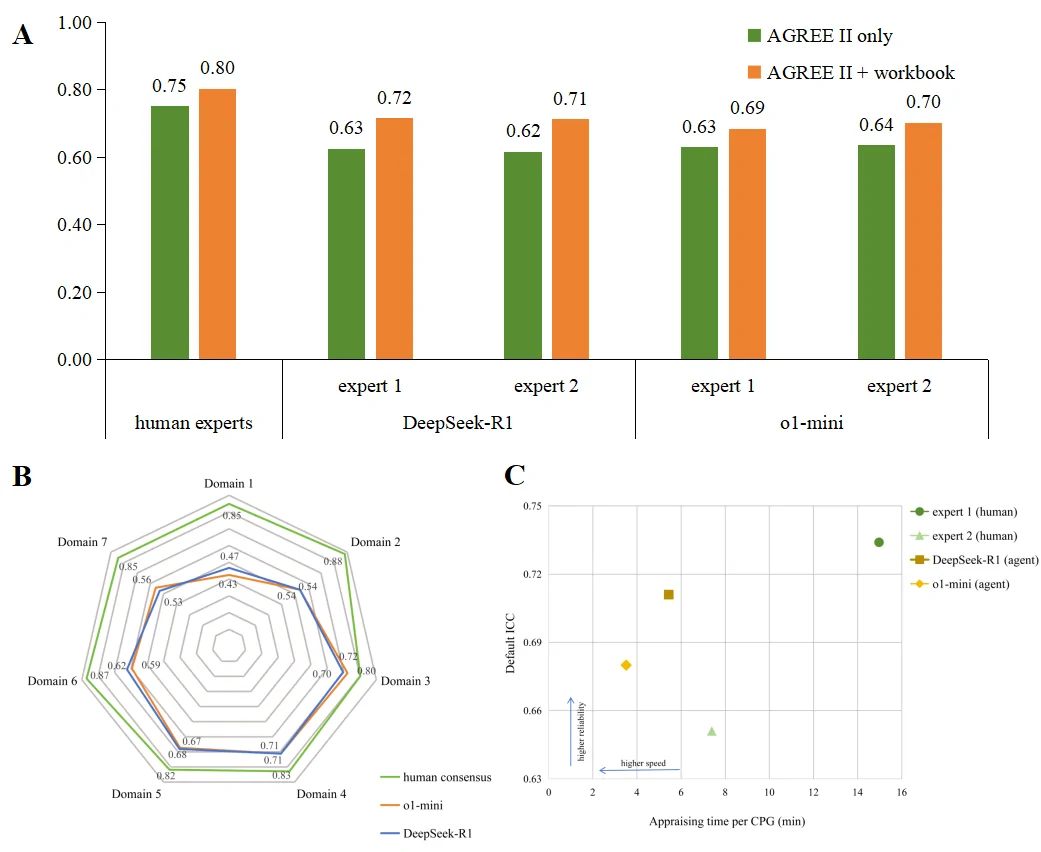
AI agent vs human expert for evaluating clinical practice guidelines (CPGs). (A) Intraclass correlation coefficients (ICC) between agents (DeepSeek-R1 and o1-mini) and human experts without additional guidance and workbook-supported conditions for Appraisal of Guidelines for Research and Evaluation II (AGREE II) scores. (B) ICC between agents (DeepSeek-R1 and o1-mini) and human consensus for Reporting Items for Practice Guidelines in Health Care reporting rates. (C) Efficiency analysis comparing mean appraisal time per guideline and ICC for human experts vs DeepSeek-R1 under workbook-supported conditions. EXP: expert.

The dataset comprised 221 rehabilitation CPGs. Six guidelines were excluded with documented reasons (Table S7 in Multimedia Appendix 6), resulting in 5083 individual scoring items. Agreement between agents and human experts was moderate at baseline. When limited to AGREE II criteria, ICCs ranged from 0.616 to 0.636, remaining below the human-human agreement of 0.752. Incorporating the workbook increased agreement across all configurations (Figure 8A, Table S8 in Multimedia Appendix 6). Using the arithmetic mean across expert 1 and expert 2 consistently for both models, the modified ICC for DeepSeek-R1 rose from 0.613 to 0.709, while o1-mini increased from 0.629 to 0.687. These findings indicate that structured domain guidance strengthened alignment with expert appraisal and improved agreement, although agreement remained below that observed between trained human experts and was not equivalent across all domains.

#### Enhanced Explainability With Workbook-Guided Justifications

The workbook also improved the quality of agent explanations. As shown in Table S9 in Multimedia Appendix 6, explanation quality, as measured by justification completeness and accuracy, showed modest improvement with workbook support for DeepSeek-R1, while o1-mini remained relatively stable. Explanations under workbook guidance cited specific criteria and presented clearer reasoning between evidence and scores, supporting their interpretability in evaluative settings.

#### Appraisal Plateau in the RIGHT Checklist

RIGHT checklist results mirrored baseline AGREE II findings (Figure 8B and Table S10 in Multimedia Appendix 6). Compared with the human consensus (ICC=0.851), DeepSeek-R1 achieved an ICC of 0.621, similar to o1-mini agent (ICC=0.623). While these values did not match the human expert agreement, they mirrored the AGREE II baseline range, suggesting a stable appraisal baseline when agents operate without workbook support. DeepSeek-R1 appraised better in extraction-oriented domains such as basic information (domain 1, ICC=0.467) and funding, declaration, and management of interests (domain 6, ICC=0.624), while o1-mini showed relative strengths in more interpretive domains. This confirms that while AI appraisal of CPGs showed a stable baseline in this dataset, it does not consistently match expert-level reliability across different instruments without dedicated structured guidance.

#### Cross-Domain Generalizability and Efficiency Assessment

Validation using ACL guidelines suggested that the AGREE II plus workbook-supported approach may transfer beyond rehabilitation. Under workbook-supported conditions, agreement between DeepSeek-R1 and human experts (expert 1 and expert 2) yielded ICCs of 0.887 and 0.785, both above the threshold for good reliability (Table S11 in Multimedia Appendix 6). Comparison with the reference showed that DeepSeek-R1 achieved an ICC of 0.711, closely matching expert 1 (ICC=0.734) and exceeding expert 2 (ICC=0.651; Table S12 in Multimedia Appendix 6). These results are encouraging, but because only 6 guidelines from a single clinical domain were included, further validation across diverse specialties is needed before broad generalizability can be claimed.

Efficiency analysis highlighted clear differences between human strategies (Table S13 in Multimedia Appendix 6). Expert 1 emphasized accuracy, averaging 14.97 (SD 2.90) minutes per guideline and achieving higher reliability. Expert 2 favored speed, averaging 7.39 (SD 1.54) minutes, with lower agreement. In contrast, DeepSeek-R1 completed appraisals in a mean of 5.44 (SD 0.16) minutes per guideline while maintaining reliability comparable to the more time-intensive human approach. This pattern suggests that workbook-guided AI appraisal can increase throughput without the decline in accuracy often seen when humans work faster (Figure 8C).

## Discussion

### Principal Findings

This study evaluated both the methodological and reporting quality of rehabilitation CPGs using the AGREE II instrument and the RIGHT checklist. Human agreement remained inconsistent after training based only on the AGREE II manual and tutorial. To address this, we developed a structured guideline appraisal workbook through expert consensus, which markedly improved interrater agreement when applying AGREE II. Despite the growing number of rehabilitation CPGs, methodological quality remained generally low. Applicability and stakeholder involvement were the weakest domains, and English-language guidelines consistently scored higher than Chinese-language guidelines across several domains. External review emerged as an associated process characteristic. For reporting quality, experts achieved good agreement using the official RIGHT Explanation and Elaboration document. Reporting was strongest for basic information but remained limited for funding disclosure and conflicts of interest. Guidelines published after the release of the RIGHT checklist showed better reporting quality than earlier publications.

We also evaluated AI-assisted guideline appraisal to improve appraisal efficiency. Without structured guidance, LLM-based agents showed only moderate agreement with human experts. Agreement increased after introducing the guideline appraisal workbook. External validation using 6 ACL reconstruction guidelines produced similar findings, with good agreement against an external comparison and performance approaching that of experienced methodologists. These findings suggest that structured methodological guidance improves consistency in both human and AI-assisted appraisal while reducing domain-specific variation. Future CPG appraisal is likely to rely on collaboration between human experts and AI systems operating under standardized, expert-developed guidance [26,40].

### The State of Rehabilitation Guidelines

Previous reviews reported that 18% to 38% of CPGs were unsuitable for use even after modification, largely because of poor applicability [16]. Our findings reached the same conclusion. Applicability received the lowest AGREE II score of all 6 domains (mean 35.9%, SD 18.8%), consistent with previous evaluations [17,18]. This recurring weakness suggests that although evidence is often synthesized systematically, guidance on implementation remains insufficient. Many guidelines omit audit criteria, resource implications, cost considerations, and patient-facing materials, limiting translation into routine practice [17,41,42]. The challenge is particularly relevant in rehabilitation. Recent evaluations of oncology, osteoarthritis, and ACL reconstruction guidelines reported the same pattern [9,10,32]. Unlike pharmacological interventions, rehabilitation recommendations require detailed operational information, including the FITT (frequency, intensity, time, and type) principles, therapist qualifications, and equipment requirements, to support implementation [10,43]. Similar findings have also been reported for ACL reconstruction guidelines, where applicability received the lowest AGREE II score (median 29%, IQR 8%-39%), largely because facilitators, barriers, and resource implications were poorly described [32]. The consistency of these findings across different clinical fields suggests that limited applicability reflects a broader weakness in guideline development rather than a problem unique to rehabilitation. Guideline developers appear to place greater emphasis on evidence synthesis than on implementation, leaving many guidelines better suited as evidence summaries than practical tools for clinical care [17,42].

Fewer than half of the included guidelines reported comprehensive systematic review methods or explicit evidence grading. In rehabilitation, where high-quality evidence is often limited, reliance on informal consensus, observed in nearly one-third of guidelines, may reinforce outdated practice. As evidence-based medicine has advanced, confidence in guideline recommendations has become increasingly dependent on transparent development methods [44,45]. Similar concerns have been reported in other fields. A systematic appraisal of 421 noncommunicable disease guidelines found that formal consensus methods were more common among higher-quality guidelines (44/74, 59.5%), although many guidelines still lacked adequate methodological rigor [34]. Likewise, a review of COVID-19 vaccine guidelines reported that only 8 (7.5%) assessed the certainty of evidence and only 28 (26.4%) described their development methods, indicating widespread deficiencies in methodological transparency [39]. Together, these findings suggest that insufficient methodological rigor and continued reliance on informal consensus remain common challenges across guideline development.

Clear differences were observed between English- and Chinese-language guidelines. Previous studies have shown that English-language guidelines consistently achieve higher AGREE II scores than Chinese-language guidelines across multiple domains [46]. Our findings were consistent, with English-language guidelines scoring higher in scope and purpose and applicability. These differences may reflect more established guideline development systems in North America and Europe, where organizations such as NICE and the American Physical Therapy Association (APTA) apply explicit methodological standards and often link funding to implementation planning [17,47]. Although the number of Chinese rehabilitation guidelines has grown rapidly, reporting transparency remains limited. RIGHT assessments showed that funding disclosure and conflict of interest management were frequently omitted, particularly funding information, consistent with previous reports [22]. Broader evaluations suggest that, without independent oversight mechanisms comparable to the Guidelines International Network or WHO guideline committees, guideline development may rely more heavily on expert consensus than on formal methodological processes [34]. Limited disclosure of funding and conflicts of interest may reduce confidence in recommendations and weaken trust in guideline development [48,49]. Similar differences have been documented across other clinical specialties. Chen et al [50] reported that Chinese guidelines generally scored lower than those from Western countries on AGREE II and were less likely to base recommendations on systematic reviews [32]. A systematic evaluation of 17 Chinese hypertension guidelines reached similar conclusions, identifying weaknesses in stakeholder involvement and applicability, with only a small proportion meeting criteria for recommendation without modification [51].

### AI Agents in Guideline Appraisal

Our factorial design showed how structured guidance influenced agreement between AI agents and human experts. When AI agents relied solely on the AGREE II instrument, agreement with human experts was only moderate. Similar findings have been reported in studies evaluating AI-assisted appraisal of medical imaging guidelines, where AI agents had difficulty with nuanced methodological judgments and tended to assign more favorable ratings [24]. Without structured guidance, LLMs may rely on superficial cues, such as the presence of terms like “systematic search,” rather than evaluating whether appropriate methods were actually followed [25,26]. Introducing the guideline appraisal workbook reduced this discrepancy, although agreement remained lower than that achieved between human experts. These findings suggest that structured, domain-specific guidance contributes more to appraisal performance than model selection alone [52,53]. The improvement was greater in models with stronger reasoning capabilities, indicating that structured guidance is most effective when paired with more capable reasoning models [54]. The workbook also improved the quality of AI-generated explanations. During external validation beyond rehabilitation, workbook-guided AI agents completed appraisals substantially faster than human experts while maintaining comparable agreement. This finding suggests that structured guidance can improve efficiency without materially reducing appraisal consistency.

Despite these improvements, AI agents occasionally generated unsupported statements, including incorrect section references and overly confident interpretations of ambiguous text [55,56]. These limitations indicate that AI should support, rather than replace, expert appraisal. A human-in-the-loop workflow remains the most appropriate approach. AI can identify missing information, organize relevant evidence, and prepare preliminary appraisal forms, while human experts verify ratings, interpret complex methodological issues, and resolve ambiguity. This approach combines the efficiency of AI-assisted appraisal with the methodological judgment required for reliable guideline evaluation [26,57].

### Strengths

This study has several strengths. It combines a systematic review of 227 English- and Chinese-language rehabilitation CPGs with an AI-assisted appraisal framework, integrating evidence synthesis, guideline evaluation, and AI-assisted assessment within a single study. The study design allowed us to isolate the effect of the guideline appraisal workbook on agreement between human experts and AI agents, providing a framework that can be readily reproduced in future studies. We also included an independent validation beyond rehabilitation using ACL reconstruction guidelines, showing that the workbook-supported approach may extend to other clinical areas.

### Limitations

Several limitations should be considered. First, the ORs from the logistic regression analysis should be interpreted with caution because the number of high-quality guidelines was limited, although the events-per-variable ratio met the recommended threshold. Second, the validation beyond rehabilitation included only 6 ACL reconstruction guidelines from a single clinical area. Although the findings support the feasibility of applying the workbook-supported approach outside rehabilitation, the small sample limits statistical precision and the generalizability of the results. Third, improved agreement under workbook-supported conditions reflects greater consistency within a shared structured appraisal framework rather than independent external validation. Additional validation across a wider range of clinical specialties is needed. Finally, the AI-assisted appraisal has inherent limitations. Even with workbook support, AI agents occasionally generated unsupported section references, overinterpreted ambiguous text, and were sensitive to the location of relevant information in long documents, consistent with the “lost in the middle” phenomenon [55,56,58].

### Implications

Our findings highlight opportunities to improve how rehabilitation guidelines, and potentially guidelines in other clinical fields, are developed and evaluated. Persistent methodological weaknesses and incomplete reporting, particularly for funding and conflict of interest disclosures, suggest that AGREE II and the RIGHT checklist should be used throughout guideline development rather than only after publication [59-62]. Incorporating these instruments into guideline development, peer review, and clinical appraisal could help identify methodological gaps earlier and promote more transparent and implementable recommendations [19,20,63].

In China, where rehabilitation guideline development is expanding rapidly, strengthening the development process should be a priority. External review was associated with higher guideline quality and represents a practical target for improvement. National organizations could require participation of methodological experts and independent external reviewers during guideline development [64,65]. Establishing a national rehabilitation guideline registry, similar to PROSPERO or GIN, could further improve transparency, reduce unnecessary duplication, and encourage prospective protocol registration. Mandatory disclosure of funding sources and conflicts of interest would address several reporting deficiencies identified in this study [66,67].

Our findings also support the use of AI-assisted guideline appraisal. As the number of CPGs continues to grow, maintaining timely and consistent quality appraisal has become increasingly challenging [68,69]. Health care systems, journal submission platforms, and guideline repositories could incorporate AI agents into appraisal workflows to support initial screening and structured quality assessment, allowing human experts to concentrate on methodological review and clinical judgment [70].

### Conclusion

This study systematically evaluated the methodological and reporting quality of rehabilitation CPGs using both the AGREE II and the RIGHT. Overall guideline quality remained variable, with persistent weaknesses in applicability and stakeholder involvement, particularly among Chinese-language guidelines. AI-assisted appraisal offers a practical approach to improving the efficiency and consistency of guideline evaluation. However, LLM-based appraisal without structured guidance achieved only moderate agreement with human experts. Structured guidance substantially improved agreement and supported AI-assisted appraisal under human oversight. Given the small-sample validation beyond rehabilitation, AI agents should be regarded as decision-support tools rather than replacements for human experts. Future guideline appraisal and evidence synthesis will likely depend on close collaboration between human experts and AI systems operating within standardized, expert-developed appraisal frameworks.

## Appendix

Multimedia Appendix 1 PRISMA 2020 expanded checklist.

Multimedia Appendix 2 PRISMA-S checklist.

Multimedia Appendix 3 PRISMA 2020 abstract checklist.

Multimedia Appendix 4 Tripod-LLM checklist.

Multimedia Appendix 5 The supplementary supporting materials of the systematic review, including the database search strategy and detailed supplementary analysis, etc.

Multimedia Appendix 6 The supplementary supporting materials of the large language model evaluation, including the AI pipeline technical specifications and the detailed large language model evaluation.

## Abbreviations

ACL: anterior cruciate ligament
AGREE II: Appraisal of Guidelines for Research and Evaluation II
APTA: American Physical Therapy Association
CPG: clinical practice guideline
FITT: frequency, intensity, time, and type
GIN: Guidelines International Network
GRADE: Grading of Recommendations Assessment, Development, and Evaluation
HKSJ: Hartung-Knapp-Sidik-Jonkman
ICC: intraclass correlation coefficient
LLM: large language model
NICE: National Institute for Health and Care Excellence
OR: odds ratio
PI: prediction interval
PICOS: participants, intervention, comparison, outcome, and study design
PRISMA: Preferred Reporting Items for Systematic Reviews and Meta-Analyses
PRISMA-S: Preferred Reporting Items for Systematic Reviews and Meta-Analyses Literature Search Extension
PROSPERO: International Prospective Register of Systematic Reviews
RD: risk difference
RIGHT: Reporting Items for Practice Guidelines in Health Care
TRIPOD-LLM: Transparent Reporting of a Multivariable Model for Individual Prognosis or Diagnosis-LLM
WHO: World Health Organization

## Data Availability

All data generated or analyzed during this study are included in this paper and its supplementary information files.
