# Supplementary material for: Improvement of Clinical Practice Guideline Appraisal by Human Experts and AI Agents by Using Structured Guidance: Systematic Review, Meta-Analysis, and Validation Study
Source: J Med Internet Res. 2026 Aug 26;28:e96002. doi: 10.2196/96002 (PMC13559151; doi:10.2196/96002)
Supplement: Supplementary file 5 [file jmir_v28i1e96002_app5.docx]

**Multimedia Appendix 5**

**Systematic Review**

[**Methods.**	3](#_Toc235811527)

[**eSearch Strategy**	3](#_Toc235811528)

[**Table S1. Cut-off of High Quality, Low Quality and Very Low Quality Clinical Practice Guidelines.**	10](#_Toc235811529)

[**Table S2. Data extraction**	11](#_Toc235811530)

[**Document 1. Guideline Appraisal Workbook**	12](#_Toc235811531)

[**Document 2. Code for Modified Intraclass Correlation Coefficient**	41](#_Toc235811532)

[**Screening result**	53](#_Toc235811533)

[**Table S3. Full-text records assessed, reviewed, and excluded from the analysis (n=80) with reasons for exclusion by reference**	53](#_Toc235811534)

[**Quality assessment**	61](#_Toc235811535)

[**Table S4. Interobserver agreement using intraclass correlation coefficient for quality appraisal of CPGs using AGREEⅡ instrument stratified by domain.**	61](#_Toc235811536)

[**Table S5. Interobserver agreement using intraclass correlation coefficient for reporting appraisal of CPGs using RIGHT checklist stratified by domain.**	62](#_Toc235811537)

[**Table S6. Intraclass correlation coefficient of five rounds of appraisal using AGREE II instrument.**	63](#_Toc235811538)

[**Table S7. Scores of 227 CPGs using AGREE Ⅱ Instrument**	64](#_Toc235811539)

[**Table S8. Reporting of 227 CPGs using RIGHT checklist**	76](#_Toc235811540)

[**Table S9. RIGHT reporting rates of is included Clinical Practice Guidelines.**	151](#_Toc235811541)

[**Factors Associated with Guideline Quality**	155](#_Toc235811542)

[**Table S10 The characteristics of CPGs by quality status.**	155](#_Toc235811543)

[**Table S11. Variables related to quality of Clinical Practice Guidelines.**	162](#_Toc235811544)

[**Table S12. Characteristics Associated with High-Quality Clinical Practice Guidelines.**	171](#_Toc235811545)

[**Panel A. Primary standard maximum-likelihood logistic regression**	171](#_Toc235811546)

[**Panel B. Firth-penalized logistic regression**	171](#_Toc235811547)

[**Table S13. 19-domain likelihood-ratio screening and diagnostic results**	173](#_Toc235811548)

[**Table S14. Sensitivity logistic regression analysis using lower 50% cutoffs.**	175](#_Toc235811549)

[**Figure S1. Funnel Plots of Meta-analysis on Reporting Quality. (a) Comparisons between Chinese-language and English-language CPGs. (b) Comparisons between CPGs published before and after the RIGHT checklist release.**	176](#_Toc235811550)

**Methods.**

**eSearch Strategy**

**PUBMED** June 16, 2026

| 42 | 27 AND 41 |
| --- | --- |
| 41 | 36 OR 40 |
| 40 | 37 OR 38 OR 39 |
| 39 | "Practice Guideline"[Publication Type] |
| 38 | "Practice Guidelines as Topic"[MeSH Terms] |
| 37 | "Guideline"[Publication Type] |
| 36 | 28 OR 29 OR 30 OR 31 OR 32 OR 33 OR 34 OR 35 |
| 35 | "Practice Guideline"[Title/Abstract] |
| 34 | "guide*"[Title/Abstract] |
| 33 | "guideline*"[Title/Abstract] |
| 32 | "guidance*"[Title/Abstract] |
| 31 | "Guideline"[Title/Abstract] |
| 30 | "guidelines"[Title/Abstract] |
| 29 | "Clinical Guidelines"[Title/Abstract] |
| 28 | "recommendation*"[Title/Abstract] |
| 27 | 4 OR 20 OR 26 |
| 26 | 21 OR 22 OR 23 OR 24 OR 25 |
| 25 | "physical therapy"[Title/Abstract] |
| 24 | "exercise"[Title/Abstract] |
| 23 | "kinesitherapy"[Title/Abstract] |
| 22 | "physiotherap*"[Title/Abstract] |
| 21 | "physiatrics"[Title/Abstract] |
| 20 | 5 OR 6 OR 7 OR 8 OR 9 OR 10 OR 11 OR 12 OR 13 OR 14 OR 15 OR 16 OR 17 OR 18 OR 19 |
| 19 | "occupational therapy"[Title/Abstract] |
| 18 | "psychology rehabilitation"[Title/Abstract] |
| 17 | "pediatric rehabilitation"[Title/Abstract] |
| 16 | "aerobic walking programs"[Title/Abstract] |
| 15 | "bibliotherapy"[Title/Abstract] |
| 14 | "language therapy"[Title/Abstract] |
| 13 | "speech therapy"[Title/Abstract] |
| 12 | "music therapy"[Title/Abstract] |
| 11 | "recreation therapy"[Title/Abstract] |
| 10 | "dance therapy"[Title/Abstract] |
| 9 | "animal assisted therapy"[Title/Abstract] |
| 8 | "assistive technology"[Title/Abstract] |
| 7 | "assistant technology"[Title/Abstract] |
| 6 | "hydrotherapy"[Title/Abstract] |
| 5 | "electric stimulation therapy"[Title/Abstract] |
| 4 | 1 OR 2 OR 3 |
| 3 | "Habilitation"[Title/Abstract] |
| 2 | "Rehabilitation"[Title/Abstract] |
| 1 | "Rehabilitation"[MeSH Terms] |

Embase June 16, 2026

| 11 | 3 AND 10 |
| --- | --- |
| 10 | 6 OR 9 |
| 9 | 7 OR 8 |
| 8 | 'physiotherapy':ab,ti OR 'physical therapy':ab,ti OR 'exercise':ab,ti OR 'kinesitherapy':ab,ti OR 'aerobic walking programs':ab,ti |
| 7 | 'occupational therapy':ab,ti OR 'psychology rehabilitation':ab,ti OR 'pediatric rehabilitation':ab,ti OR 'bibliotherapy':ab,ti OR 'language therapy':ab,ti OR 'speech therapy':ab,ti OR 'music therapy':ab,ti OR 'recreation therapy':ab,ti OR 'dance therapy':ab,ti OR 'animal assisted therapy':ab,ti OR 'assistive technology':ab,ti OR 'assistant technology':ab,ti OR 'hydrotherapy':ab,ti OR 'electric stimulation therapy':ab,ti |
| 6 | 4 OR 5 |
| 5 | 'functional readaptation' OR 'medical rehabilitation' OR 'readaption' OR 'readjustment' OR 'rehabilitation concept' OR 'rehabilitation engineering' OR 'rehabilitation potential' OR 'rehabilitation process' OR 'rehabilitation program' OR 'rehabilitation programme' OR 'rehabilitation, medical' OR 'rehabilitative treatment' OR 'resocialisation' OR 'resocialisation therapy' OR 'resocialization' OR 'resocialization therapy' OR 'revalidation' OR 'rehabilitation' |
| 4 | 'rehabilitation'/exp |
| 3 | 1 OR 2 |
| 2 | 'clinical practice guideline*':ab,ti OR 'guideline*':ab,ti OR 'practice guideline*':ab,ti OR 'guide*':ab,ti OR 'recommendation*':ab,ti |
| 1 | 'practice guideline'/exp |

Scopus June 16, 2026

| 6 | 1 AND 5 |
| --- | --- |
| 5 | 2 OR 3 OR 4 |
| 4 | TITLE-ABS-KEY( physiotherapy OR physical AND therapy OR exercise OR kinesitherapy OR aerobic AND walking AND programs ) |
| 3 | TITLE-ABS-KEY( occupational AND therapy OR psychology AND rehabilitation OR pediatric AND rehabilitation OR bibliotherapy OR language AND therapy OR speech AND therapy OR music AND therapy OR recreation AND therapy OR dance AND therapy OR animal AND assisted AND therapy OR assistive AND technology OR assistant AND technology OR hydrotherapy OR electric AND stimulation AND therapy ) |
| 2 | TITLE-ABS-KEY ( (functional AND readaptation OR medical AND rehabilitation OR readaption OR readjustment OR rehabilitation AND concept OR rehabilitation AND engineering OR rehabilitation AND potential OR rehabilitation AND process OR rehabilitation AND program OR rehabilitation AND programme OR rehabilitation AND medical OR rehabilitative AND treatment OR resocialisation OR resocialisation AND therapy OR resocialization OR resocialization AND therapy OR revalidation OR rehabilitation ) |
| 1 | TITLE-ABS-KEY ( clinical practice guideline OR practice guideline OR guide OR recommendation ) |

**CNKI database** June 16, 2026

(SU='康复' OR SU='物理治疗' OR SU='运动' OR SU='运动疗法' OR SU='作业治疗' OR SU='有氧运动' OR SU='言语治疗' OR SU='辅助技术') AND (TI='指南' OR TI='推荐意见')

**WanFang database** June 16, 2026

题名或关键词:(“康复” or “物理治疗” or “运动” or “运动疗法” or “作业治疗” or “有氧运动” or “言语治疗” or “辅助技术”) and 题名:(“指南”or “推荐意见”)

**Other sources in free keyword format (CMA, NICE, SIGN and GIN)**

(clinical practice guideline OR practice guideline OR guide OR recommendation) AND ((functional readaptation OR medical rehabilitation OR readaption OR readjustment OR rehabilitation concept OR rehabilitation engineering OR rehabilitation potential OR rehabilitation process OR rehabilitation program OR rehabilitation programme OR rehabilitation medical OR rehabilitative treatment OR resocialisation OR resocialisation therapy OR resocialization OR resocialization therapy OR revalidation OR rehabilitation) OR (occupational therapy OR psychology rehabilitation OR pediatric rehabilitation OR bibliotherapy OR language therapy OR speech therapy OR music therapy OR recreation therapy OR dance therapy OR animal assisted therapy OR assistive technology OR assistant technology OR hydrotherapy OR electric stimulation therapy) OR (physiotherapy OR physical therapy OR exercise OR kinesitherapy OR aerobic walking programs))

**Grey Literature**

rehabilitation guidelines

(rehabilitation OR physical therapy OR excercise OR occupational therapy OR speech therapy) AND (guideline OR recommendation OR practice guideline OR clinical practice guideline OR guide)

**Table S1. Cut-off of High Quality, Low Quality and Very Low Quality Clinical Practice Guidelines.**

|  | Domain 1  Scope and Purpose (%) | Domain 2  Stakeholder Involvement (%) | Domain 3  Rigor of Development (%) | Domain 4  Clarity of Presentation (%) | Domain 5  Applicability (%) | Domain 6  Editorial Independence (%) |
| --- | --- | --- | --- | --- | --- | --- |
| High | 100 | 100 | 100 | 100 | 100 | 100 |
|  | 72 | 72 | 67 | 67 | 58 | 83 |
| Low | 71 | 71 | 66 | 66 | 57 | 82 |
|  | 33 | 28 | 27 | 33 | 17 | 50 |
| Very Low | 32 | 27 | 26 | 32 | 16 | 49 |
|  | 0 | 0 | 0 | 0 | 0 | 0 |

**Table S2. Data extraction**

| ID | Title | Language | Year of Publication | Type of Institution | Funding | Author | Region | Publication Platform | Disease Topic | Evidence Grading System | Type of Guideline | No. of Authors | No. of Panel Members | Method for Guideline Development | Recommendation Methods | No. of Recommendations | Quality Tool Referral | Defined Time to Update | External Review | Old Version |
| --- | --- | --- | --- | --- | --- | --- | --- | --- | --- | --- | --- | --- | --- | --- | --- | --- | --- | --- | --- | --- |
| 1 | XX guideline |  |  |  |  |  |  |  |  |  |  |  |  |  |  |  |  |  |  |  |

**Document 1. Guideline Appraisal Workbook**

**Guideline appraising Workbook**

**Basic Standards:**

**7: Meets all criteria**

**5-6: Essentially meets the criteria**

**3-4: Missing key entries**

**1-2: Missing descriptions**

**Note.** Document 1 contains selected excerpts, figures, tables, and screenshots from previously published clinical practice guidelines. These materials are included solely as illustrative calibration examples to demonstrate the application of specific AGREE II appraisal criteria and to support consistent scoring by human experts and AI agents. They are not studies included in this systematic review, do not constitute data analyzed in this study, and do not represent the literature search strategy used for this review. Each external figure or screenshot identifying its original source and its specific purpose within the Guideline Appraisal Workbook.

**Domain 1. Scope and Purpose**

1. **The overall objective(s) of the guideline is (are) specifically described.**

Main location: The beginning section of the guideline

**High quality: 5-7**

Example: This clinical practice guideline (CPG) is intended to provide recommendations to improve mobility, function, and qualityof-life (QOL) outcomes using an ankle-foot orthosis (AFO) or functional electrical stimulation (FES) for individuals with poststroke hemiplegia who have decreased lower extremity motor control that impacts body function and structure, activity, and participation.

**Low quality: 3-4**

Example: The purpose of this CPG is to help improve the physical therapist management of individuals with PD based on the current best evidence.

**Very low quality: 1-2**

Example: No relevant description.

**2.The health question(s) covered by the guideline is (are) specifically described.**

Main location: The beginning section of the guideline, search strategy, and summary table

**High quality: 6-7**

Example: In patients with Parkinson’s, are stretching/flexibility interventions more effective than no/other treatments in affecting patient outcomes?

**Low quality: 3-5**

Example: We have sought to provide visibility to broader rehabilitation aspects based on the intervention concepts proposed in the International Classification of Functioning, Disability, and Health. The rehabilitation strategies included in this guideline cover the different stroke phases: hyper-acute (0-24 hours), acute (1-7 days), early subacute (7 days-3 months), late subacute (3-6 months), and chronic phases (> 6 months). Most studies in stroke rehabilitation include participants over the age of 18 years. In clinical practice, the same interventions are used for all ages. Therefore, the Recommendations of this guideline can be applied to all individuals after a stroke. In addition, these guidelines also aim to highlight the issues of accessibility and palliative care.

**Very low quality: 1-2**

Example: Health issues not specified.

**3.The population (patients, public, etc.) to whom the guideline is meant to apply is specifically described.**

Main location: The beginning section of the guideline

**High quality: 5-7**

Example: This CPG addresses the management of adult idiopathic, typical PD. It is not intended to address management of individuals with atypical Parkinsonism disorders or other neurodegenerative conditions. Most studies reviewed include individuals in the early to mid-stages of PD as measured by Hoehn&Yahr (H&Y) stages 1 to 3. Recommendations may not generalize to those in the advanced H&Y stages 4 to 5 of the disease.

**Low quality: 3-4**

Example: The purpose of this paper is to describe the recommendations on the diagnostics and treatment of the revised clinical practice physical therapy guideline for patients with RA.

**Very low quality: 1-2**

Example: Target population not specified.

**Domain 2. Stakeholder Involvement**

**4. The guideline development group includes individuals from all relevant professional groups.**

Main location: The beginning section of the guideline, acknowledgments, and guideline development group composition paragraph

**High quality: 6-7**

Example:

External figure 1. Illustrative example for AGREE II Item 4, showing high quality. Source: Osborne JA, Botkin R, Colon-Semenza C, DeAngelis TR, Gallardo OG, Kosakowski H, et al. Physical Therapist Management of Parkinson Disease: A Clinical Practice Guideline From the American Physical Therapy Association. Phys Ther. 2022 Apr 1;102(4). PMID: 34963139. doi: 10.1093/ptj/pzab302.

**Low quality: 2-5**

Example:

External figure 2. Illustrative example for AGREE II Item 4, showing low quality. Source: 许正伟 郝, 程黎明，贺宝荣. 骨质疏松性椎体压缩骨折椎体强化术后康复治疗指南（2022版）. 中华创伤杂志. 2022;38(11):961-72. doi: 10.3760/cma.j.cn501098-20221020-00696.

**Very low quality: 1**

Example: No description of individuals from all relevant professional groups.

5. **The views and preferences of the target population (patients, public, etc.) have been sought.**

Main location: Methodology section

**High quality: 5-7**

Example: An individual with PD participated in the development of this CPG through the peer-review process. The reviewer provided important feedback on the draft from the perspective of a physical therapy user and commented on the clarity and feasibility of implementing the recommendations. The GDG took the reviewer’s feedback into consideration in making any necessary edits to the CPG.

**Low quality: 3-4**

Example: For therapeutic interventions, the recommendations were formulated based on the GRADE Evidence-to-Decision framework (Alonso-Coello et al., 2016), including a discussion on the balance between benefits and harms, the quality of the evidence, the values and preferences of patients and clinicians, and feasibility, equity, and acceptability of the recommendations.

**Very low quality: 1-2**

Example: Did not specify the collection of target population views.

6. **The target users of the guideline are clearly defined.**

Main location: The beginning section of the guideline

**High quality: 5-7**

Example: This CPG is intended to be used by physical therapists, and physical therapist assistants under the direction of physical therapists, for the management of individuals with PD. Physical therapists are health care professionals who help individuals maintain, restore, and improve movement, activity, and functioning to enable optimal performance and enhance health, well-being, and quality of life. Neurologists, adult primary care clinicians, geriatricians, rehabilitation medicine provider, nurse practitioners, physician assistants, occupational therapists, speech language pathologists, and other health care professionals who routinely see individuals with PD in various practice settings also may benefit from this guideline. This guideline is not intended for use as an insurance benefit determination document.

**Low quality: 3-4**

Example: The target users of these guidelines are physical therapists, physiatrists, orthopedic surgeons, rheumatologists, family physicians, and neurologists.

**Very low quality: 1-2**

Example: Did not specify the guideline users.

**Domain 3. Rigour of Development**

7. **Systematic methods were used to search for evidence.**

Main location: Methodology section, literature search strategy, and attachments

**High quality: 6-7**

Example: The medical librarian conducted a comprehensive search of PubMed, Embase, and the Cochrane Central Register of Controlled Trials based on key terms and concepts from the PICOT questions. Bibliographies of relevant systematic reviews were hand searched for additional references. All databases were last searched on June 16,2020, with limits for publication dates from 1994 through 2020, English language, and only randomized controlled trials. The PICOT questions used to define the literature search and inclusion criteria, and the literature search strategy used to develop this CPG, can be found in Supplementary Appendix 2.

External figure 3. Illustrative example for AGREE II Item 7, showing high quality. Source: Osborne JA, Botkin R, Colon-Semenza C, DeAngelis TR, Gallardo OG, Kosakowski H, et al. Physical Therapist Management of Parkinson Disease: A Clinical Practice Guideline From the American Physical Therapy Association. Phys Ther. 2022 Apr 1;102(4). PMID: 34963139. doi: 10.1093/ptj/pzab302.

**Low quality: 3-5**

Example: A structured literature search was developed based on the sensitive search strategy for RCTs recommended by the Cochrane Collaboration1 and modifications proposed by Haynes et al. The search strategy was expanded to identify case control, cohort, and nonrandomized studies. The search was conducted in the electronic databases of MEDLINE, EMBASE, Current Contents, CINAHL, and the Cochrane Controlled Trials Register up to July 1, 2000.

**Very low quality: 1-2**

Example: Did not describe the literature search method.

8. **The criteria for selecting the evidence are clearly described.**

Main location: Methodology section, literature search, and attachments

**High quality: 5-7**

Example:

External figure 4. Illustrative example for AGREE II Item 8, showing high quality. Source: Moore JL, Potter K, Blankshain K, Kaplan SL, OʼDwyer LC, Sullivan JE. A Core Set of Outcome Measures for Adults With Neurologic Conditions Undergoing Rehabilitation: A CLINICAL PRACTICE GUIDELINE. J Neurol Phys Ther. 2018 Jul;42(3):174-220. PMID: 29901487. doi: 10.1097/npt.0000000000000229.

**Low quality: 2-4**

Example: All 26 studies were randomized controlled trials and only included patients that were clinically diagnosed with knee osteoarthritis.

**Very low quality: 1**

Example: Did not describe the criteria for selecting evidence.

9. **The strengths and limitations of the body of evidence are clearly described.**

Main location: Methodology section, evidence grading table

**High quality: 5-7**

Example: For assessing the methodological quality of articles, AMSTAR 2.0, GRADE system used for systematic reviews, Cochrane risk of bias (RoB) used for randomized controlled trials (RCTs), and the RoB Assessment tool for non-RCTs. Two researchers evaluated the quality of one article. In case of disagreement during the assessment of the methodological quality of the article, consensus was reached after discussion or the final decision was made by the third researcher's arbitration. Data obtained in the search were accepted as evidence of the studies' populations (human cases of ACS), designs, and methods (sample size, blinding, and data analysis), results (objectivity of results and consistency throughout studies), language (Korean and English), and applicability to Korea.

**Low quality: 3-4**

Example: The final step entailed the GDG’s assessment of the identified risks of bias and relative importance of those risks to the procedures or specific outcome of interest to designate the article into one of 4 quality ratings: (1) high quality, (2) acceptable quality, (3) low quality, and (4) unacceptable quality. If a study was deemed as unacceptable quality, it was removed from consideration for inclusion for recommendations related to that area.

**Very low quality: 1-2**

Example: Did not describe the strength and limitations of the evidence.

10. **The methods for formulating the recommendations are clearly described.**

Main location: Paragraph on forming recommendation suggestions

**High quality: 5-7**

Example: GDG members agreed on the strength of every recommendation. Recommendations were approved and adopted when a majority of 60% of the GDG voted to approve. All recommendations received 100% agreement among the quorum of the voting GDG. No disagreements were recorded during recommendation voting. When changes were made to the strength of a recommendation based on the magnitude of benefit or potential risk, harm, or cost, the GDG voted and provided an explanation in the rationale.

**Low quality: 3-4**

Example: In four face-to-face meetings of the guideline panel and in one face-to-face and three digital meetings of the review group over an 18-month period, the results of the (systematic/narrative) literature reviews were presented. For therapeutic interventions, the recommendations were formulated based on the GRADE Evidence-toDecision framework, including a discussion on the balance between benefits and harms, the quality of the evidence, the values and preferences of patients and clinicians, and feasibility, equity, and acceptability of the recommendations. Discussions were structured using an Evidence-to-Decision form (Appendix D), leading to strong or conditional recommendations in favor of or against the intervention or to a neutral recommendation .

**Very low quality: 1-2**

Example: Did not describe the method of forming recommendation suggestions.

11. **The health benefits, side effects, and risks have been considered in formulating the recommendations.**

Main location: Methodology section, discussion, recommendation suggestions section

**High quality: 4-7**

Example: When changes were made to the strength of a recommendation based on the magnitude of benefit or potential risk, harm, or cost, the GDG voted and provided an explanation in the rationale.

Benefits are as follows:

• Improvements in strength/power

• Improvements in nonmotor symptoms (anxiety, cognition, depression)

• Reductions in motor disease severity

• Improvements in activities (gait speed, balance, mobility, stability)

• Improvements in quality of life

• Reduction in fall rate

Risk, harms, and/or costs are as follows:

• There are 6 studies that found no significant difference in adverse events with resistance training compared with control or another active condition. In these studies, adverse events included strains and sprains, delayed onset muscle soreness, fatigue, cardiovascular events, pain, and falls. In 2 studies, hospitalizations and deaths occurred that were deemed unrelated to participation in these studies. In 1 study, injurious falls were reported; however, there were similar rates of injurious falls in the experimental group (progressive resistance strength training and movement strategy training) and the control group (education-based life skills training).

Benefit-harm assessment: The balance of the benefits versus risk, harms, or cost overwhelmingly supports this recommendation.

**Low quality: 2-3**

Example: For therapeutic interventions, the recommendations were formulated based on the GRADE Evidence-to-Decision framework (Alonso-Coello et al., 2016), including a discussion on the balance between benefits and harms, the quality of the evidence, the values and preferences of patients and clinicians, and feasibility, equity, and acceptability of the recommendations.

**Very low quality: 1**

Example: Did not specify that the benefits, side effects, and dangers to health were considered when forming recommendation suggestions.

12. **There is an explicit link between the recommendations and the supporting evidence.**

Main location: Methodology section, discussion, recommendation suggestions, and evidence charts

**High quality: 5-7**

Example: Judging the quality of evidence is only a stepping stone toward arriving at the strength of a CPG recommendation. The operational definitions for the quality of evidence are listed in Table 2, and the rating of the magnitude of benefits versus risk, harms, and cost is provided in Table 3. The strength of recommendation (Tab. 4) also considers the quality, quantity, and trade-off between the benefits and harms of a treatment, the magnitude of a treatment’s effect, and whether there are data on critical outcomes. Table 5 addresses how to link the assigned grade with the language of obligation of each recommendation.

Physical therapists should implement resistance training to reduce motor disease severity and improve strength, power, nonmotor symptoms, functional outcomes, and quality of life in individuals with PD. Evidence Quality: high; Recommendation Strength: strong.

Action Statement Profile

Aggregate Evidence Quality: 19 high-quality studies and 28 moderate-quality studies.

Strength and power

Physical therapists should implement resistance training programs that are progressive in nature. Benefits were observed whether resistance training was carried out alone or as part of a multimodal program to improve strength and power in individuals with PD. There were 3 high-quality and 3 moderate-quality studies that favor resistance training compared with control to improve strength and power.

**Low quality: 2-4**

Example: It is recommended to perform oropharyngeal care and health assessment in patients who had a stroke, as well as a routine assessment of swallowing ability before oral intake (Class I recommendation, Level B evidence).

**Very low quality: 1**

Example: There is no connection between the recommendation suggestions and the supporting evidence.

13. **The guideline has been externally reviewed by experts prior to its publication.**

Main location: Methodology section, acknowledgments, expert composition

**High quality: 5-7**

Example: Following the formation of a final draft, the CPG draft was subjected to a 3-week peer review for additional input from external content experts and stakeholders. More than 250 comments from 12 societies were collected via an electronic structured review form. All peer reviewers were required to disclose any potential conflicts of interest, which were recorded and, as necessary, addressed. After modifying the draft in response to peer review, the CPG was subjected to a 2-week public comment period. Commenters consisted of the APTA Board of Directors (Board), the APTA Scientific and Practice Affairs Committee, all relevant APTA sections and academies, stakeholder organizations, and the physical therapy community at large. More than 47 public comments were received. Revisions to the draft were made in response to relevant comments.

**Low quality: 3-4**

Example: The recommendations and underlying descriptions formed the basis of a draft guideline, which was externally reviewed by seven physical therapists and 14 stakeholder organizations participating in the guideline or review panels (Appendix A). Based on their comments, revisions were made to the draft guideline, which then resulted in the final document.

**Very low quality: 1-2**

Example: Did not specify that it underwent expert review.

14. **A procedure for updating the guideline is provided.**

Main location: Methodology section, final paragraph

**High quality: 5-7**

Example: This CPG represents a cross-sectional view of current management strategies and may become outdated as new evidence becomes available. In 5 years, this CPG will either: (1) revised by APTA in accordance with new evidence, changing practice, rapidly emerging treatment options, and new technology; (2) reaffirmed; or (3) withdrawn.

**Low quality: 3-4**

Example: In conclusion, an up-to-date evidence-based PT guideline for the management of patients with hip or knee OA has been developed. To improve the quality of care for these patients, adequate dissemination, implementation, and timely updates are needed.

**Very low quality: 1**

Example: Did not specify updates.

**Domain 4. Clarity of Presentation**

15. **The recommendations are specific and unambiguous.**

Main location: Recommendation suggestions section

**High quality: 5-7**

Example: Physical therapists should implement resistance training to reduce motor disease severity and improve strength, power, nonmotor symptoms, functional outcomes, and quality of life in individuals with PD.

**Low quality: 3-4**

Example: Task practice with or without motor imaginary training for limb disability should be considered.

**Very low quality: 1-2**

Example: No recommendation suggestions formed.

16. **The different options for management of the condition or health issue are clearly presented.**

Main location: Discussion, recommendation suggestions section

**High quality: 5-7**

Example:

External figure 5. Illustrative example for AGREE II Item 16, showing high quality. Source: Zhang T, Zhao J, Li X, Bai Y, Wang B, Qu Y, et al. Chinese Stroke Association guidelines for clinical management of cerebrovascular disorders: executive summary and 2019 update of clinical management of stroke rehabilitation. Stroke Vasc Neurol. 2020 Sep;5(3):250-9. PMID: 32595138. doi: 10.1136/svn-2019-000321.

External figure 6. Illustrative example for AGREE II Item 16, showing high quality. Source: 中华医学会神经病学分会神经康复学组 中, 卫生部脑卒中筛查与防治工程委员会办公室. 中国脑卒中康复治疗指南(2011完全版). 中国医学前沿杂志. 2012 (6). doi: 10.3969/j.issn.1674-7372.2012.06.014.

**Low quality: 3-4**

Example: The treatment of atopic dermatitis requires an individual combination of therapy elements that are stage-appropriate and suited to the patient’s needs.

• external therapy

• systemic therapy

• phototherapy

• hydrotherapy/balneotherapy

• climate therapy

• diet

• relaxation techniques

• information/education

• sports therapy

• psychotherapy

**Very low quality: 1-2**

Example: Did not specify different options.

17. **Key recommendations are easily identifiable.**

Main location: Recommendation suggestions section and summary charts

**High quality: 5-7**

Example:

External figure 7. Illustrative example for AGREE II Item 17, showing high quality. Source: Osborne JA, Botkin R, Colon-Semenza C, DeAngelis TR, Gallardo OG, Kosakowski H, et al. Physical Therapist Management of Parkinson Disease: A Clinical Practice Guideline From the American Physical Therapy Association. Phys Ther. 2022 Apr 1;102(4). PMID: 34963139. doi: 10.1093/ptj/pzab302.

**Low quality: 3-4**

Example:

External figure 8. Illustrative example for AGREE II Item 17, showing low quality. Source: 中国康复医学会脊柱脊髓专业委员会基础研究学组 中. 急性脊柱脊髓损伤围术期管理临床指南. 中华创伤杂志. 2019;35(7):577-87. doi: 10.3760/cma.j.issn.1001-8050.2019.07.001.

**Very low quality: 1-2**

Example:

External figure 9. Illustrative example for AGREE II Item 17, showing very low quality. Source: 中国医师协会眼科医师分会眼视光专业委员会 中. 中国低视力康复临床指南. 中华眼视光学与视觉科学杂志. 2021;23(03):161-70. doi: 10.3760/cma.j.cn115909-20210221-00068.

**Domain 5. Applicability**

18. **The guideline describes facilitators and barriers to its application.**

Main location: Dissemination/implementation section

**High quality: 4-7**

Example: A potential barrier to implementation of this CPG is that physical therapist management of patients who have experienced a concussive event may require evaluation and treatment strategies that are typically provided by physical therapy specialists. Therefore, effective physical therapist management of concussion may necessitate referral to physical therapy specialists or other health care providers with necessary expertise appropriate across the continuum of management.

Additional barriers to implementation may include costs associated with training clinicians, lack of equipment, cultural barriers with local practice coordination or patterns that contrast with recommendations, and the additional time needed to examine, evaluate, and treat patients who have experienced a concussive event and have multiple impairments. Physical therapists are encouraged to use this CPG to support collaboration with other care providers managing patients with concussion in their local practice settings. The contents of this CPG may also be useful to inform discussions with clinic managers and administrators on how to set up infrastructure to ensure adequate time and resources, and to ensure that referral sources are dedicated to provide optimal care for patients who have experienced a concussive event.

Facilitators to implementing this CPG may include a local practice culture that embraces evidence-based practice and physical therapists who are trained to specifically manage patients who have experienced a concussive event. Another facilitator to implementation may be access to a multidisciplinary clinic or network of health care providers who can work together to help manage patients who have experienced a concussive event. Last, the complexity of concussive injuries may lead to highly variable care-delivery processes. Clinical pathways that support optimal patient referral and treatment flows that align with the recommendations proposed in this CPG are encouraged to facilitate direct integration into local practice settings.

**Low quality: 2-3**

Example: The 6MWT may have limited feasibility in certain settings, such as a hospital room or home environment with limited walkway space or fixed environmental barriers. Thus, clinicians will need to determine the feasibility and appropriateness of the 6MWT in specific situations.

**Very low quality: 1**

Example: No description of facilitators/barriers to application.

19. **The guideline provides advice and/or tools on how the recommendations can be put into practice.**

Main location: Dissemination/implementation section

**High quality: 5-7**

Example: The primary purpose of this CPG is to provide interested readers with full documentation of the best available evidence for various intervention strategies associated with the physical therapist management of PD. Publication of this CPG will be in PTJ: Physical Therapy and Rehabilitation Journal, the journal of the American Physical

Therapy Association. This CPG is available in Spanish; see Supplementary Appendix 1. Education and awareness about this CPG will be disseminated via online resources, such as webinars, podcasts, pocket guides https://www.guidelinecentral.com/aptamembers/, and continuing education courses; at professional annual meetings; and via social media. A CPG+, which includes an appraisal rating using the AGREE II tool, highlights, a check-your-practice section, and review comments, is available on APTA’s website (https://www.apta.org/patient-care/evidence-based-practice-resources/cpgs/parkinson-disease) for this CPG. A knowledge translation task force led by the APTA Academy of Neurologic Physical Therapy has been formed and will create additional implementation tools during the 3 years following the publication of this CPG. Please visit the CPG+ webpage or www.neuropt.org for details.

**Low quality: 2-4**

Example: Finally, implementation of the guideline in daily practice is important, and presentations and an E-learning module have been developed for this purpose. Information with regard to the guideline has been published on the KNGF website and in journals for professionals and patients.

**Very low quality: 1**

Example: Did not provide application tools.

20. **The potential resource implications of applying the recommendations have been considered.**

Main location: Methodology, dissemination/implementation section

**High quality: 4-7**

Example:

External figure 10. Illustrative example for AGREE II Item 20, showing high quality. Source: NICE. Stroke rehabilitation in adults. NICE. 2023.

**Low quality: 2-3**

Example: This CPG should not be construed as including all proper methods of care or excluding methods of care reasonably directed at obtaining the same results. The ultimate judgment regarding the application of any specific procedure or treatment must be made considering all circumstances presented by the individual, including safety, preferences, and disease stage, as well as the needs and resources particular to the locality or institution.

**Very low quality: 1**

Example: No information on potential relevant resources when applying recommendation suggestions.

21. **The guideline presents monitoring and/or auditing criteria.**

Main location: Discussion, recommendation suggestions section

**High quality: 5-7**

Example: Implementation and Audit:

The FGA is intended to assess balance while walking and has demonstrated a ceiling effect in individuals with balance and vestibular deficits seen in a tertiary care center. If a patient demonstrates a high score (near 30 out of 30), or is likely to do so, the clinician may need to select a more challenging OM to assess changes over time. If a patient is unable to ambulate, but has goals and capacity to improve balance, a baseline score of 0 should be documented on the FGA. For patients who perform well on the FGA and score near the top of the scale, it may not be necessary to readminister the test. Clinics and organizations should establish administration consistency within and among clinicians prior to using the FGA, and this should be repeated annually.

**Low quality: 2-4**

Example: CR exercise should be initiated as early as possible following acute phase treatment (SOR: strong/LOE: 1−).

**Very low quality: 1**

Example: No supervision/audit standards.

**Domain 6. Editorial Independence**

22. **The views of the funding body have not influenced the content of the guideline.**

Main location: The beginning section of the guideline, acknowledgments, statements

**High quality: 6-7**

Example: Throughout the CPG development process, the GDG received support through an APTA grant and sponsorship from the Academy of Orthopaedic Physical Therapy, the American Academy of Sports Physical Therapy, and the Academy of Neurologic Physical Therapy for training, travel, software, and librarian assistance. The funding bodies did not have any influence over the recommendations proposed.

**Low quality: 4-5**

Example: Development of this CPG was funded by the Royal Dutch Society for Physical Therapy (KNGF).

**Very low quality: 1-3**

Example: No information on sponsoring organization.

23. **Competing interests of guideline development group members have been recorded and addressed.**

Main location: The beginning section of the guideline, acknowledgments, statements, attachments

**High quality: 6-7**

Example: In accordance with APTA policy, all individuals whose names appear as authors or contributors to this CPG filed a disclosure statement as part of the submission process. All panel members provided full disclosure of potential conflicts of interest prior to voting on the recommendations contained within this CPG.

**Low quality: 4-5**

Example: The authors declare that there is no conflict of interest regarding the publication of this article or the development of the CPG.

**Very low quality: 1-3**

Example: No conflict of interest statement.

**Document 2. Code for Modified Intraclass Correlation Coefficient**

# Initialize various variables and functions
import pandas as pd
import numpy as np
from sklearn.metrics import cohen_kappa_score, confusion_matrix
from statsmodels.stats.inter_rater import cohens_kappa
from pingouin import intraclass_corr


# Define items within each domain
domains = {
    'domain1': ['item 1', 'item 2', 'item 3'],
    'domain2': ['item 4', 'item 5', 'item 6'],
    'domain3': ['item 7', 'item 8', 'item 9', 'item 10', 'item 11', 'item 12', 'item 13', 'item 14'],
    'domain4': ['item 15', 'item 16', 'item 17'],
    'domain5': ['item 18', 'item 19', 'item 20', 'item 21'],
    'domain6': ['item 22', 'item 23']
}

# Define score weight intervals within each domain (used only for ICC weighted calculation)
weights = {
    'domain1': ['3, 5', '3, 6', '3, 5'],
    'domain2': ['2, 6', '3, 5', '3, 5'],
    'domain3': ['3, 6', '2, 5', '3, 5', '3, 5', '2, 4', '2, 5', '3, 5', '3, 5'],
    'domain4': ['3, 5', '3, 5', '3, 5'],
    'domain5': ['2, 4', '2, 5', '2, 4', '2, 5'],
    'domain6': ['4, 6', '4, 6']
}

# Define the indices of items within each domain that share the same weight interval
domain_item_sameweight_index = {
    'domain1': [[0, 2], [1]],
    'domain2': [[0], [1, 2]],
    'domain3': [[0], [1, 5], [2, 3, 6, 7], [4]],
    'domain4': [[0, 1, 2]],
    'domain5': [[0, 2], [1, 3]],
    'domain6': [[0, 1]]
}

# Define mapping rules based on weight intervals to map original expert scores
# to scores reflected by the Xingrun rule (used only for ICC weighted calculation)
mapping_rules = {
    '3, 5': {1: 1, 2: 1.5, 3: 3.25, 4: 3.75, 5: 5.5, 6: 6, 7: 6.5},       
    '3, 6': {1: 1, 2: 1.5, 3: 3.5, 4: 4, 5: 4.5, 6: 6.5, 7: 7},           
    '2, 4': {1: 1, 2: 2.25, 3: 2.75, 4: 4.75, 5: 5.25, 6: 5.75, 7: 6.25}, 
    '2, 5': {1: 1, 2: 2.5, 3: 3, 4: 3.5, 5: 5.5, 6: 6, 7: 6.5},           
    '2, 6': {1: 1, 2: 2.75, 3: 3.25, 4: 3.75, 5: 4.25, 6: 6.5, 7: 7},     
    '4, 6': {1: 1.5, 2: 2, 3: 2.5, 4: 4.25, 5: 4.75, 6: 6.25, 7: 6.75}    
}

#  Utility function: return key based on value
def find_key_by_value(value, dictionary):
    """
    Return the corresponding key for a given value
    :param value: value to search for
    :param dictionary: dictionary containing key-value pairs
    :return: corresponding key
    """
    for key, values in dictionary.items():
        if value in values:
            return key
    return None

# Score mapping function: map original expert score based on weight interval
def map_score(score, rule):
    return mapping_rules[rule].get(score, score)

# Map original scores according to domain-specific weight intervals
def map_scores(expert_scores, domain_weights):
    mapped_scores = expert_scores.copy()
    idx = 0
    for domain, thresholds in domain_weights.items():
        for _, threshold in enumerate(thresholds):
            item_col = f'item {idx+1}'
            if item_col in expert_scores.columns:
                # print(item_col)
                # print(threshold)
                mapped_scores[item_col] = expert_scores[item_col].apply(lambda x: map_score(x, threshold))
            idx += 1
    return mapped_scores

# Define the first row of the weight confusion matrix based on weight intervals
# (used only for Cohen Kappa weighted calculation)
first_rows = {
    '3, 5': [0, 0.3, 0.6, 0.6, 0.9, 0.9, 0.9],
    '3, 6': [0, 0.3, 0.6, 0.6, 0.6, 0.9, 0.9],
    '2, 4': [0, 0.6, 0.6, 0.9, 0.9, 0.9, 0.9],
    '2, 5': [0, 0.6, 0.6, 0.6, 0.9, 0.9, 0.9],
    '2, 6': [0, 0.6, 0.6, 0.6, 0.6, 0.9, 0.9],
    '4, 6': [0, 0.3, 0.3, 0.6, 0.6, 0.9, 0.9]
}

# Categorize which items belong to the same weight interval
first_rows_index = {
    '3, 5': {1, 3, 5, 6, 9, 10, 13, 14, 15, 16, 17},
    '3, 6': {2, 7},
    '2, 4': {11, 18, 20},
    '2, 5': {8, 12, 19, 21},
    '2, 6': {4},
    '4, 6': {22, 23}
}

def determine_groups(first_row):
    """
    Determine grouping of rating categories
    :param first_row: first row of the weight confusion matrix
    :return: grouping list for 7-point Likert scale as described by Xingrun
             e.g., [[0], [1,2,3], [4,5,6]]
    """
    groups = []
    current_group = [0]
    first_weight = first_row[0]
    current_weight = first_row[1]
    for i in range(1, len(first_row)):
        if i == 1 and abs(first_row[i] - first_weight) == 0.6:
            groups.append(current_group)
            current_group = [i]
            current_weight = first_row[i]
        elif first_row[i] != current_weight:
            groups.append(current_group)
            current_group = [i]
            current_weight = first_row[i]
        else:
            current_group.append(i)
    groups.append(current_group)
    # print(f"Groups:{groups}")
    return groups

def generate_weight_confusion_matrix(first_row):
    """
     Generate a weighted confusion matrix
    :param first_row: first row of the confusion matrix
    :return: weighted confusion matrix
    """
    n = len(first_row)
    groups = determine_groups(first_row)
    matrix = np.zeros((n, n))
    for i in range(n):
        for j in range(n):
            if i == j:
                matrix[i, j] = 0
            else:
                group_i = next((g for g in groups if i in g), None)
                group_j = next((g for g in groups if j in g), None)
                
                if group_i == group_j:
                    matrix[i, j] = 0.3
                else:
                    group_i_index = groups.index(group_i)
                    group_j_index = groups.index(group_j)
                    if abs(group_i_index - group_j_index) == 1:
                        matrix[i, j] = 0.6
                    else:
                        matrix[i, j] = 0.9
    return matrix

# load data and compute overall default and weighted ICC
expert1 = pd.read_excel('724ai2.xlsx')
expert2 = pd.read_excel('724mxr1.xlsx')

# For weighted ICC, map the original scores
mapped_expert1 = map_scores(expert1, weights)
mapped_expert2 = map_scores(expert2, weights)

# Rename columns of expert1 and expert2, then concatenate
expert1.columns = [f'{col}_exp1' for col in expert1.columns]
expert1 = expert1.rename(columns={'id_exp1': 'id', 'title_exp1': 'title'}) 
expert2.columns = [f'{col}_exp2' for col in expert2.columns]
expert2 = expert2.drop(columns=['id_exp2', 'title_exp2'])

common_data_raw = pd.concat([expert1, expert2], axis=1)

# Rename columns of mapped_expert1 and mapped_expert2, then concatenate
mapped_expert1.columns = [f'{col}_exp1' for col in mapped_expert1.columns]
mapped_expert1 = mapped_expert1.rename(columns={'id_exp1': 'id', 'title_exp1': 'title'})
mapped_expert2.columns = [f'{col}_exp2' for col in mapped_expert2.columns]
mapped_expert2 = mapped_expert2.drop(columns=['id_exp2', 'title_exp2'])

common_data_weightd = pd.concat([mapped_expert1, mapped_expert2], axis=1)

# Extract raw and weighted ICC-mapped scores for all items
items_e1_raw = common_data_raw.filter(regex='item.*_exp1').values.flatten()
items_e2_raw = common_data_raw.filter(regex='item.*_exp2').values.flatten()
items_e1_weighted = common_data_weightd.filter(regex='item.*_exp1').values.flatten()
items_e2_weighted = common_data_weightd.filter(regex='item.*_exp2').values.flatten()


# Prepare data format for ICC calculation (same format for default and weighted ICC)
icc_data_raw = pd.DataFrame({
    'expert': ['exp1'] * len(items_e1_raw) + ['exp2'] * len(items_e2_raw),
    'raw_score': list(items_e1_raw) + list(items_e2_raw),
    'id': list(range(len(items_e1_raw))) + list(range(len(items_e2_raw)))
})
icc_data_weighted = pd.DataFrame({
    'expert': ['exp1'] * len(items_e1_weighted) + ['exp2'] * len(items_e2_weighted),
    'score': list(items_e1_weighted) + list(items_e2_weighted),
    'id': list(range(len(items_e1_weighted))) + list(range(len(items_e2_weighted)))
})

# Compute overall ICC
overall_icc_raw = intraclass_corr(data=icc_data_raw, targets='id', raters='expert', ratings='raw_score')
print("1. Overall default ICC:\n", overall_icc_raw)
print("\n")

overall_icc = intraclass_corr(data=icc_data_weighted, targets='id', raters='expert', ratings='score')
print("2. Overall weighted ICC:\n", overall_icc)
print("\n")

# Initialize result dictionary
base_confusion_matrix = confusion_matrix(items_e1_raw, items_e2_raw, labels=range(1, 8), sample_weight=None, normalize=None)
overall_ck_raw = cohens_kappa(base_confusion_matrix, return_results=False)

print("3. Overall default Cohen's Kappa:\n", overall_ck_raw)
print("\n")

# Initialize result dictionary
results = {'cohen_kappa': {'overall_default': overall_ck_raw, 'overall_weighted': 0}, 'icc': {'overall_default': overall_icc_raw, 'overall_weighted': overall_icc}, 'default_icc':{}, 'weighted_icc':{}}

for domain, items in domains.items():
    
    domain_kappa_weighted = []
    item_list = domains[domain]
    weight_range_list = weights[domain]
    weight_indexes_lists = domain_item_sameweight_index[domain]
    for weight_indexs in weight_indexes_lists:
        item_e1 = []
        item_e2 = []
        weight_range_index = 0
        for index in weight_indexs:
            item = item_list[index]
            item_e1.append(common_data_raw[f'{item}_exp1'].values.flatten())
            item_e2.append(common_data_raw[f'{item}_exp2'].values.flatten())
            weight_range_index = index
        item_e1 = np.concatenate(item_e1)
        item_e2 = np.concatenate(item_e2)
        weight_range = weight_range_list[weight_range_index]
        base_confusion_matrix = confusion_matrix(item_e1, item_e2, labels=range(1, 8), sample_weight=None, normalize=None)
        weight_matrix = generate_weight_confusion_matrix(first_rows[weight_range])
        domain_kappa_weighted.append(cohens_kappa(base_confusion_matrix, weight_matrix, return_results=False))
    domain_kappa_mean_weighted = np.mean(domain_kappa_weighted)
    domain_kappa_std_weighted = np.std(domain_kappa_weighted)
    
    # Extract corresponding item data from common_data_raw and store column by column in a list
    items_e1_raw = [common_data_raw[f"{item}_exp1"].values for item in items]
    items_e2_raw = [common_data_raw[f"{item}_exp2"].values for item in items]
    items_e1_weighted = [common_data_weightd[f"{item}_exp1"].values for item in items]
    items_e2_weighted = [common_data_weightd[f"{item}_exp2"].values for item in items]

    items_e1_raw = np.concatenate(items_e1_raw)
    items_e2_raw = np.concatenate(items_e2_raw)
    items_e1_weighted = np.concatenate(items_e1_weighted)
    items_e2_weighted = np.concatenate(items_e2_weighted)

    base_confusion_matrix = confusion_matrix(items_e1_raw, items_e2_raw, labels=range(1, 8), sample_weight=None, normalize=None)
    domain_kappa_raw = cohens_kappa(base_confusion_matrix, return_results=False)
 
    domain_cohen_kappa = {
    'Type': ['Default', 'Weighted'],
    'CohenKappa': [domain_kappa_raw, domain_kappa_mean_weighted]
              }
    domain_cohen_kappa = pd.DataFrame(domain_cohen_kappa)
    results['cohen_kappa'][domain] = domain_cohen_kappa

    # Prepare data format for computing default ICC
    icc_data_raw = pd.DataFrame({
        'expert': ['e1'] * len(items_e1_raw) + ['e2'] * len(items_e2_raw),
        'score': list(items_e1_raw) + list(items_e2_raw),
        'id': list(range(len(items_e1_raw))) + list(range(len(items_e2_raw)))
    })
    # Compute domain-level ICC
    domain_icc_raw = intraclass_corr(data=icc_data_raw, targets='id', raters='expert', ratings='score')
    results['default_icc'][domain] = domain_icc_raw

    #  Prepare data format for computing weighted ICC
    icc_data_weighted = pd.DataFrame({
        'expert': ['e1'] * len(items_e1_weighted) + ['e2'] * len(items_e2_weighted),
        'score': list(items_e1_weighted) + list(items_e2_weighted),
        'id': list(range(len(items_e1_weighted))) + list(range(len(items_e2_weighted)))
    })
    # Compute domain-level weighted ICC
    domain_icc_weighted = intraclass_corr(data=icc_data_weighted, targets='id', raters='expert', ratings='score')
    results['weighted_icc'][domain] = domain_icc_weighted

# Output results
for domain in domains.keys():
    print(f'{domain} Cohen Kappa:', results['cohen_kappa'][domain])
    print("\n")
    print(f'{domain} default ICC:', results['default icc'][domain])
    print("\n")
    print(f'{domain} weighted ICC:', results['weighted icc'][domain])
    print("\n")

**Screening result**

**Table S3. Full-text records assessed, reviewed, and excluded from the analysis (n=80) with reasons for exclusion by reference**

**No accessible full text (n = 3)**

**Not a CPG(n =46)**

**Not on rehabilitation (n = 19)**

**Other languages(n = 11)**

**Translation (n= 1)**

| **ID** | **Year** | **Title** | **Reason for Exclusion** |
| --- | --- | --- | --- |
| 1 | 2022 | Occupational Therapy Practice Guidelines for Adults With Chronic Conditions | No accessible full text |
| 2 | 2020 | Occupational Therapy Practice Guidelines for Older Adults With Low Vision | No accessible full text |
| 3 | 2020 | Occupational Therapy Practice Guidelines for Early Childhood: Birth-5 Years | No accessible full text |
| 4 | 2011 | 儿童孤独症诊疗康复指南(卫办医政发(2010]123号) | Not a CPG |
| 5 | 2022 | At-Home Exercise Guide for People With Chronic Kidney Disease | Not a CPG |
| 6 | 2016 | Guidelines and treatment pathways of neurological rehabilitation | Not a CPG |
| 7 | 2005 | Canadian guidelines for cardiac rehabilitation and cardiovascular disease prevention, second edition, 2004: executive summary | Not a CPG |
| 8 | 2001 | Exercise prescription for older adults with osteoarthritis pain: consensus practice recommendations. A supplement to the AGS Clinical Practice Guidelines on the management of chronic pain in older adults | Not a CPG |
| 9 | 1995 | Post-stroke rehabilitation: assessment, referral, and patient management. U.S. Department of Health and Human Services Public Health Service. Agency for Health Care Policy and Research | Not a CPG |
| 10 | 2023 | 新型冠状病毒感染后全程中西医心肺康复专家共识 | Not a CPG |
| 11 | 2023 | 老年患者新型冠状病毒感染诊疗与康复专家共识 | Not a CPG |
| 12 | 2023 | 老年帕金森病功能障碍全周期康复专家共识 | Not a CPG |
| 13 | 2023 | 口腔颌面头颈肿瘤术后康复护理专家共识 | Not a CPG |
| 14 | 2023 | 以功能障碍为中心的中国癌症患者运动康复专家共识 | Not a CPG |
| 15 | 2022 | 先天性并指畸形康复专家共识 | Not a CPG |
| 16 | 2022 | 经皮冠状动脉介入术后中西医结合心脏康复专家共识 | Not a CPG |
| 17 | 2022 | 中国心血管疾病患者居家康复专家共识 | Not a CPG |
| 18 | 2020 | 运动疗法治疗颈痛的中国专家共识 | Not a CPG |
| 19 | 2020 | 下肢关节周围骨质疏松性骨折诊疗与康复专家共识 | Not a CPG |
| 20 | 2020 | 基于世界卫生组织国际健康分类家族架构的2019冠状病毒病康复方案专家共识 | Not a CPG |
| 21 | 2020 | 冠状动脉旁路移植术后心脏康复专家共识 | Not a CPG |
| 22 | 2020 | 经导管主动脉瓣置换术后运动康复专家共识 | Not a CPG |
| 23 | 2019 | 中国慢性踝关节外侧不稳定术后康复专家共识 | Not a CPG |
| 24 | 2019 | 中国老年膝关节骨关节炎诊疗及智能矫形康复专家共识 | Not a CPG |
| 25 | 2019 | 中国高龄脑卒中患者康复治疗技术专家共识 | Not a CPG |
| 26 | 2018 | 脑卒中合并稳定性冠心病运动康复专家共识 | Not a CPG |
| 27 | 2017 | 浙江省重症康复专家共识 | Not a CPG |
| 28 | 2017 | 中西医结合冠状动脉旁路移植术Ⅰ期心脏康复专家共识 | Not a CPG |
| 29 | 2016 | 中国脑梗死急性期康复专家共识 | Not a CPG |
| 30 | 2016 | 经皮冠状动脉介入治疗术后运动康复专家共识 | Not a CPG |
| 31 | 2008 | 《骨关节炎的康复治疗》专家共识 | Not a CPG |
| 32 | 2022 | Exercise/Physical Activity in Individuals with Type 2 Diabetes: A Consensus Statement from the American College of Sports Medicine | Not a CPG |
| 33 | 2021 | International Exercise Recommendations in Older Adults (ICFSR): Expert Consensus Guidelines | Not a CPG |
| 34 | 2020 | An Italian consensus on pulmonary rehabilitation in COVID-19 patients recovering from acute respiratory failure: Results of a Delphi process | Not a CPG |
| 35 | 2020 | Cardiac Rehabilitation During the COVID-19 Era: Guidance on Implementing Virtual Care | Not a CPG |
| 36 | 2020 | Enhanced recovery after surgery for degenerative scoliosis: Consensus on perioperative management strategy | Not a CPG |
| 37 | 2019 | Clinical practice guidelines for the management of brain tumours: A rehabilitation perspective | Not a CPG |
| 38 | 2019 | Chinese Expert Consensus on Enhanced Recovery After Hepatectomy (Version 2017) | Not a CPG |
| 39 | 2018 | Exercise for Knee Injury Prevention: A Summary of Clinical Practice Guideline Recommendations-Using the Evidence to Guide Physical Therapist Practice | Not a CPG |
| 40 | 2017 | Improving the development, monitoring and reporting of stroke rehabilitation research: Consensus-based core recommendations from the Stroke Recovery and Rehabilitation Roundtable | Not a CPG |
| 41 | 2015 | Physiotherapy for functional motor disorders: A consensus recommendation | Not a CPG |
| 42 | 2011 | American College of Sports Medicine position stand. Quantity and quality of exercise for developing and maintaining cardiorespiratory, musculoskeletal, and neuromotor fitness in apparently healthy adults: guidance for prescribing exercise | Not a CPG |
| 43 | 2009 | Guidelines for the physiotherapy management of the adult, medical, spontaneously breathing patient | Not a CPG |
| 44 | 2012 | Rehabilitation after arthroscopic rotator cuff repair: current concepts review and evidence-based guidelines | Not a CPG |
| 45 | 2008 | Adherence to, and results of, physical therapy programs in patients with hip or knee osteoarthritis. Development of French clinical practice guidelines | Not a CPG |
| 46 | 2012 | Rehabilitation after arthroscopic rotator cuff repair: current concepts review and evidence-based guidelines | Not a CPG |
| 47 | 2008 | Adherence to, and results of, physical therapy programs in patients with hip or knee osteoarthritis. Development of French Clinical Practice Guidelines | Not a CPG |
| 48 | 2016 | Restorative dentistry and oral rehabilitation: United Kingdom National Multidisciplinary Guidelines | Not a CPG |
| 49 | 2026 | Stroke rehabilitation: NICE guideline update | Not a CPG |
| 50 | 2018 | 骨质疏松性骨折围手术期干预指南 | Not on rehabilitation |
| 51 | 2015 | 肌肉减少症营养治疗指南 | Not on rehabilitation |
| 52 | 2014 | 人工耳蜗植入工作指南 | Not on rehabilitation |
| 53 | 2010 | 肉毒毒素治疗成人肢体肌痉挛中国指南2010 | Not on rehabilitation |
| 54 | 2019 | 中国脑血管病临床管理指南(节选版)——卒中组织化管理 | Not on rehabilitation |
| 55 | 2021 | Guide to Enhanced Recovery for Cancer Patients Undergoing Surgery: Pancreaticoduodenectomy | Not on rehabilitation |
| 56 | 2020 | The Belgian national guideline on low back pain and radicular pain: key roles for rehabilitation, assessment of rehabilitation potential and the PRM specialist | Not on rehabilitation |
| 57 | 2019 | Guidelines for Perioperative Care in Esophagectomy: Enhanced Recovery After Surgery (ERAS®) Society Recommendations | Not on rehabilitation |
| 58 | 2019 | Rehabilitation before regenerative cartilage knee surgery: a new prehabilitation guideline based on the best available evidence | Not on rehabilitation |
| 59 | 2019 | Clinical guidelines on perioperative management strategies for enhanced recovery after lung surgery | Not on rehabilitation |
| 60 | 2019 | Guidelines for enhanced recovery after lung surgery: Recommendations of the Enhanced Recovery after Surgery (ERAS®) Society and the European Society of Thoracic Surgeons (ESTS) | Not on rehabilitation |
| 61 | 2018 | Practice guideline update recommendations summary: Disorders of consciousness: Report of the Guideline Development, Dissemination, and Implementation Subcommittee of the American Academy of Neurology; the American Congress of Rehabilitation Medicine; and the National Institute on Disability, Independent Living, and Rehabilitation Research | Not on rehabilitation |
| 62 | 2017 | Development of practice guidelines for psychological interventions in the rehabilitation of patients with oncological disease (breast, prostate, or colorectal cancer): Methods and results | Not on rehabilitation |
| 63 | 2017 | Clinical Practice Guidelines for Enhanced Recovery After Colon and Rectal Surgery From the American Society of Colon and Rectal Surgeons and Society of American Gastrointestinal and Endoscopic Surgeons | Not on rehabilitation |
| 64 | 2016 | Restorative dentistry and oral rehabilitation: United Kingdom National Multidisciplinary Guidelines | Not on rehabilitation |
| 65 | 2011 | Ottawa Panel evidence-based clinical practice guidelines for the management of osteoarthritis in adults who are obese or overweight | Not on rehabilitation |
| 66 | 1996 | AACE Clinical Practice Guidelines for the Prevention and Treatment of Postmenopausal Osteoporosis | Not on rehabilitation |
| 67 | 2026 | American College of Surgeons Best Practice Guidelines on Management of Traumatic Brain Injury: Executive Summary of Early Rehabilitation Recommendations | Not on rehabilitation |
| 68 | 2026 | Chinese Clinical Practice Guidelines for Auditory Neuropathy (gCAN) | Not on rehabilitation |
| 69 | 2020 | Brazilian cardiovascular rehabilitation guideline: Values and limitations | Other languages |
| 70 | 2020 | Brazilian cardiovascular rehabilitation guideline – 2020 | Other languages |
| 71 | 2019 | Clinical Practice Guidelines for the prevention, diagnosis, treatment, and rehabilitation of heart failure in a population greater than 18 years-old: B, C and D Classification. Cardiac rehabilitation component | Other languages |
| 72 | 2019 | Rehabilitation of Patients with Peripheral Arterial Disease in the Context of Guideline Recommendations | Other languages |
| 73 | 2018 | Cardiovascular rehabilitation and prevention guideline update | Other languages |
| 74 | 2018 | Guidelines for cardiovascular rehabilitation and secondary prevention in China 2018 simplified edition | Other languages |
| 75 | 2017 | Prolonged weaning during early neurological and neurosurgical rehabilitation: S2k guideline published by the Weaning Committee of the German Neurorehabilitation Society (DGNR) | Other languages |
| 76 | 2017 | Guide to clinical practice for the diagnosis, treatment and rehabilitation of non-syndromic craniosynostosis on 3 levels of care | Other languages |
| 77 | 2017 | Guidelines for early rehabilitation of stroke in China | Other languages |
| 78 | 2016 | Surgical and rehabilitation treatment for patients with Muscular Dystrophies. Evidence-based recommendations for clinical practice guidelines in Colombia | Other languages |
| 79 | 1999 | ANMCO-SIC-GIVFRC guidelines on cardiological rehabilitation | Other languages |
| 80 | 2021 | 5～21岁儿童和青少年作业治疗实践指南 | Translation |

**Quality assessment**

**Table S4. Interobserver agreement using intraclass correlation coefficient for quality appraisal of CPGs using AGREEⅡ instrument stratified by domain.**

|  | Intraclass correlation coefficient (Mean 95%CI) | Degree of agreement |
| --- | --- | --- |
| Domain 1 Scope and Purpose | 0.861998 [0.83, 0.89] | good |
| Domain 2 Stakeholder Involvement | 0.916437 [0.90, 0.93] | excellent |
| Domain 3 Rigor of Development | 0.884843 [0.87, 0.90] | good |
| Domain 4 Clarity of Presentation | 0.918328 [0.90, 0.93] | excellent |
| Domain 5 Applicability | 0.840211 [0.81, 0.86] | good |
| Domain 6 Editorial Independence | 0.896743 [0.87, 0.92] | good |

ICC <0.5 indicates poor reliability; 0.5-0.75, moderate reliability; 0.75-0.9, good reliability; and >0.9, excellent reliability.(Altman DG. Practical Statistics For Medical Research. London: Chapman and Hall, 1991)

**Table S5. Interobserver agreement using intraclass correlation coefficient for reporting appraisal of CPGs using RIGHT checklist stratified by domain.**

|  | Intraclass correlation coefficient (Mean 95%CI) | Degree of agreement |
| --- | --- | --- |
| Domain 1 Basic information | 0.849 [0.83, 0.86] | good |
| Domain 2 Background | 0.882 [0.87, 0.89] | good |
| Domain 3 Evidence | 0.802 [0.78, 0.82] | good |
| Domain 4 Recommendations | 0.829 [0.81, 0.85] | good |
| Domain 5 Review and quality assurance | 0.818 [0.78, 0.85] | good |
| Domain 6 Funding and declaration and management of interests | 0.871 [0.85, 0.89] | good |
| Domain 7 Other information | 0.845 [0.82, 0.87] | good |

ICC <0.5 indicates poor reliability; 0.5-0.75, moderate reliability; 0.75-0.9, good reliability; and >0.9, excellent reliability.(Altman DG. Practical Statistics For Medical Research. London: Chapman and Hall, 1991)

**Table S6. Intraclass correlation coefficient of five rounds of appraisal using AGREE II instrument.**

|  | Domain 1 Scope and Purpose | Domain 2 Stakeholder Involvement | Domain 3 Rigor of Development | Domain 4 Clarity of Presentation | Domain 5 Applicability | Domain 6 Editorial Independence |
| --- | --- | --- | --- | --- | --- | --- |
| First appraisal | 0.22 | 0.45 | 0.66 | -0.09 | 0.21 | 0.65 |
| Second appraisal | 0.66 | 0.74 | 0.79 | 0.43 | 0.57 | 0.81 |
| Third appraisal | 0.87 | 0.92 | 0.89 | 0.93 | 0.84 | 0.90 |
| Fourth appraisal | 0.86 | 0.91 | 0.86 | 0.89 | 0.82 | 0.87 |
| Fifth appraisal | 0.86 | 0.92 | 0.88 | 0.92 | 0.84 | 0.90 |

**Table S7. Scores of 227 CPGs using AGREE Ⅱ Instrument**

| ID | Year | Title | D1 | D2 | D3 | D4 | D5 | D6 |
| --- | --- | --- | --- | --- | --- | --- | --- | --- |
| 1 | 2026 | 创伤性脊髓损伤康复指南与规范（2025年版） | 83 | 39 | 6 | 92 | 4 | 83 |
| 2 | 2026 | 中国慢性呼吸疾病呼吸康复指南（2026版） | 97 | 83 | 96 | 94 | 83 | 58 |
| 3 | 2025 | 儿童发育性协调障碍的早期干预和康复指南(2025) | 92 | 50 | 74 | 92 | 4 | 54 |
| 4 | 2025 | 儿童柔性扁平足康复管理指南(2025) | 94 | 47 | 75 | 94 | 23 | 54 |
| 5 | 2025 | 儿童重症早期康复介入的临床实践指南（2025） | 94 | 53 | 60 | 97 | 4 | 33 |
| 6 | 2025 | 慢性气道疾病康复基层指南（2025年） | 97 | 47 | 23 | 97 | 4 | 50 |
| 7 | 2024 | 脑卒中中西医结合康复临床循证实践指南 | 83 | 53 | 66 | 83 | 42 | 75 |
| 8 | 2024 | 脊髓损伤中西医结合康复临床实践指南 | 64 | 50 | 50 | 67 | 46 | 29 |
| 9 | 2024 | 中国2型糖尿病运动治疗指南（2024版） | 58 | 58 | 59 | 69 | 25 | 54 |
| 10 | 2024 | 中国冠心病康复循证实践指南(2024版)第一部分 | 50 | 25 | 31 | 86 | 15 | 50 |
| 11 | 2024 | 中国膝骨关节炎康复治疗指南（2023版） | 78 | 61 | 78 | 83 | 38 | 79 |
| 12 | 2023 | 中医康复临床实践指南·儿童青少年特发性脊柱侧弯 | 64 | 50 | 51 | 72 | 8 | 83 |
| 13 | 2023 | 中医康复临床实践指南·悬吊推拿运动技术 | 75 | 56 | 55 | 75 | 21 | 79 |
| 14 | 2023 | 新型冠状病毒感染恢复期常见症中西医结合管理循证实践指南 | 92 | 69 | 54 | 86 | 38 | 83 |
| 15 | 2023 | 中轴脊柱关节炎/强直性脊柱炎康复实践指南(2023版) | 72 | 64 | 49 | 72 | 27 | 67 |
| 16 | 2023 | 特发性肺纤维化中医康复指南(2021-10-21) | 50 | 58 | 41 | 67 | 13 | 67 |
| 17 | 2023 | 膝骨关节炎针刀临床诊疗指南 | 61 | 56 | 10 | 75 | 10 | 25 |
| 18 | 2023 | 中国骨肌疾病体外冲击波疗法指南(2023年版) | 50 | 58 | 53 | 56 | 13 | 25 |
| 19 | 2023 | 中国吞咽障碍康复管理指南(2023版) | 83 | 69 | 60 | 75 | 13 | 71 |
| 20 | 2022 | 中国纤维肌痛康复指南(2021) | 78 | 28 | 52 | 94 | 17 | 50 |
| 21 | 2022 | 改良太极功法临床实践指南·脑卒中运动障碍 | 64 | 44 | 39 | 92 | 10 | 17 |
| 22 | 2022 | 中国膝骨关节炎基层诊疗与康复指南(2022版) | 78 | 64 | 54 | 94 | 46 | 67 |
| 23 | 2022 | 中国非特异性腰背痛临床诊疗指南 | 78 | 58 | 59 | 72 | 23 | 46 |
| 24 | 2022 | 脊髓损伤康复治疗临床实践指南 | 61 | 64 | 48 | 56 | 23 | 63 |
| 25 | 2022 | 中国老年人肌少症临床康复治疗指南 | 50 | 42 | 10 | 69 | 15 | 71 |
| 26 | 2022 | 周围神经损伤居家康复指南 | 81 | 56 | 65 | 81 | 19 | 0 |
| 27 | 2022 | 中国脑性瘫痪康复指南(2022) | 50 | 58 | 50 | 61 | 29 | 67 |
| 28 | 2022 | 腰椎间盘突出症诊治与康复管理指南 | 61 | 31 | 42 | 58 | 17 | 33 |
| 29 | 2022 | 骨质疏松性椎体压缩骨折椎体强化术后康复治疗指南（2022版） | 58 | 33 | 52 | 53 | 29 | 50 |
| 30 | 2021 | 冠心病心脏康复基层合理用药指南 | 83 | 53 | 45 | 72 | 58 | 33 |
| 31 | 2021 | 冠心病心脏康复基层指南（2020年） | 75 | 56 | 35 | 67 | 29 | 42 |
| 32 | 2021 | 蒙医康复临床实践指南·脑梗死 | 83 | 53 | 54 | 72 | 63 | 33 |
| 33 | 2021 | 中医康复临床实践指南·不完全性截瘫 | 83 | 56 | 58 | 78 | 58 | 46 |
| 34 | 2021 | 中医康复临床实践指南·缺血性脑卒中(脑梗死) | 78 | 61 | 52 | 72 | 50 | 50 |
| 35 | 2021 | 中医康复临床实践指南·腰痛(腰椎间盘突出症) | 78 | 56 | 44 | 72 | 33 | 0 |
| 36 | 2021 | 尘肺病康复专家共识（2021版） | 78 | 58 | 53 | 61 | 56 | 71 |
| 37 | 2021 | 慢性阻塞性肺疾病临床康复循证实践指南 | 69 | 92 | 59 | 75 | 35 | 83 |
| 38 | 2021 | 慢性阻塞性肺疾病中医肺康复临床应用指南 | 56 | 56 | 55 | 67 | 23 | 79 |
| 39 | 2021 | 脑性瘫痪儿童水中运动治疗临床实践指南 | 81 | 67 | 63 | 72 | 27 | 67 |
| 40 | 2021 | 中国慢性呼吸道疾病呼吸康复管理指南（2021年） | 69 | 61 | 57 | 69 | 35 | 63 |
| 41 | 2021 | 中国低视力康复临床指南（2021） | 50 | 44 | 19 | 61 | 19 | 50 |
| 42 | 2020 | 中医康复临床实践指南·类风湿关节炎 | 72 | 50 | 42 | 72 | 38 | 17 |
| 43 | 2020 | 中医康复临床实践指南·膝骨关节炎 | 67 | 50 | 44 | 72 | 38 | 17 |
| 44 | 2020 | 中医康复临床实践指南·项痹(颈椎病) | 67 | 61 | 54 | 72 | 46 | 25 |
| 45 | 2020 | 中医康复临床实践指南·心肺康复 | 61 | 56 | 48 | 72 | 46 | 17 |
| 46 | 2020 | 新型冠状病毒肺炎恢复期中西医结合康复指南（第一版） | 75 | 53 | 13 | 75 | 19 | 8 |
| 47 | 2020 | 新型冠状病毒肺炎中医康复专家共识(第一版) | 58 | 61 | 48 | 78 | 29 | 67 |
| 48 | 2020 | 中西医结合康复临床实践指南·认知障碍 | 58 | 33 | 19 | 69 | 13 | 21 |
| 49 | 2020 | 危重症新型冠状病毒肺炎患者后ICU综合征呼吸康复推荐意见 | 67 | 42 | 41 | 69 | 23 | 58 |
| 50 | 2019 | 中国骨肌疾病体外冲击波疗法指南(2019年版) | 75 | 42 | 36 | 69 | 38 | 13 |
| 51 | 2019 | 脑卒中水中运动治疗中国循证临床实践指南(2019版) | 64 | 50 | 55 | 72 | 35 | 54 |
| 52 | 2019 | 中医康复临床实践指南·脑卒中 | 58 | 42 | 34 | 50 | 31 | 8 |
| 53 | 2019 | 骨质疏松症康复指南（上） | 81 | 94 | 75 | 89 | 58 | 71 |
| 54 | 2019 | 物理技术辅助脑卒中康复的临床指南 | 56 | 28 | 8 | 64 | 17 | 29 |
| 55 | 2019 | 急性脊柱脊髓损伤围术期管理临床指南 | 64 | 28 | 53 | 72 | 25 | 17 |
| 56 | 2017 | 中医骨伤科临床诊疗指南·人工髋关节置换围手术期康复专家共识 | 58 | 53 | 47 | 67 | 27 | 83 |
| 57 | 2017 | 中国脑卒中早期康复治疗指南 | 58 | 33 | 14 | 72 | 23 | 0 |
| 58 | 2015 | 中国脑性瘫痪康复指南(2015) | 56 | 42 | 48 | 56 | 40 | 29 |
| 59 | 2013 | 脊髓损伤患者泌尿系管理与临床康复指南 | 61 | 36 | 16 | 75 | 17 | 25 |
| 60 | 2013 | 烧伤康复治疗指南(2013版) | 61 | 22 | 10 | 64 | 10 | 0 |
| 61 | 2012 | 中国脑卒中康复治疗指南(2011完全版) | 61 | 28 | 26 | 61 | 10 | 25 |
| 62 | 2012 | 中国卒中康复治疗指南简化版 | 58 | 11 | 19 | 69 | 8 | 0 |
| 63 | 2008 | 地震伤员康复治疗指南 | 64 | 33 | 3 | 72 | 23 | 0 |
| 64 | 1993 | 肝病患者康复指南 | 28 | 14 | 4 | 28 | 6 | 0 |
| 65 | 2026 | ASSOBRAFIR clinical practice guidelines in respiratory physical therapy: Exercise-based interventions in people with chronic obstructive pulmonary disease (COPD) | 92 | 69 | 78 | 64 | 67 | 67 |
| 66 | 2026 | Canadian Stroke Best Practice Recommendations Rehabilitation, Recovery, and Community Participation Following Stroke, Part Three: Optimizing Activity and Community Participation Following Stroke , 7th Edition Update, 2025 | 100 | 89 | 86 | 94 | 77 | 83 |
| 67 | 2026 | Canadian Stroke Best Practice Recommendations Rehabilitation, Recovery, and Community Participation Following Stroke, Part Two: Delivery of Stroke Rehabilitation to Optimize Functional Recovery, 7th Edition Update 2025 | 97 | 86 | 86 | 97 | 85 | 83 |
| 68 | 2026 | Canadian Stroke Best Practice Recommendations: Rehabilitation, Recovery and Community Participation Following Stroke. Part One: Stroke Rehabilitation Planning for Optimal Care Delivery, 7th Edition Update 2025 | 100 | 89 | 86 | 97 | 83 | 83 |
| 69 | 2026 | Clinical guideline on the open arthrolysis for post-traumatic elbow stiffness in adult patients | 97 | 78 | 96 | 97 | 63 | 100 |
| 70 | 2025 | ASSOBRAFIR clinical practice guidelines in cardiovascular physical therapy: Exercise-based interventions in outpatient rehabilitation programs for heart failure | 92 | 69 | 81 | 67 | 71 | 67 |
| 71 | 2025 | Australian clinical practice guideline for physical rehabilitation and mobilisation in adult intensive care units | 97 | 83 | 83 | 100 | 83 | 83 |
| 72 | 2025 | Chinese Guideline for the Rehabilitation Treatment of Osteoporosis (2024 Edition) | 97 | 78 | 88 | 97 | 27 | 75 |
| 73 | 2025 | Clinical practice guidelines for rehabilitation following surgical management of chronic lateral ankle instability: enhancing recovery based on available evidence | 97 | 28 | 65 | 86 | 29 | 42 |
| 74 | 2025 | European Stroke Organisation (ESO) guideline on aphasia rehabilitation | 97 | 72 | 90 | 89 | 15 | 67 |
| 75 | 2025 | European Stroke Organisation (ESO) guideline on motor rehabilitation | 72 | 61 | 76 | 81 | 29 | 42 |
| 76 | 2025 | Guidelines for Rehabilitation Interventions in Older Adults With Sarcopenia (2025 Edition) | 100 | 61 | 94 | 97 | 52 | 58 |
| 77 | 2025 | National Institute for Health and Care Excellence: Clinical Guidelines | 100 | 67 | 20 | 39 | 48 | 8 |
| 78 | 2025 | Rehabilitation of the lower extremities, standing and walking function in people with spinal cord injury or disease: Guideline of the German-Speaking Medical Society for Spinal Cord Injury | 100 | 75 | 75 | 67 | 27 | 50 |
| 79 | 2025 | Rotator Cuff Tendinopathy Diagnosis, Nonsurgical Medical Care, and Rehabilitation: A Clinical Practice Guideline | 100 | 67 | 70 | 97 | 52 | 50 |
| 80 | 2024 | Development of clinical practice guidelines for rehabilitation after diagnosis for primary bone and soft tissue tumours | 97 | 33 | 28 | 31 | 33 | 42 |
| 81 | 2024 | Telerehabilitation in Physical Therapist Practice: A Clinical Practice Guideline From the American Physical Therapy Association | 58 | 56 | 70 | 75 | 35 | 54 |
| 82 | 2024 | Digital technologies to deliver pulmonary rehabilitation programmes for adults with COPD: early value assessment | 56 | 50 | 47 | 75 | 58 | 46 |
| 83 | 2024 | GaitSmart rehabilitation exercise programme for gait and mobility issues | 61 | 39 | 34 | 75 | 27 | 42 |
| 84 | 2024 | VA/DoD CLINICAL PRACTICE GUIDELINE FOR MANAGEMENT OF STROKE REHABILITATION | 78 | 47 | 47 | 67 | 52 | 17 |
| 85 | 2023 | Physical Therapist Management of Glenohumeral Joint Osteoarthritis: A Clinical Practice Guideline from the American Physical Therapy Association | 81 | 69 | 84 | 67 | 73 | 88 |
| 86 | 2023 | Stroke rehabilitation in adults | 83 | 78 | 82 | 78 | 65 | 42 |
| 87 | 2023 | Acupuncture for treatment of knee osteoarthritis: A clinical practice guideline | 72 | 64 | 42 | 50 | 44 | 83 |
| 88 | 2023 | 2022 American College of Rheumatology Guideline for Exercise, Rehabilitation, Diet, and Additional Integrative Interventions for Rheumatoid Arthritis | 64 | 53 | 50 | 64 | 40 | 17 |
| 89 | 2022 | Physical Therapist Management of Parkinson Disease: A Clinical Practice Guideline From the American Physical Therapy Association | 83 | 64 | 76 | 58 | 67 | 83 |
| 90 | 2022 | Brazilian practice guidelines for stroke rehabilitation | 64 | 47 | 25 | 67 | 50 | 29 |
| 91 | 2022 | Women-Focused Cardiovascular Rehabilitation: An International Council of Cardiovascular Prevention and Rehabilitation Clinical Practice Guideline | 72 | 78 | 71 | 89 | 58 | 75 |
| 92 | 2022 | Rehabilitation after traumatic injury | 89 | 78 | 82 | 72 | 63 | 54 |
| 93 | 2022 | Vestibular Rehabilitation for Peripheral Vestibular Hypofunction: An Updated Clinical Practice Guideline From the Academy of Neurologic Physical Therapy of the American Physical Therapy Association | 75 | 67 | 77 | 81 | 50 | 75 |
| 94 | 2022 | KNGF Guideline on Oncology | 86 | 78 | 65 | 75 | 52 | 21 |
| 95 | 2022 | Vision Rehabilitation Preferred Practice Pattern | 83 | 61 | 81 | 81 | 54 | 79 |
| 96 | 2022 | Role of Physical Therapists in the Management of Individuals at Risk for or Diagnosed With Venous Thromboembolism: Evidence-Based Clinical Practice Guideline 2022 | 75 | 53 | 51 | 61 | 58 | 50 |
| 97 | 2022 | VA/DoD CLINICAL PRACTICE GUIDELINE FOR THE MANAGEMENT OF UPPER LIMB AMPUTATION REHABILITATION | 75 | 53 | 49 | 72 | 56 | 17 |
| 98 | 2022 | ISPAD Clinical Practice Consensus Guidelines 2022: Exercise in children and adolescents with diabetes | 64 | 50 | 29 | 53 | 33 | 42 |
| 99 | 2022 | Exercise-based knee and anterior cruciate ligament injury prevention.2022 | 72 | 58 | 54 | 83 | 44 | 38 |
| 100 | 2021 | Clinical Practice Guideline for Physical Therapist Management of People With Rheumatoid Arthritis | 75 | 61 | 70 | 78 | 48 | 92 |
| 101 | 2021 | Mobilization and Exercise Intervention for Patients With Multiple Myeloma: Clinical Practice Guidelines Endorsed by the Canadian Physiotherapy Association | 67 | 69 | 59 | 42 | 27 | 92 |
| 102 | 2021 | A Clinical Practice Guideline for the Use of Ankle-Foot Orthoses and Functional Electrical Stimulation Post-Stroke | 78 | 75 | 88 | 89 | 67 | 96 |
| 103 | 2021 | Physical Therapy Management of Older Adults With Hip Fracture | 78 | 67 | 79 | 75 | 69 | 67 |
| 104 | 2021 | KNGF Guideline on Low Back Pain and Lumbosacral Radicular Syndrome | 94 | 75 | 52 | 78 | 63 | 0 |
| 105 | 2021 | The CanPain SCI clinical practice guidelines for rehabilitation management of neuropathic pain after spinal cord injury: 2021 update | 83 | 72 | 82 | 86 | 40 | 92 |
| 106 | 2021 | VA/DoD CLINICAL PRACTICE GUIDELINE FOR THE MANAGEMENT AND REHABILITATION OF POST-ACUTE MILD TRAUMATIC BRAIN INJURY | 78 | 58 | 56 | 75 | 52 | 25 |
| 107 | 2021 | JCS/JACR 2021 Guideline on Rehabilitation in Patients With Cardiovascular Disease | 64 | 53 | 47 | 61 | 42 | 46 |
| 108 | 2020 | Chinese Stroke Association guidelines for clinical management of cerebrovascular disorders: executive summary and 2019 update of clinical management of stroke rehabilitation | 75 | 61 | 14 | 89 | 13 | 46 |
| 109 | 2020 | Physical Therapist Clinical Practice Guideline for the Management of Individuals With Heart Failure | 78 | 75 | 73 | 83 | 40 | 71 |
| 110 | 2020 | Pulmonary rehabilitation principles in SARS-COV-2 infection (COVID-19): A guideline for the acute and subacute rehabilitation | 75 | 58 | 49 | 89 | 13 | 79 |
| 111 | 2020 | Rehabilitation for adults with complex psychosis | 92 | 58 | 55 | 78 | 63 | 50 |
| 112 | 2020 | Physical Therapy Evaluation and Treatment After Concussion/Mild Traumatic Brain Injury | 89 | 69 | 86 | 92 | 71 | 100 |
| 113 | 2020 | Evidence-based national consensus: Recommendations for physiotherapy management in covid-19 in acute care indian setup | 78 | 50 | 46 | 75 | 27 | 38 |
| 114 | 2020 | Clinical Practice Guideline to Improve Locomotor Function Following Chronic Stroke, Incomplete Spinal Cord Injury, and Brain Injury | 83 | 75 | 77 | 97 | 58 | 79 |
| 115 | 2020 | A clinical practice guideline for physical therapy in patients with hip or knee osteoarthritis | 78 | 58 | 75 | 94 | 48 | 46 |
| 116 | 2020 | Canadian Stroke Best Practice Recommendations: Rehabilitation, Recovery, and Community Participation following Stroke | 69 | 53 | 60 | 72 | 40 | 92 |
| 117 | 2019 | The Turkish Society of Physical Medicine and Rehabilitation (TSPMR) guideline recommendations for the management of fibromyalgia syndrome | 61 | 39 | 44 | 94 | 10 | 79 |
| 118 | 2019 | The management of stroke rehabilitation: A synopsis of the 2019 U.S. Department of Veterans Affairs and U.S. Department of Defense clinical practice guideline | 67 | 44 | 40 | 69 | 19 | 63 |
| 119 | 2019 | Clinical Practice Guidelines for the Rehabilitation of Lower Limb Amputation: An Update from the Department of Veterans Affairs and Department of Defense | 75 | 53 | 54 | 75 | 25 | 50 |
| 120 | 2019 | Clinical practice guideline for cardiac rehabilitation in Korea: recommendations for cardiac rehabilitation and secondary prevention after acute coronary syndrome | 92 | 61 | 73 | 89 | 27 | 54 |
| 121 | 2019 | VA/DOD Clinical practice guideline for the management of stroke rehabilitation | 78 | 72 | 73 | 89 | 42 | 67 |
| 122 | 2019 | Occupational therapy for epidermolysis bullosa: Clinical practice guidelines | 78 | 50 | 45 | 56 | 31 | 83 |
| 123 | 2018 | The Ottawa Panel guidelines on programmes involving therapeutic exercise for the management of hand osteoarthritis | 72 | 61 | 45 | 81 | 10 | 58 |
| 124 | 2018 | Spinal Manipulative Therapy and Other Conservative Treatments for Low Back Pain: A Guideline From the Canadian Chiropractic Guideline Initiative | 100 | 58 | 69 | 83 | 71 | 88 |
| 125 | 2018 | Evidence- and Consensus-Based Guidelines for the Management of Communication and Swallowing Disorders Following Pediatric Traumatic Brain Injury | 67 | 50 | 61 | 89 | 17 | 38 |
| 126 | 2018 | Unique features of the INESSS-Onf rehabilitation guidelines for moderate to severe traumatic brain injury: Responding to users' needs | 61 | 58 | 65 | 94 | 15 | 42 |
| 127 | 2018 | A Core Set of Outcome Measures for Adults With Neurologic Conditions Undergoing Rehabilitation: A CLINICAL PRACTICE GUIDELINE | 67 | 100 | 77 | 89 | 33 | 8 |
| 128 | 2018 | KNGF guideline Rheumatoid arthritis | 89 | 61 | 42 | 67 | 42 | 0 |
| 129 | 2018 | KNGF guideline Osteoarthritis of the hip-knee | 72 | 67 | 58 | 78 | 71 | 0 |
| 130 | 2018 | 2016 SOSORT guidelines: orthopaedic and rehabilitation treatment of idiopathic scoliosis during growth | 78 | 50 | 75 | 89 | 67 | 92 |
| 131 | 2018 | Vision Rehabilitation Preferred Practice Pattern | 83 | 53 | 74 | 97 | 35 | 88 |
| 132 | 2018 | ISPAD Clinical Practice Consensus Guidelines 2018: Exercise in children and adolescents with diabetes | 75 | 47 | 40 | 86 | 42 | 17 |
| 133 | 2018 | Exercise-based knee and anterior cruciate ligament injury prevention 2018 | 81 | 64 | 50 | 69 | 40 | 88 |
| 134 | 2017 | Evidence-based (GRADE approach) Korean medicine clinical practice guidelines of manual acupuncture for the treatment of shoulder pain | 83 | 39 | 73 | 67 | 42 | 58 |
| 135 | 2017 | Clinical practice guidelines of acupuncture-moxibustion for cervical spondylotic radiculopath | 72 | 50 | 33 | 94 | 21 | 42 |
| 136 | 2017 | A Clinical Practice Guideline for the Management of Patients With Acute Spinal Cord Injury: Recommendations on the Type and Timing of Rehabilitation | 83 | 56 | 65 | 89 | 42 | 42 |
| 137 | 2017 | Cardiac Rehabilitation for Patients With Coronary Artery Disease: A Practical Guide to Enhance Patient Outcomes Through Continuity of Care | 56 | 22 | 15 | 67 | 0 | 33 |
| 138 | 2017 | The Ottawa panel clinical practice guidelines for the management of knee osteoarthritis. Part two: strengthening exercise programs | 44 | 39 | 38 | 78 | 0 | 67 |
| 139 | 2017 | Cardiac rehabilitation | 83 | 78 | 50 | 94 | 46 | 0 |
| 140 | 2017 | Australian and New Zealand Pulmonary Rehabilitation Guidelines | 72 | 67 | 40 | 83 | 13 | 83 |
| 141 | 2017 | General guidelines in the rehabilitation process for return to training after a sports injury | 50 | 22 | 4 | 39 | 4 | 17 |
| 142 | 2017 | The CanPain SCI Clinical Practice Guidelines for Rehabilitation Management of Neuropathic Pain after Spinal Cord: introduction, methodology and recommendation overview | 83 | 67 | 56 | 100 | 46 | 67 |
| 143 | 2017 | INESSS-ONF clinical practice guidelines for the rehabilitation of adults having sustained a moderate-to-severe TBI | 22 | 56 | 15 | 100 | 13 | 0 |
| 144 | 2017 | Sexual Rehabilitation After Treatment for Prostate Cancer—Part 1: Recommendations From the Fourth International Consultation for Sexual Medicine (ICSM 2015) | 61 | 28 | 17 | 83 | 8 | 33 |
| 145 | 2017 | Canadian stroke best practice recommendations: Stroke rehabilitation practice guidelines, update 2015 of stroke, sixth edition practice guidelines, update 2017 | 72 | 61 | 33 | 89 | 29 | 100 |
| 146 | 2017 | Canadian Stroke Best Practice Recommendations Telestroke Best Practice Guidelines Update 2017 | 83 | 64 | 67 | 64 | 25 | 83 |
| 147 | 2017 | Ottawa Panel Evidence-Based Clinical Practice Guidelines for Structured Physical Activity in the Management of Juvenile Idiopathic Arthritis | 83 | 78 | 72 | 94 | 48 | 83 |
| 148 | 2017 | Clinical Practice Guideline for Physical Therapy Assessment and Treatment in Patients With Nonspecific Neck Pain | 83 | 47 | 41 | 67 | 21 | 92 |
| 149 | 2017 | VA/DoD CLINICAL PRACTICE GUIDELINE FOR REHABILITATION OF INDIVIDUALS WITH LOWER LIMB AMPUTATION | 94 | 56 | 81 | 92 | 44 | 75 |
| 150 | 2016 | Speech and swallow rehabilitation in head and neck cancer: United Kingdom National Multidisciplinary Guidelines | 56 | 11 | 15 | 78 | 13 | 0 |
| 151 | 2016 | Vestibular Rehabilitation for Peripheral Vestibular Hypofunction: An Evidence-Based Clinical Practice Guideline: FROM THE AMERICAN PHYSICAL THERAPY ASSOCIATION NEUROLOGY SECTION | 94 | 78 | 79 | 100 | 58 | 92 |
| 152 | 2016 | Canadian stroke best practice recommendations: Stroke rehabilitation practice guidelines, update 2015 | 94 | 67 | 33 | 67 | 29 | 100 |
| 153 | 2016 | Guidelines for Adult Stroke Rehabilitation and Recovery: A Guideline for Healthcare Professionals from the American Heart Association/American Stroke Association | 89 | 61 | 29 | 72 | 38 | 75 |
| 154 | 2016 | KNGF Clinical Practice Guideline for Physical Therapy in patients with neck pain | 83 | 83 | 6 | 100 | 17 | 0 |
| 155 | 2016 | The CanPain SCI Clinical Practice Guidelines for Rehabilitation Management of Neuropathic Pain after Spinal Cord: Recommendations for treatment | 83 | 67 | 56 | 100 | 46 | 67 |
| 156 | 2016 | Evidence-based clinical practice update: practice guidelines for anterior cruciate ligament rehabilitation based on a systematic review and multidisciplinary consensus | 89 | 28 | 67 | 83 | 0 | 67 |
| 157 | 2016 | The CanPain SCI Clinical Practice Guidelines for Rehabilitation Management of Neuropathic Pain after Spinal Cord: screening and diagnosis recommendations | 78 | 64 | 55 | 100 | 46 | 67 |
| 158 | 2016 | Practice guidelines for program evaluation in community-based rehabilitation | 56 | 22 | 22 | 86 | 8 | 58 |
| 159 | 2016 | Vestibular rehabilitation for peripheral vestibular hypofunction: an evidence-based clinical practice guideline: from the American Physical Therapy Association Neurology Section | 83 | 50 | 49 | 92 | 44 | 46 |
| 160 | 2016 | Role of Physical Therapists in the Management of Individuals at Risk for or Diagnosed With Venous Thromboembolism: Evidence-Based Clinical Practice Guideline | 97 | 69 | 64 | 86 | 50 | 92 |
| 161 | 2016 | VA/DoD CLINICAL PRACTICE GUIDELINE FOR THE MANAGEMENT OF CONCUSSION-MILD TRAUMATIC BRAIN INJURY | 97 | 81 | 78 | 72 | 50 | 75 |
| 162 | 2016 | The CanPain SCI Clinical Practice Guideline for Rehabilitation Management of Neuropathic Pain after Spinal Cord: Recommendations for model systems of care | 94 | 61 | 64 | 72 | 56 | 71 |
| 163 | 2015 | Ottawa Panel evidence-based clinical practice guidelines for therapeutic exercise in the management of hip osteoarthritis | 100 | 61 | 68 | 67 | 0 | 67 |
| 164 | 2015 | Practice guidelines for cardiovascular fitness and strengthening exercise prescription after burn injury | 83 | 53 | 63 | 64 | 0 | 0 |
| 165 | 2015 | Exercise and Habitual Physical Activity for People with Cystic Fibrosis: Expert Consensus, Evidence-Based Guide for Advising Patients | 89 | 42 | 16 | 83 | 13 | 17 |
| 166 | 2015 | Exercise-based cardiac rehabilitation in patients with chronic heart failure: a Dutch practice guideline | 61 | 50 | 51 | 89 | 33 | 33 |
| 167 | 2015 | Stroke rehabilitation: long-term rehabilitation after stroke | 75 | 33 | 22 | 61 | 25 | 42 |
| 168 | 2014 | Guidelines for rehabilitation in patients with cardiovascular disease (JCS 2012) | 36 | 39 | 29 | 75 | 29 | 8 |
| 169 | 2014 | Physical activity exercise referral schemes | 81 | 78 | 58 | 53 | 58 | 8 |
| 170 | 2014 | KNGF Clinical Practice Guideline for Physical Therapy in patients with stroke | 72 | 53 | 41 | 69 | 54 | 42 |
| 171 | 2014 | Rehabilitation guidelines after rotator cuff repair | 33 | 28 | 3 | 36 | 13 | 0 |
| 172 | 2014 | VA/DoD CLINICAL PRACTICE GUIDELINE FOR THE MANAGEMENT OF UPPER LIMB AMPUTATION REHABILITATION | 81 | 69 | 64 | 75 | 58 | 25 |
| 173 | 2014 | ISPAD Clinical Practice Consensus Guidelines 2014. Exercise in children and adolescents with diabetes | 89 | 56 | 59 | 75 | 63 | 54 |
| 174 | 2014 | Clinical Competency Guidelines for Pulmonary Rehabilitation Professionals 2014 | 81 | 53 | 52 | 75 | 56 | 42 |
| 175 | 2013 | Brain injury rehabilitation in adults | 94 | 67 | 61 | 39 | 63 | 25 |
| 176 | 2013 | British Thoracic Society guideline on pulmonary rehabilitation in adults | 83 | 42 | 61 | 61 | 54 | 0 |
| 177 | 2013 | Exercise-based cardiac rehabilitation in patients with chronic heart failure: a Dutch practice guideline | 50 | 33 | 40 | 86 | 50 | 50 |
| 178 | 2013 | The 2013 Canadian Cardiovascular Society Heart Failure Management Guidelines Update: Focus on Rehabilitation and Exercise and Surgical Coronary Revascularization | 78 | 64 | 49 | 81 | 50 | 50 |
| 179 | 2012 | Expert opinion and key recommendations for the physical therapy and rehabilitation of patients with ankylosing spondylitis | 50 | 31 | 38 | 78 | 38 | 0 |
| 180 | 2012 | French Society of Cardiology guidelines for cardiac rehabilitation in adults | 25 | 14 | 19 | 67 | 8 | 25 |
| 181 | 2012 | Ottawa panel evidence-based clinical practice guidelines for aerobic walking programs in the management of osteoarthritis | 86 | 50 | 51 | 64 | 33 | 38 |
| 182 | 2012 | Ottawa Panel evidence-based clinical practice guidelines on therapeutic massage for neck pain | 72 | 39 | 31 | 64 | 38 | 8 |
| 183 | 2012 | Ottawa Panel evidence-based clinical practice guidelines on therapeutic massage for low back pain | 69 | 61 | 46 | 69 | 42 | 8 |
| 184 | 2011 | In-hospital rehabilitation after multiple joint procedures of the lower extremities in haemophilia patients: clinical guidelines for physical therapists | 39 | 33 | 43 | 67 | 46 | 50 |
| 185 | 2011 | Physiotherapy in rheumatoid arthritis: development of a practice guideline | 72 | 19 | 43 | 78 | 42 | 0 |
| 186 | 2011 | KNGF Guideline for Physical Therapy in patients with Osteoporosis | 58 | 19 | 19 | 58 | 38 | 0 |
| 187 | 2011 | KNGF Guideline for Physical Therapy in patients with Stress urinary incontinence | 53 | 22 | 21 | 58 | 38 | 0 |
| 188 | 2011 | KNGF Clinical Practice Guideline for physical therapy in patients undergoing cardiac rehabilitation | 61 | 22 | 21 | 61 | 38 | 0 |
| 189 | 2011 | S1 guideline: dermatological inpatient rehabilitation in adult atopic dermatitis | 50 | 28 | 9 | 28 | 13 | 0 |
| 190 | 2011 | 2011 SOSORT guidelines: Orthopaedic and rehabilitation treatment of idiopathic scoliosis during growth. Scoliosis. | 72 | 50 | 42 | 67 | 50 | 21 |
| 191 | 2010 | Optimizing pulmonary rehabilitation in chronic obstructive pulmonary disease--practical issues: a Canadian Thoracic Society Clinical Practice Guideline | 75 | 50 | 55 | 56 | 33 | 50 |
| 192 | 2010 | The American Society of Shoulder and Elbow Therapists' consensus rehabilitation guideline for arthroscopic anterior capsulolabral repair of the shoulder | 75 | 39 | 34 | 56 | 29 | 50 |
| 193 | 2010 | KNGF Guideline for Physical Therapy in patients with Osteoarthritis of the hip and knee | 72 | 33 | 34 | 58 | 50 | 33 |
| 194 | 2010 | Management of patients with stroke: rehabilitation, prevention and management of complications, and discharge planning : a national clinical guideline | 81 | 31 | 47 | 61 | 46 | 50 |
| 195 | 2009 | Rehabilitation after critical illness in adults | 64 | 25 | 26 | 56 | 25 | 17 |
| 196 | 2009 | Hip pain and mobility deficits--hip osteoarthritis: clinical practice guidelines linked to the international classification of functioning, disability, and health from the orthopaedic section of the American Physical Therapy Association | 64 | 44 | 42 | 47 | 50 | 58 |
| 197 | 2008 | Mental wellbeing in over 65s occupational therapy and physical activity interventions | 83 | 44 | 39 | 64 | 42 | 0 |
| 198 | 2008 | Neck pain: Clinical practice guidelines linked to the International Classification of Functioning, Disability, and Health from the Orthopedic Section of the American Physical Therapy Association | 67 | 42 | 43 | 72 | 33 | 8 |
| 199 | 2008 | KNGF Clinical Practice Guideline for physical therapy in patients with COPD | 72 | 28 | 33 | 69 | 50 | 25 |
| 200 | 2008 | Internal impingement in the tennis player: rehabilitation guidelines | 67 | 22 | 22 | 47 | 33 | 25 |
| 201 | 2008 | Ottawa Panel evidence-based clinical practice guidelines for strengthening exercises in the management of fibromyalgia | 64 | 42 | 28 | 83 | 31 | 8 |
| 202 | 2007 | Pulmonary rehabilitation: Joint ACCP/AACVPR evidence-based clinical practice guidelines | 69 | 42 | 47 | 53 | 29 | 42 |
| 203 | 2007 | Clinical Competency Guidelines for Pulmonary Rehabilitation Professionals: American Association of Cardiovascular and Pulmonary Rehabilitation Position Statement | 89 | 56 | 44 | 81 | 40 | 50 |
| 204 | 2006 | Ottawa panel evidence-based clinical practice guidelines for post-stroke rehabilitation | 67 | 47 | 43 | 61 | 21 | 50 |
| 205 | 2006 | KNGF Guideline for Physical Therapy in patients with acute ankle sprain | 67 | 39 | 38 | 67 | 46 | 25 |
| 206 | 2005 | Management of Adult Stroke Rehabilitation Care: a clinical practice guideline | 64 | 53 | 55 | 69 | 42 | 25 |
| 207 | 2005 | Evidence-Based Clinical Practice Guidelines for Interdisciplinary Rehabilitation of Chronic Nonmalignant Pain Syndrome Patients | 64 | 28 | 38 | 58 | 17 | 50 |
| 208 | 2005 | Italian guidelines on rehabilitation treatment of adolescents with scoliosis or other spinal deformities | 75 | 39 | 39 | 61 | 13 | 50 |
| 209 | 2005 | EFNS guidelines on cognitive rehabilitation: report of an EFNS task force | 64 | 42 | 50 | 72 | 25 | 50 |
| 210 | 2005 | Guidelines for rehabilitation of children with cerebral palsy | 58 | 39 | 39 | 61 | 25 | 50 |
| 211 | 2005 | Ottawa Panel evidence-based clinical practice guidelines for therapeutic exercises and manual therapy in the treatment of osteoarthritis | 86 | 72 | 66 | 58 | 38 | 42 |
| 212 | 2004 | Ottawa Panel Evidence-Based Clinical Practice Guidelines for Electrotherapy and Thermotherapy Interventions in the Management of Rheumatoid Arthritis in Adults | 67 | 42 | 40 | 58 | 25 | 67 |
| 213 | 2003 | Health professional's guide to rehabilitation of the patient with osteoporosis | 67 | 28 | 41 | 61 | 29 | 67 |
| 214 | 2003 | Occupational therapy and physiotherapy for the patient with burns: principles and management guidelines | 67 | 47 | 41 | 72 | 29 | 58 |
| 215 | 2003 | EFNS guidelines on cognitive rehabilitation report of an EFNS task force | 81 | 64 | 58 | 75 | 63 | 33 |
| 216 | 2002 | Clinical practice guideline for the physiotherapy of patients with whiplash-associated disorders | 64 | 53 | 48 | 56 | 25 | 67 |
| 217 | 2001 | Philadelphia Panel evidence-based clinical practice guidelines on selected rehabilitation interventions for low back pain | 61 | 33 | 36 | 36 | 25 | 50 |
| 218 | 2001 | Philadelphia Panel evidence-based clinical practice guidelines on selected rehabilitation interventions for knee pain | 61 | 28 | 38 | 42 | 29 | 50 |
| 219 | 2001 | Upper Extremity Rehabilitation in Women with Breast Cancer after Axillary Dissection: Clinical Practice Guidelines | 56 | 42 | 43 | 53 | 38 | 42 |
| 220 | 2001 | Pulmonary rehabilitation British Thoracic Society Standards of Care Subcommittee on Pulmonary Rehabilitation | 58 | 39 | 50 | 44 | 38 | 58 |
| 221 | 2001 | Philadelphia panel evidence-based clinical practice guidelines on selected rehabilitation interventions for shoulder pain | 58 | 47 | 47 | 44 | 42 | 58 |
| 222 | 2001 | Philadelphia Panel evidence-based clinical practice guidelines on selected rehabilitation interventions for neck pain | 56 | 36 | 65 | 50 | 42 | 58 |
| 223 | 2001 | Philadelphia Panel Evidence-Based Clinical Practice Guidelines on Selected Rehabilitation Interventions: Overview and Methodology | 75 | 42 | 39 | 69 | 29 | 42 |
| 224 | 1999 | Therapeutic Exercise Guidelines for Rehabilitating Lumbar Spine Injuries in Athletes | 56 | 36 | 55 | 58 | 38 | 50 |
| 225 | 1996 | Cardiac rehabilitation in the United Kingdom: guidelines and audit standards | 64 | 36 | 28 | 53 | 42 | 50 |
| 226 | 1996 | Cardiac Rehabilitation as Secondary Prevention: Quick Reference Guide for Clinicians | 61 | 33 | 39 | 61 | 38 | 67 |
| 227 | 1992 | Guidelines for Cognitive Rehabilitation | 58 | 42 | 45 | 44 | 46 | 58 |

**Table S8. Reporting of 227 CPGs using RIGHT checklist**

| ID | Title | Basic information | | | | | | | | Background | | | | | | | | Evidence | | | | | | Recommendations | | | | | | | | Review and quality assurance | | | Funding and declaration and management of interests | | | | | Other information | | |
| --- | --- | --- | --- | --- | --- | --- | --- | --- | --- | --- | --- | --- | --- | --- | --- | --- | --- | --- | --- | --- | --- | --- | --- | --- | --- | --- | --- | --- | --- | --- | --- | --- | --- | --- | --- | --- | --- | --- | --- | --- | --- | --- |
|  |  | 1a | 1b | 1c | 2 | 3 | 4 | 5 | 6 | | 7a | 7b | 8a | 8b | 9a | 9b | 10a | | 10b | 11a | 11b | 12 | 13a | | 13b | 13c | 14a | 14b | 14c | 15 | 16 | | 17 | 18a | | 18b | 19a | 19b | 20 | | 21 | 22 |
| 1 | 中国膝骨关节炎康复治疗指南（2023版） | 1 | 1 | 1 | 1 | 1 | 1 | 1 | 1 | | 1 | 0 | 1 | 1 | 1 | 1 | 1 | | 1 | 1 | 1 | 1 | 1 | | 1 | 1 | 1 | 1 | 0 | 1 | 1 | | 1 | 1 | | 1 | 1 | 0 | 1 | | 1 | 1 |
| 2 | 新型冠状病毒感染恢复期常见症中西医结合管理循证实践指南 | 1 | 0 | 1 | 0 | 1 | 1 | 1 | 1 | | 1 | 0 | 1 | 0 | 1 | 1 | 1 | | 1 | 1 | 1 | 1 | 1 | | 1 | 1 | 1 | 1 | 1 | 1 | 1 | | 0 | 1 | | 1 | 1 | 0 | 0 | | 0 | 0 |
| 3 | 中轴脊柱关节炎/强直性脊柱炎康复实践指南(2023版) | 1 | 1 | 1 | 0 | 1 | 1 | 0 | 1 | | 1 | 0 | 1 | 0 | 1 | 1 | 1 | | 0 | 1 | 1 | 1 | 1 | | 1 | 1 | 0 | 0 | 0 | 1 | 1 | | 0 | 1 | | 0 | 0 | 0 | 0 | | 1 | 1 |
| 4 | 特发性肺纤维化中医康复指南(2021-10-21) | 1 | 1 | 1 | 0 | 1 | 1 | 1 | 1 | | 1 | 0 | 1 | 0 | 1 | 0 | 1 | | 0 | 1 | 1 | 1 | 1 | | 1 | 0 | 0 | 0 | 0 | 1 | 0 | | 1 | 1 | | 0 | 1 | 0 | 0 | | 0 | 0 |
| 5 | 膝骨关节炎针刀临床诊疗指南 | 1 | 0 | 1 | 1 | 1 | 1 | 0 | 1 | | 1 | 0 | 1 | 1 | 1 | 0 | 0 | | 0 | 0 | 0 | 0 | 1 | | 1 | 1 | 0 | 0 | 0 | 0 | 0 | | 0 | 1 | | 0 | 0 | 0 | 1 | | 1 | 1 |
| 6 | 中国骨肌疾病体外冲击波疗法指南(2023年版) | 1 | 1 | 1 | 0 | 1 | 1 | 0 | 1 | | 1 | 1 | 1 | 0 | 1 | 1 | 1 | | 0 | 1 | 1 | 1 | 0 | | 1 | 1 | 0 | 0 | 0 | 1 | 0 | | 1 | 1 | | 0 | 0 | 0 | 1 | | 0 | 0 |
| 7 | 中国吞咽障碍康复管理指南(2023版) | 1 | 1 | 1 | 0 | 1 | 0 | 1 | 1 | | 1 | 0 | 1 | 1 | 1 | 1 | 1 | | 0 | 1 | 1 | 1 | 1 | | 1 | 1 | 0 | 1 | 0 | 1 | 0 | | 1 | 1 | | 0 | 1 | 0 | 1 | | 1 | 0 |
| 8 | 脊髓损伤康复治疗临床实践指南 | 1 | 0 | 1 | 0 | 1 | 1 | 1 | 1 | | 1 | 0 | 1 | 0 | 1 | 1 | 0 | | 0 | 1 | 1 | 1 | 1 | | 1 | 1 | 1 | 0 | 0 | 0 | 0 | | 1 | 1 | | 0 | 0 | 0 | 0 | | 1 | 0 |
| 9 | 中国老年人肌少症临床康复治疗指南 | 1 | 0 | 1 | 0 | 1 | 0 | 1 | 0 | | 1 | 0 | 0 | 0 | 1 | 1 | 0 | | 0 | 0 | 0 | 0 | 0 | | 0 | 0 | 0 | 0 | 0 | 0 | 0 | | 0 | 0 | | 0 | 1 | 0 | 1 | | 1 | 0 |
| 10 | 周围神经损伤居家康复指南 | 1 | 0 | 1 | 0 | 1 | 0 | 1 | 1 | | 1 | 0 | 1 | 1 | 1 | 1 | 1 | | 0 | 1 | 1 | 1 | 1 | | 1 | 1 | 0 | 0 | 0 | 1 | 0 | | 0 | 0 | | 0 | 0 | 0 | 1 | | 1 | 1 |
| 11 | 中国脑性瘫痪康复指南(2022) | 1 | 1 | 1 | 0 | 1 | 1 | 1 | 1 | | 1 | 0 | 0 | 0 | 1 | 0 | 0 | | 0 | 1 | 1 | 1 | 0 | | 0 | 1 | 1 | 1 | 1 | 1 | 1 | | 1 | 1 | | 1 | 1 | 0 | 0 | | 0 | 0 |
| 12 | 腰椎间盘突出症诊治与康复管理指南 | 1 | 0 | 1 | 0 | 1 | 1 | 0 | 1 | | 1 | 0 | 0 | 0 | 1 | 1 | 0 | | 0 | 1 | 1 | 1 | 1 | | 1 | 1 | 0 | 0 | 0 | 1 | 0 | | 1 | 1 | | 1 | 1 | 0 | 1 | | 1 | 1 |
| 13 | 骨质疏松性椎体压缩骨折椎体强化术后康复治疗指南（2022版） | 1 | 1 | 1 | 1 | 1 | 1 | 1 | 1 | | 1 | 0 | 0 | 0 | 0 | 1 | 0 | | 0 | 1 | 1 | 1 | 1 | | 1 | 1 | 0 | 0 | 0 | 0 | 0 | | 0 | 0 | | 0 | 1 | 0 | 1 | | 1 | 0 |
| 14 | 脑卒中中西医结合康复临床循证实践指南 | 1 | 0 | 1 | 0 | 1 | 1 | 1 | 1 | | 1 | 1 | 1 | 1 | 1 | 1 | 1 | | 1 | 1 | 1 | 1 | 1 | | 1 | 1 | 0 | 0 | 1 | 1 | 1 | | 1 | 1 | | 0 | 1 | 1 | 1 | | 1 | 1 |
| 15 | 脊髓损伤中西医结合康复临床实践指南 | 1 | 0 | 1 | 0 | 1 | 1 | 0 | 1 | | 0 | 0 | 0 | 1 | 0 | 0 | 0 | | 0 | 0 | 0 | 1 | 1 | | 1 | 1 | 0 | 0 | 0 | 0 | 0 | | 0 | 1 | | 0 | 0 | 0 | 1 | | 1 | 1 |
| 16 | 中国2型糖尿病运动治疗指南（2024版） | 1 | 0 | 1 | 1 | 1 | 1 | 1 | 1 | | 1 | 0 | 1 | 0 | 1 | 1 | 1 | | 1 | 1 | 1 | 1 | 1 | | 1 | 1 | 1 | 1 | 0 | 1 | 1 | | 1 | 1 | | 1 | 1 | 0 | 1 | | 1 | 1 |
| 17 | 中国冠心病康复循证实践指南(2024版)第一部分 | 0 | 0 | 1 | 0 | 0 | 0 | 1 | 0 | | 1 | 0 | 1 | 0 | 1 | 1 | 1 | | 1 | 1 | 1 | 1 | 1 | | 1 | 1 | 1 | 1 | 1 | 1 | 1 | | 0 | 0 | | 0 | 1 | 1 | 1 | | 1 | 1 |
| 18 | 中医康复临床实践指南·儿童青少年特发性脊柱侧弯 | 1 | 0 | 1 | 1 | 1 | 1 | 1 | 1 | | 1 | 0 | 1 | 0 | 0 | 1 | 1 | | 1 | 0 | 0 | 1 | 1 | | 1 | 1 | 1 | 1 | 1 | 0 | 0 | | 0 | 1 | | 1 | 1 | 0 | 1 | | 0 | 0 |
| 19 | 中医康复临床实践指南·悬吊推拿运动技术 | 1 | 0 | 1 | 1 | 1 | 1 | 1 | 1 | | 1 | 0 | 1 | 0 | 1 | 1 | 0 | | 1 | 1 | 1 | 1 | 1 | | 1 | 1 | 1 | 1 | 0 | 1 | 1 | | 0 | 1 | | 1 | 1 | 1 | 1 | | 1 | 1 |
| 20 | 中国纤维肌痛康复指南(2021) | 1 | 0 | 1 | 0 | 1 | 1 | 1 | 1 | | 1 | 0 | 1 | 0 | 1 | 1 | 0 | | 0 | 0 | 1 | 0 | 1 | | 1 | 1 | 0 | 0 | 0 | 0 | 0 | | 1 | 0 | | 0 | 1 | 0 | 1 | | 0 | 1 |
| 21 | 改良太极功法临床实践指南·脑卒中运动障碍 | 1 | 0 | 1 | 1 | 1 | 1 | 1 | 1 | | 1 | 0 | 1 | 1 | 1 | 1 | 0 | | 1 | 1 | 1 | 1 | 1 | | 1 | 1 | 1 | 1 | 1 | 1 | 1 | | 0 | 1 | | 0 | 1 | 1 | 1 | | 1 | 1 |
| 22 | 中国膝骨关节炎基层诊疗与康复指南(2022版) | 0 | 0 | 1 | 0 | 0 | 0 | 1 | 1 | | 1 | 0 | 1 | 1 | 1 | 1 | 1 | | 1 | 1 | 1 | 1 | 1 | | 1 | 1 | 1 | 1 | 1 | 1 | 1 | | 1 | 1 | | 0 | 1 | 1 | 1 | | 1 | 1 |
| 23 | 中国非特异性腰背痛临床诊疗指南 | 1 | 0 | 1 | 1 | 1 | 1 | 1 | 1 | | 1 | 0 | 1 | 0 | 0 | 1 | 0 | | 0 | 1 | 1 | 1 | 1 | | 1 | 1 | 1 | 1 | 1 | 1 | 1 | | 1 | 1 | | 0 | 1 | 1 | 1 | | 1 | 1 |
| 24 | 冠心病心脏康复基层合理用药指南 | 1 | 0 | 1 | 0 | 1 | 1 | 1 | 1 | | 0 | 0 | 0 | 0 | 1 | 0 | 0 | | 0 | 0 | 0 | 0 | 1 | | 1 | 0 | 0 | 0 | 0 | 0 | 0 | | 0 | 0 | | 0 | 1 | 0 | 1 | | 1 | 1 |
| 25 | 冠心病心脏康复基层指南（2020年） | 1 | 1 | 1 | 0 | 1 | 1 | 1 | 1 | | 0 | 1 | 0 | 1 | 1 | 0 | 0 | | 0 | 0 | 0 | 0 | 1 | | 1 | 0 | 0 | 0 | 0 | 0 | 0 | | 0 | 0 | | 0 | 1 | 0 | 1 | | 1 | 1 |
| 26 | 蒙医康复临床实践指南·脑梗死 | 1 | 0 | 1 | 0 | 1 | 1 | 0 | 1 | | 0 | 0 | 0 | 1 | 0 | 0 | 0 | | 0 | 0 | 0 | 0 | 1 | | 1 | 0 | 0 | 0 | 0 | 0 | 0 | | 0 | 1 | | 0 | 0 | 0 | 1 | | 1 | 1 |
| 27 | 中医康复临床实践指南·不完全性截瘫 | 1 | 0 | 1 | 0 | 1 | 1 | 0 | 1 | | 0 | 0 | 0 | 1 | 0 | 0 | 1 | | 0 | 0 | 0 | 0 | 1 | | 1 | 1 | 0 | 0 | 0 | 0 | 0 | | 0 | 1 | | 0 | 0 | 0 | 1 | | 0 | 0 |
| 28 | 中医康复临床实践指南·缺血性脑卒中(脑梗死) | 1 | 0 | 1 | 0 | 1 | 1 | 0 | 1 | | 0 | 0 | 0 | 1 | 0 | 0 | 1 | | 0 | 0 | 0 | 0 | 1 | | 1 | 1 | 0 | 0 | 0 | 0 | 0 | | 0 | 1 | | 0 | 0 | 0 | 1 | | 1 | 1 |
| 29 | 中医康复临床实践指南·腰痛(腰椎间盘突出症) | 1 | 0 | 1 | 0 | 1 | 1 | 0 | 1 | | 0 | 0 | 0 | 1 | 0 | 0 | 1 | | 0 | 0 | 0 | 0 | 1 | | 1 | 1 | 0 | 0 | 0 | 0 | 0 | | 0 | 1 | | 0 | 0 | 0 | 0 | | 0 | 0 |
| 30 | 尘肺病康复专家共识（2021版） | 1 | 1 | 1 | 0 | 1 | 1 | 1 | 1 | | 1 | 0 | 1 | 0 | 1 | 1 | 1 | | 0 | 1 | 1 | 1 | 0 | | 1 | 1 | 0 | 0 | 0 | 1 | 0 | | 0 | 1 | | 0 | 1 | 0 | 1 | | 1 | 1 |
| 31 | 慢性阻塞性肺疾病临床康复循证实践指南 | 1 | 0 | 1 | 0 | 1 | 1 | 1 | 0 | | 1 | 0 | 1 | 0 | 1 | 1 | 1 | | 0 | 1 | 1 | 1 | 1 | | 1 | 1 | 1 | 1 | 1 | 1 | 1 | | 1 | 1 | | 1 | 1 | 0 | 0 | | 0 | 0 |
| 32 | 慢性阻塞性肺疾病中医肺康复临床应用指南 | 1 | 0 | 1 | 0 | 1 | 1 | 1 | 0 | | 0 | 0 | 1 | 1 | 1 | 0 | 0 | | 0 | 1 | 1 | 1 | 1 | | 1 | 1 | 1 | 1 | 0 | 0 | 1 | | 0 | 1 | | 0 | 1 | 0 | 0 | | 1 | 1 |
| 33 | 脑性瘫痪儿童水中运动治疗临床实践指南 | 1 | 0 | 1 | 0 | 1 | 1 | 1 | 0 | | 1 | 0 | 1 | 1 | 1 | 1 | 1 | | 0 | 1 | 1 | 1 | 1 | | 1 | 1 | 0 | 0 | 0 | 0 | 1 | | 0 | 1 | | 1 | 1 | 0 | 0 | | 1 | 0 |
| 34 | 中国慢性呼吸道疾病呼吸康复管理指南（2021年） | 1 | 1 | 1 | 0 | 1 | 1 | 1 | 1 | | 1 | 0 | 1 | 0 | 1 | 1 | 0 | | 0 | 1 | 1 | 1 | 1 | | 1 | 1 | 0 | 0 | 0 | 1 | 1 | | 0 | 1 | | 0 | 1 | 0 | 0 | | 1 | 0 |
| 35 | 中国低视力康复临床指南（2021） | 1 | 1 | 1 | 0 | 0 | 1 | 1 | 0 | | 0 | 0 | 1 | 0 | 1 | 1 | 0 | | 0 | 0 | 0 | 0 | 0 | | 0 | 0 | 0 | 0 | 0 | 1 | 0 | | 1 | 1 | | 0 | 1 | 0 | 0 | | 0 | 1 |
| 36 | 中医康复临床实践指南·类风湿关节炎 | 1 | 0 | 1 | 0 | 1 | 1 | 0 | 0 | | 0 | 0 | 1 | 1 | 0 | 0 | 1 | | 0 | 0 | 0 | 0 | 1 | | 1 | 1 | 0 | 0 | 0 | 0 | 0 | | 1 | 1 | | 0 | 0 | 0 | 0 | | 0 | 0 |
| 37 | 中医康复临床实践指南·膝骨关节炎 | 1 | 0 | 1 | 0 | 1 | 1 | 0 | 0 | | 0 | 0 | 0 | 1 | 0 | 0 | 1 | | 0 | 0 | 0 | 0 | 1 | | 1 | 1 | 0 | 0 | 0 | 0 | 0 | | 1 | 1 | | 0 | 0 | 0 | 0 | | 0 | 0 |
| 38 | 中医康复临床实践指南·项痹(颈椎病) | 1 | 0 | 1 | 0 | 1 | 1 | 0 | 0 | | 0 | 0 | 1 | 1 | 0 | 0 | 1 | | 0 | 0 | 0 | 0 | 1 | | 1 | 1 | 0 | 0 | 0 | 0 | 0 | | 1 | 1 | | 0 | 0 | 0 | 0 | | 0 | 0 |
| 39 | 中医康复临床实践指南·心肺康复 | 1 | 0 | 1 | 0 | 1 | 1 | 0 | 0 | | 0 | 0 | 1 | 1 | 0 | 0 | 1 | | 0 | 0 | 0 | 0 | 1 | | 1 | 1 | 0 | 0 | 0 | 0 | 0 | | 1 | 1 | | 0 | 0 | 0 | 0 | | 0 | 0 |
| 40 | 新型冠状病毒肺炎恢复期中西医结合康复指南（第一版） | 1 | 0 | 1 | 0 | 0 | 0 | 0 | 1 | | 1 | 1 | 0 | 1 | 1 | 1 | 0 | | 0 | 0 | 0 | 0 | 1 | | 1 | 0 | 0 | 0 | 0 | 0 | 1 | | 1 | 0 | | 0 | 0 | 0 | 0 | | 0 | 0 |
| 41 | 新型冠状病毒肺炎中医康复专家共识(第一版) | 1 | 0 | 1 | 0 | 1 | 1 | 0 | 1 | | 1 | 0 | 1 | 0 | 1 | 1 | 0 | | 0 | 1 | 1 | 1 | 1 | | 1 | 0 | 1 | 1 | 0 | 1 | 0 | | 1 | 1 | | 0 | 1 | 0 | 0 | | 0 | 0 |
| 42 | 中西医结合康复临床实践指南·认知障碍 | 1 | 0 | 1 | 0 | 1 | 1 | 1 | 1 | | 0 | 0 | 0 | 1 | 0 | 0 | 0 | | 0 | 0 | 0 | 0 | 0 | | 0 | 1 | 0 | 0 | 0 | 0 | 0 | | 0 | 1 | | 0 | 0 | 0 | 0 | | 0 | 0 |
| 43 | 危重症新型冠状病毒肺炎患者后ICU综合征呼吸康复推荐意见 | 1 | 0 | 1 | 0 | 1 | 1 | 1 | 1 | | 1 | 0 | 0 | 0 | 1 | 1 | 0 | | 1 | 1 | 1 | 0 | 1 | | 1 | 0 | 0 | 0 | 0 | 0 | 0 | | 1 | 1 | | 0 | 1 | 0 | 0 | | 1 | 1 |
| 44 | 中国骨肌疾病体外冲击波疗法指南(2019年版) | 1 | 1 | 1 | 0 | 1 | 1 | 0 | 1 | | 1 | 1 | 1 | 0 | 1 | 1 | 0 | | 1 | 1 | 1 | 1 | 1 | | 1 | 1 | 0 | 0 | 0 | 0 | 0 | | 1 | 1 | | 0 | 0 | 0 | 0 | | 1 | 0 |
| 45 | 脑卒中水中运动治疗中国循证临床实践指南(2019版) | 1 | 1 | 1 | 0 | 1 | 1 | 1 | 1 | | 1 | 1 | 1 | 1 | 0 | 1 | 0 | | 1 | 1 | 1 | 1 | 1 | | 1 | 1 | 1 | 1 | 1 | 1 | 1 | | 1 | 1 | | 1 | 1 | 0 | 0 | | 1 | 0 |
| 46 | 中医康复临床实践指南·脑卒中 | 1 | 0 | 1 | 0 | 1 | 1 | 1 | 1 | | 0 | 0 | 0 | 1 | 0 | 0 | 1 | | 0 | 0 | 0 | 0 | 1 | | 1 | 1 | 0 | 0 | 0 | 0 | 0 | | 1 | 1 | | 0 | 0 | 0 | 0 | | 0 | 0 |
| 47 | 骨质疏松症康复指南（上）(下) | 1 | 0 | 1 | 1 | 1 | 1 | 1 | 1 | | 1 | 0 | 1 | 1 | 1 | 1 | 1 | | 0 | 1 | 1 | 1 | 1 | | 1 | 1 | 1 | 1 | 0 | 1 | 1 | | 1 | 1 | | 1 | 1 | 0 | 0 | | 1 | 0 |
| 48 | 物理技术辅助脑卒中康复的临床指南 | 1 | 0 | 1 | 0 | 0 | 1 | 1 | 1 | | 1 | 0 | 0 | 0 | 1 | 1 | 0 | | 0 | 0 | 0 | 0 | 0 | | 0 | 0 | 0 | 0 | 0 | 0 | 0 | | 0 | 0 | | 0 | 1 | 0 | 0 | | 1 | 0 |
| 49 | 急性脊柱脊髓损伤围术期管理临床指南 | 1 | 0 | 1 | 0 | 0 | 1 | 1 | 1 | | 1 | 0 | 0 | 0 | 0 | 1 | 0 | | 1 | 1 | 1 | 1 | 1 | | 1 | 1 | 0 | 0 | 0 | 1 | 1 | | 1 | 0 | | 0 | 1 | 0 | 0 | | 0 | 1 |
| 50 | 中医骨伤科临床诊疗指南·人工髋关节置换围手术期康复专家共识 | 1 | 0 | 1 | 0 | 1 | 1 | 0 | 1 | | 1 | 0 | 1 | 1 | 1 | 1 | 0 | | 1 | 1 | 1 | 1 | 0 | | 1 | 0 | 1 | 1 | 1 | 1 | 1 | | 1 | 1 | | 1 | 1 | 0 | 0 | | 0 | 0 |
| 51 | 中国脑卒中早期康复治疗指南 | 1 | 0 | 1 | 0 | 1 | 1 | 1 | 1 | | 1 | 0 | 1 | 1 | 1 | 1 | 0 | | 0 | 0 | 0 | 0 | 1 | | 1 | 1 | 0 | 0 | 0 | 0 | 0 | | 0 | 0 | | 0 | 0 | 0 | 0 | | 0 | 0 |
| 52 | 中国脑性瘫痪康复指南(2015) | 1 | 1 | 1 | 0 | 0 | 1 | 1 | 1 | | 0 | 0 | 0 | 1 | 1 | 1 | 1 | | 1 | 1 | 1 | 1 | 1 | | 1 | 1 | 0 | 0 | 0 | 1 | 1 | | 0 | 1 | | 1 | 1 | 0 | 0 | | 0 | 1 |
| 53 | 脊髓损伤患者泌尿系管理与临床康复指南 | 1 | 0 | 1 | 0 | 1 | 0 | 1 | 1 | | 1 | 1 | 0 | 1 | 0 | 1 | 0 | | 0 | 0 | 0 | 0 | 1 | | 1 | 0 | 0 | 0 | 0 | 0 | 0 | | 0 | 1 | | 0 | 0 | 0 | 0 | | 0 | 0 |
| 54 | 烧伤康复治疗指南(2013版) | 1 | 1 | 1 | 0 | 1 | 0 | 1 | 1 | | 1 | 0 | 0 | 0 | 0 | 1 | 0 | | 0 | 0 | 0 | 0 | 0 | | 1 | 0 | 0 | 0 | 0 | 0 | 0 | | 0 | 0 | | 0 | 0 | 0 | 0 | | 0 | 0 |
| 55 | 中国脑卒中康复治疗指南(2011完全版) | 1 | 1 | 1 | 0 | 1 | 0 | 1 | 1 | | 1 | 1 | 0 | 1 | 0 | 0 | 0 | | 0 | 0 | 0 | 1 | 1 | | 1 | 1 | 0 | 0 | 0 | 0 | 0 | | 1 | 1 | | 0 | 0 | 0 | 0 | | 0 | 0 |
| 56 | 中国卒中康复治疗指南简化版 | 1 | 0 | 1 | 0 | 1 | 1 | 1 | 1 | | 0 | 1 | 0 | 0 | 0 | 0 | 0 | | 0 | 0 | 0 | 1 | 1 | | 1 | 1 | 0 | 0 | 0 | 0 | 0 | | 0 | 0 | | 0 | 0 | 0 | 0 | | 0 | 0 |
| 57 | 地震伤员康复治疗指南 | 1 | 0 | 1 | 0 | 0 | 0 | 1 | 1 | | 0 | 0 | 1 | 0 | 1 | 1 | 0 | | 0 | 0 | 0 | 0 | 1 | | 1 | 0 | 0 | 0 | 0 | 0 | 0 | | 0 | 0 | | 0 | 0 | 0 | 0 | | 0 | 0 |
| 58 | 肝病患者康复指南 | 1 | 0 | 1 | 0 | 0 | 0 | 1 | 0 | | 0 | 0 | 0 | 0 | 0 | 1 | 0 | | 0 | 0 | 0 | 0 | 0 | | 0 | 0 | 0 | 0 | 0 | 0 | 0 | | 0 | 0 | | 0 | 0 | 0 | 0 | | 0 | 0 |
| 59 | 创伤性脊髓损伤康复指南与规范 | 1 | 1 | 1 | 1 | 1 | 1 | 1 | 1 | | 1 | 0 | 1 | 1 | 1 | 0 | 0 | | 0 | 0 | 0 | 0 | 1 | | 1 | 0 | 0 | 0 | 0 | 0 | 0 | | 0 | 1 | | 0 | 1 | 0 | 0 | | 1 | 1 |
| 60 | 儿童发育性协调障碍的早期干预和康复指南(2025) | 1 | 1 | 1 | 1 | 0 | 1 | 1 | 1 | | 1 | 0 | 1 | 1 | 1 | 0 | 1 | | 1 | 1 | 1 | 1 | 1 | | 1 | 1 | 0 | 0 | 0 | 1 | 1 | | 1 | 1 | | 0 | 1 | 0 | 0 | | 0 | 0 |
| 61 | 儿童柔性扁平足康复管理指南(2025) | 1 | 1 | 1 | 1 | 0 | 1 | 1 | 1 | | 1 | 0 | 1 | 1 | 1 | 0 | 1 | | 1 | 1 | 1 | 1 | 1 | | 1 | 1 | 0 | 0 | 0 | 1 | 1 | | 1 | 1 | | 0 | 1 | 0 | 0 | | 1 | 1 |
| 62 | 儿童重症早期康复介入的临床实践指南（2025） | 1 | 1 | 1 | 1 | 0 | 1 | 1 | 1 | | 1 | 0 | 1 | 1 | 1 | 0 | 1 | | 1 | 1 | 1 | 1 | 1 | | 1 | 1 | 0 | 0 | 0 | 1 | 0 | | 0 | 1 | | 0 | 1 | 0 | 0 | | 1 | 1 |
| 63 | 慢性气道疾病康复基层指南（2025年） | 1 | 1 | 1 | 1 | 1 | 1 | 1 | 1 | | 1 | 0 | 1 | 1 | 1 | 0 | 0 | | 0 | 0 | 0 | 1 | 1 | | 1 | 1 | 0 | 0 | 0 | 0 | 0 | | 0 | 1 | | 0 | 1 | 0 | 0 | | 1 | 0 |
| 64 | 中国慢性呼吸疾病呼吸康复指南（2026版 | 1 | 1 | 1 | 1 | 0 | 1 | 1 | 1 | | 1 | 0 | 1 | 1 | 1 | 1 | 1 | | 1 | 1 | 1 | 1 | 1 | | 1 | 1 | 1 | 1 | 1 | 1 | 1 | | 1 | 1 | | 0 | 1 | 0 | 1 | | 1 | 1 |
| 65 | Physical Therapist Management of Glenohumeral Joint Osteoarthritis: A Clinical Practice Guideline from the American Physical Therapy Association | 1 | 0 | 1 | 1 | 1 | 1 | 1 | 1 | | 1 | 0 | 1 | 0 | 1 | 1 | 1 | | 1 | 1 | 1 | 1 | 1 | | 1 | 1 | 1 | 1 | 0 | 1 | 1 | | 1 | 1 | | 1 | 1 | 0 | 1 | | 1 | 1 |
| 66 | Stroke rehabilitation in adults | 0 | 0 | 1 | 0 | 0 | 0 | 1 | 1 | | 1 | 0 | 1 | 0 | 1 | 1 | 1 | | 1 | 1 | 1 | 1 | 1 | | 1 | 1 | 1 | 1 | 1 | 1 | 1 | | 1 | 0 | | 0 | 1 | 1 | 1 | | 0 | 0 |
| 67 | Acupuncture for treatment of knee osteoarthritis: A clinical practice guideline | 1 | 0 | 1 | 1 | 1 | 1 | 1 | 1 | | 1 | 0 | 1 | 0 | 0 | 1 | 1 | | 1 | 0 | 0 | 1 | 1 | | 1 | 1 | 1 | 1 | 1 | 0 | 0 | | 1 | 1 | | 1 | 1 | 0 | 0 | | 1 | 1 |
| 68 | Physical Therapist Management of Parkinson Disease: A Clinical Practice Guideline From the American Physical Therapy Association | 1 | 0 | 1 | 1 | 1 | 1 | 1 | 1 | | 1 | 0 | 1 | 0 | 1 | 1 | 0 | | 1 | 1 | 1 | 1 | 1 | | 1 | 1 | 1 | 1 | 0 | 1 | 1 | | 1 | 1 | | 1 | 1 | 1 | 1 | | 1 | 1 |
| 69 | Brazilian practice guidelines for stroke rehabilitation | 1 | 0 | 1 | 0 | 1 | 1 | 1 | 1 | | 1 | 0 | 1 | 0 | 1 | 1 | 0 | | 0 | 0 | 1 | 0 | 1 | | 1 | 1 | 0 | 0 | 0 | 0 | 0 | | 0 | 0 | | 0 | 1 | 0 | 1 | | 1 | 0 |
| 70 | Women-Focused Cardiovascular Rehabilitation: An International Council of Cardiovascular Prevention and Rehabilitation Clinical Practice Guideline | 1 | 0 | 1 | 1 | 1 | 1 | 1 | 1 | | 1 | 0 | 1 | 0 | 1 | 1 | 0 | | 1 | 1 | 1 | 1 | 1 | | 1 | 1 | 1 | 1 | 1 | 1 | 1 | | 1 | 1 | | 0 | 1 | 1 | 1 | | 1 | 1 |
| 71 | Rehabilitation after traumatic injury | 0 | 0 | 1 | 0 | 0 | 0 | 1 | 1 | | 1 | 1 | 1 | 0 | 1 | 1 | 1 | | 1 | 1 | 1 | 1 | 1 | | 1 | 1 | 1 | 1 | 1 | 1 | 1 | | 1 | 1 | | 0 | 1 | 1 | 1 | | 1 | 1 |
| 72 | Vestibular Rehabilitation for Peripheral Vestibular Hypofunction: An Updated Clinical Practice Guideline From the Academy of Neurologic Physical Therapy of the American Physical Therapy Association | 1 | 0 | 1 | 1 | 1 | 1 | 1 | 1 | | 1 | 0 | 1 | 0 | 0 | 1 | 0 | | 0 | 1 | 1 | 1 | 1 | | 1 | 1 | 1 | 1 | 1 | 1 | 1 | | 1 | 1 | | 0 | 1 | 1 | 1 | | 0 | 1 |
| 73 | KNGF Guideline on Oncology | 1 | 0 | 0 | 0 | 1 | 0 | 1 | 1 | | 1 | 1 | 1 | 0 | 1 | 1 | 1 | | 0 | 0 | 0 | 0 | 1 | | 1 | 1 | 1 | 1 | 1 | 0 | 0 | | 1 | 0 | | 0 | 0 | 0 | 1 | | 1 | 1 |
| 74 | Vision Rehabilitation Preferred Practice Pattern | 0 | 0 | 1 | 1 | 1 | 1 | 1 | 1 | | 1 | 0 | 1 | 0 | 1 | 1 | 0 | | 1 | 1 | 1 | 1 | 1 | | 1 | 1 | 0 | 1 | 1 | 0 | 1 | | 1 | 1 | | 1 | 1 | 1 | 1 | | 1 | 1 |
| 75 | Clinical Practice Guideline for Physical Therapist Management of People With Rheumatoid Arthritis | 1 | 0 | 1 | 1 | 1 | 1 | 1 | 1 | | 1 | 0 | 1 | 0 | 1 | 1 | 1 | | 1 | 1 | 1 | 1 | 1 | | 1 | 1 | 1 | 1 | 1 | 1 | 1 | | 1 | 1 | | 1 | 1 | 1 | 1 | | 1 | 1 |
| 76 | Mobilization and Exercise Intervention for Patients With Multiple Myeloma: Clinical Practice Guidelines Endorsed by the Canadian Physiotherapy Association | 1 | 0 | 1 | 0 | 1 | 1 | 1 | 1 | | 1 | 0 | 1 | 0 | 1 | 1 | 0 | | 0 | 1 | 1 | 1 | 1 | | 1 | 0 | 1 | 0 | 0 | 1 | 1 | | 1 | 1 | | 1 | 1 | 1 | 1 | | 1 | 1 |
| 77 | A Clinical Practice Guideline for the Use of Ankle-Foot Orthoses and Functional Electrical Stimulation Post-Stroke | 1 | 0 | 1 | 1 | 1 | 1 | 1 | 1 | | 1 | 0 | 1 | 0 | 1 | 1 | 1 | | 1 | 1 | 1 | 1 | 1 | | 1 | 1 | 1 | 1 | 1 | 1 | 1 | | 1 | 1 | | 1 | 1 | 1 | 1 | | 1 | 1 |
| 78 | Physical Therapy Management of Older Adults With Hip Fracture | 1 | 0 | 1 | 1 | 1 | 1 | 1 | 1 | | 1 | 0 | 1 | 0 | 1 | 1 | 1 | | 1 | 1 | 1 | 1 | 1 | | 1 | 1 | 0 | 1 | 0 | 0 | 1 | | 1 | 1 | | 1 | 1 | 0 | 1 | | 1 | 1 |
| 79 | KNGF Guideline on Low Back Pain and Lumbosacral Radicular Syndrome | 1 | 0 | 0 | 0 | 1 | 0 | 1 | 1 | | 1 | 1 | 1 | 0 | 1 | 1 | 1 | | 0 | 1 | 0 | 1 | 1 | | 1 | 1 | 1 | 1 | 1 | 0 | 0 | | 1 | 0 | | 0 | 0 | 0 | 0 | | 1 | 1 |
| 80 | The CanPain SCI clinical practice guidelines for rehabilitation management of neuropathic pain after spinal cord injury: 2021 update | 1 | 0 | 1 | 1 | 1 | 1 | 1 | 1 | | 1 | 0 | 1 | 0 | 1 | 1 | 0 | | 0 | 1 | 1 | 1 | 1 | | 1 | 1 | 1 | 0 | 1 | 1 | 1 | | 1 | 1 | | 0 | 1 | 0 | 1 | | 1 | 1 |
| 81 | Chinese Stroke Association guidelines for clinical management of cerebrovascular disorders: executive summary and 2019 update of clinical management of stroke rehabilitation | 1 | 1 | 1 | 0 | 1 | 1 | 1 | 1 | | 1 | 0 | 0 | 0 | 1 | 1 | 0 | | 0 | 0 | 0 | 1 | 1 | | 1 | 1 | 0 | 0 | 0 | 0 | 0 | | 0 | 1 | | 0 | 0 | 0 | 1 | | 0 | 0 |
| 82 | Physical Therapist Clinical Practice Guideline for the Management of Individuals With Heart Failure | 1 | 0 | 1 | 1 | 1 | 1 | 1 | 1 | | 1 | 0 | 1 | 0 | 1 | 1 | 1 | | 1 | 1 | 1 | 1 | 1 | | 1 | 1 | 1 | 1 | 1 | 1 | 1 | | 0 | 1 | | 0 | 1 | 0 | 1 | | 0 | 0 |
| 83 | Pulmonary rehabilitation principles in SARS-COV-2 infection (COVID-19): A guideline for the acute and subacute rehabilitation | 1 | 0 | 1 | 1 | 1 | 1 | 1 | 1 | | 1 | 1 | 1 | 1 | 0 | 1 | 0 | | 0 | 1 | 0 | 0 | 1 | | 1 | 0 | 0 | 0 | 0 | 1 | 0 | | 0 | 1 | | 1 | 1 | 0 | 0 | | 1 | 1 |
| 84 | Rehabilitation for adults with complex psychosis | 0 | 0 | 1 | 0 | 0 | 0 | 0 | 1 | | 1 | 0 | 1 | 0 | 1 | 1 | 1 | | 1 | 1 | 1 | 1 | 1 | | 1 | 1 | 1 | 1 | 1 | 1 | 1 | | 1 | 1 | | 0 | 1 | 1 | 1 | | 1 | 0 |
| 85 | Physical Therapy Evaluation and Treatment After Concussion/Mild Traumatic Brain Injury | 1 | 0 | 1 | 1 | 1 | 1 | 1 | 1 | | 1 | 0 | 1 | 0 | 1 | 1 | 1 | | 0 | 1 | 1 | 1 | 1 | | 1 | 1 | 0 | 0 | 0 | 0 | 1 | | 1 | 1 | | 1 | 1 | 1 | 1 | | 1 | 1 |
| 86 | Evidence-based national consensus: Recommendations for physiotherapy management in covid-19 in acute care indian setup | 1 | 0 | 1 | 0 | 1 | 1 | 1 | 1 | | 1 | 0 | 1 | 0 | 0 | 1 | 1 | | 0 | 1 | 1 | 1 | 1 | | 1 | 1 | 0 | 0 | 0 | 1 | 0 | | 1 | 1 | | 1 | 1 | 0 | 0 | | 1 | 1 |
| 87 | Clinical Practice Guideline to Improve Locomotor Function Following Chronic Stroke, Incomplete Spinal Cord Injury, and Brain Injury | 1 | 0 | 1 | 1 | 1 | 1 | 1 | 1 | | 1 | 1 | 1 | 0 | 0 | 1 | 1 | | 1 | 1 | 1 | 1 | 1 | | 1 | 1 | 0 | 1 | 1 | 1 | 1 | | 1 | 1 | | 1 | 1 | 0 | 1 | | 1 | 1 |
| 88 | A clinical practice guideline for physical therapy in patients with hip or knee osteoarthritis | 1 | 0 | 1 | 1 | 1 | 1 | 1 | 1 | | 1 | 0 | 0 | 0 | 1 | 1 | 1 | | 0 | 1 | 1 | 1 | 1 | | 1 | 0 | 1 | 0 | 1 | 1 | 1 | | 0 | 1 | | 0 | 1 | 0 | 1 | | 1 | 1 |
| 89 | The Turkish Society of Physical Medicine and Rehabilitation (TSPMR) guideline recommendations for the management of fibromyalgia syndrome | 1 | 0 | 1 | 1 | 1 | 1 | 1 | 1 | | 1 | 0 | 1 | 0 | 0 | 1 | 0 | | 0 | 1 | 1 | 1 | 1 | | 1 | 1 | 0 | 0 | 0 | 1 | 0 | | 0 | 1 | | 1 | 1 | 0 | 0 | | 1 | 0 |
| 90 | The management of stroke rehabilitation: A synopsis of the 2019 U.S. Department of Veterans Affairs and U.S. Department of Defense clinical practice guideline | 1 | 1 | 1 | 1 | 1 | 1 | 1 | 1 | | 1 | 0 | 1 | 0 | 1 | 1 | 1 | | 0 | 1 | 1 | 1 | 1 | | 1 | 1 | 0 | 0 | 0 | 0 | 0 | | 1 | 1 | | 0 | 1 | 1 | 1 | | 0 | 0 |
| 91 | Clinical Practice Guidelines for the Rehabilitation of Lower Limb Amputation: An Update from the Department of Veterans Affairs and Department of Defense | 1 | 0 | 1 | 1 | 1 | 1 | 1 | 1 | | 1 | 0 | 1 | 0 | 0 | 0 | 1 | | 0 | 1 | 1 | 1 | 1 | | 1 | 1 | 1 | 0 | 0 | 1 | 1 | | 1 | 1 | | 0 | 1 | 1 | 1 | | 1 | 1 |
| 92 | Clinical practice guideline for cardiac rehabilitation in Korea: recommendations for cardiac rehabilitation and secondary prevention after acute coronary syndrome | 1 | 0 | 1 | 1 | 1 | 1 | 1 | 1 | | 1 | 0 | 1 | 0 | 1 | 1 | 1 | | 0 | 1 | 1 | 1 | 1 | | 1 | 1 | 0 | 0 | 0 | 1 | 1 | | 1 | 1 | | 1 | 1 | 0 | 1 | | 1 | 1 |
| 93 | The Ottawa Panel guidelines on programmes involving therapeutic exercise for the management of hand osteoarthritis | 1 | 0 | 1 | 0 | 1 | 1 | 1 | 1 | | 1 | 0 | 1 | 0 | 0 | 1 | 1 | | 1 | 1 | 1 | 1 | 1 | | 0 | 0 | 0 | 0 | 0 | 1 | 0 | | 0 | 1 | | 0 | 1 | 0 | 0 | | 1 | 0 |
| 94 | Spinal Manipulative Therapy and Other Conservative Treatments for Low Back Pain: A Guideline From the Canadian Chiropractic Guideline Initiative | 1 | 0 | 1 | 0 | 1 | 1 | 1 | 1 | | 1 | 1 | 1 | 0 | 1 | 1 | 1 | | 1 | 1 | 1 | 1 | 1 | | 1 | 1 | 1 | 1 | 0 | 1 | 1 | | 1 | 1 | | 1 | 1 | 0 | 1 | | 1 | 1 |
| 95 | Evidence- and Consensus-Based Guidelines for the Management of Communication and Swallowing Disorders Following Pediatric Traumatic Brain Injury | 1 | 0 | 1 | 1 | 1 | 1 | 1 | 1 | | 1 | 0 | 1 | 0 | 1 | 1 | 1 | | 1 | 1 | 1 | 1 | 1 | | 1 | 1 | 0 | 0 | 0 | 1 | 0 | | 1 | 1 | | 0 | 1 | 0 | 1 | | 1 | 1 |
| 96 | Unique features of the INESSS-Onf rehabilitation guidelines for moderate to severe traumatic brain injury: Responding to users' needs | 1 | 0 | 1 | 1 | 1 | 1 | 1 | 1 | | 1 | 0 | 1 | 0 | 0 | 1 | 1 | | 0 | 1 | 1 | 1 | 1 | | 1 | 1 | 1 | 0 | 0 | 1 | 1 | | 0 | 1 | | 0 | 1 | 0 | 1 | | 1 | 1 |
| 97 | A Core Set of Outcome Measures for Adults With Neurologic Conditions Undergoing Rehabilitation: A CLINICAL PRACTICE GUIDELINE | 1 | 0 | 1 | 1 | 1 | 1 | 1 | 1 | | 1 | 0 | 1 | 0 | 1 | 1 | 1 | | 1 | 1 | 1 | 1 | 1 | | 1 | 1 | 1 | 1 | 1 | 1 | 1 | | 1 | 1 | | 1 | 1 | 0 | 1 | | 1 | 1 |
| 98 | KNGF guideline Rheumatoid arthritis | 1 | 0 | 0 | 0 | 0 | 0 | 1 | 1 | | 1 | 0 | 1 | 0 | 1 | 1 | 1 | | 0 | 0 | 0 | 0 | 1 | | 1 | 0 | 0 | 1 | 1 | 0 | 1 | | 1 | 0 | | 0 | 0 | 0 | 0 | | 0 | 0 |
| 99 | KNGF guideline Osteoarthritis of the hip-knee | 1 | 0 | 1 | 0 | 0 | 0 | 1 | 1 | | 1 | 1 | 1 | 0 | 1 | 1 | 1 | | 0 | 0 | 0 | 0 | 1 | | 1 | 0 | 0 | 1 | 1 | 0 | 1 | | 1 | 0 | | 0 | 0 | 0 | 0 | | 1 | 1 |
| 100 | 2016 SOSORT guidelines: orthopaedic and rehabilitation treatment of idiopathic scoliosis during growth | 1 | 1 | 1 | 1 | 1 | 1 | 1 | 1 | | 1 | 0 | 1 | 0 | 0 | 1 | 1 | | 0 | 1 | 1 | 1 | 1 | | 1 | 1 | 1 | 1 | 0 | 1 | 1 | | 1 | 1 | | 1 | 1 | 1 | 1 | | 1 | 0 |
| 101 | Evidence-based (GRADE approach) Korean medicine clinical practice guidelines of manual acupuncture for the treatment of shoulder pain | 1 | 0 | 1 | 0 | 1 | 1 | 1 | 1 | | 1 | 0 | 1 | 0 | 0 | 1 | 1 | | 1 | 1 | 1 | 1 | 1 | | 1 | 1 | 1 | 1 | 0 | 1 | 0 | | 1 | 1 | | 1 | 1 | 0 | 1 | | 1 | 1 |
| 102 | Clinical practice guidelines of acupuncture-moxibustion for cervical spondylotic radiculopath | 1 | 0 | 1 | 0 | 1 | 1 | 1 | 1 | | 0 | 0 | 1 | 0 | 0 | 1 | 1 | | 0 | 1 | 1 | 1 | 1 | | 1 | 1 | 1 | 1 | 0 | 0 | 1 | | 1 | 1 | | 0 | 1 | 0 | 0 | | 0 | 1 |
| 103 | A Clinical Practice Guideline for the Management of Patients With Acute Spinal Cord Injury: Recommendations on the Type and Timing of Rehabilitation | 1 | 0 | 1 | 1 | 1 | 1 | 1 | 1 | | 1 | 0 | 1 | 0 | 0 | 1 | 1 | | 1 | 1 | 0 | 1 | 1 | | 1 | 1 | 1 | 1 | 0 | 0 | 1 | | 1 | 1 | | 1 | 1 | 0 | 0 | | 0 | 0 |
| 104 | Cardiac Rehabilitation for Patients With Coronary Artery Disease: A Practical Guide to Enhance Patient Outcomes Through Continuity of Care | 1 | 0 | 1 | 0 | 1 | 1 | 1 | 1 | | 1 | 0 | 1 | 0 | 0 | 1 | 0 | | 0 | 0 | 0 | 0 | 1 | | 0 | 0 | 0 | 0 | 0 | 0 | 0 | | 0 | 1 | | 0 | 1 | 0 | 0 | | 0 | 1 |
| 105 | The Ottawa panel clinical practice guidelines for the management of knee osteoarthritis. Part two: strengthening exercise programs | 1 | 0 | 1 | 1 | 1 | 1 | 0 | 1 | | 1 | 0 | 1 | 0 | 0 | 1 | 1 | | 1 | 1 | 1 | 1 | 1 | | 1 | 1 | 1 | 0 | 0 | 1 | 0 | | 0 | 1 | | 0 | 1 | 0 | 1 | | 0 | 1 |
| 106 | Cardiac rehabilitation | 0 | 0 | 1 | 1 | 1 | 1 | 1 | 1 | | 1 | 1 | 1 | 0 | 1 | 1 | 0 | | 0 | 1 | 1 | 1 | 1 | | 1 | 1 | 1 | 1 | 0 | 0 | 1 | | 1 | 1 | | 0 | 1 | 1 | 1 | | 0 | 1 |
| 107 | Australian and New Zealand Pulmonary Rehabilitation Guidelines | 1 | 0 | 1 | 1 | 1 | 1 | 1 | 1 | | 1 | 1 | 1 | 0 | 0 | 1 | 1 | | 1 | 1 | 1 | 1 | 1 | | 1 | 1 | 1 | 1 | 1 | 1 | 0 | | 1 | 1 | | 1 | 1 | 1 | 1 | | 0 | 0 |
| 108 | General guidelines in the rehabilitation process for return to training after a sports injury | 1 | 0 | 1 | 0 | 1 | 1 | 1 | 1 | | 1 | 0 | 1 | 0 | 0 | 1 | 1 | | 0 | 1 | 0 | 0 | 0 | | 0 | 0 | 0 | 0 | 0 | 0 | 0 | | 1 | 0 | | 0 | 1 | 0 | 0 | | 1 | 1 |
| 109 | The CanPain SCI Clinical Practice Guidelines for Rehabilitation Management of Neuropathic Pain after Spinal Cord: introduction, methodology and recommendation overview | 1 | 0 | 1 | 1 | 1 | 1 | 1 | 1 | | 1 | 0 | 1 | 0 | 1 | 1 | 1 | | 1 | 1 | 1 | 1 | 1 | | 1 | 1 | 0 | 1 | 0 | 1 | 1 | | 1 | 1 | | 1 | 1 | 0 | 1 | | 1 | 1 |
| 110 | INESSS-ONF clinical practice guidelines for the rehabilitation of adults having sustained a moderate-to-severe TBI | 1 | 0 | 1 | 1 | 1 | 0 | 0 | 0 | | 1 | 0 | 1 | 0 | 0 | 0 | 1 | | 0 | 0 | 0 | 1 | 1 | | 1 | 1 | 1 | 1 | 0 | 0 | 0 | | 0 | 0 | | 0 | 0 | 0 | 1 | | 0 | 0 |
| 111 | Sexual Rehabilitation After Treatment for Prostate Cancer—Part 1: Recommendations From the Fourth International Consultation for Sexual Medicine (ICSM 2015) | 0 | 1 | 1 | 1 | 1 | 1 | 1 | 1 | | 1 | 0 | 0 | 0 | 0 | 1 | 0 | | 0 | 1 | 0 | 1 | 1 | | 1 | 1 | 0 | 0 | 0 | 1 | 0 | | 1 | 1 | | 1 | 1 | 0 | 0 | | 0 | 1 |
| 112 | Telerehabilitation in Physical Therapist Practice: A Clinical Practice Guideline From the American Physical Therapy Association | 1 | 0 | 1 | 1 | 1 | 1 | 1 | 1 | | 1 | 0 | 1 | 0 | 1 | 1 | 1 | | 1 | 1 | 1 | 1 | 1 | | 0 | 1 | 1 | 1 | 1 | 1 | 1 | | 1 | 1 | | 1 | 1 | 1 | 1 | | 0 | 1 |
| 113 | Digital technologies to deliver pulmonary rehabilitation programmes for adults with COPD: early value assessment | 1 | 0 | 1 | 0 | 0 | 1 | 0 | 1 | | 0 | 0 | 0 | 0 | 0 | 0 | 1 | | 1 | 1 | 0 | 0 | 1 | | 0 | 0 | 1 | 1 | 0 | 1 | 1 | | 1 | 1 | | 0 | 1 | 0 | 1 | | 0 | 0 |
| 114 | GaitSmart rehabilitation exercise programme for gait and mobility issues | 1 | 0 | 1 | 1 | 1 | 0 | 1 | 1 | | 1 | 1 | 0 | 0 | 1 | 0 | 0 | | 0 | 1 | 0 | 0 | 0 | | 0 | 1 | 1 | 1 | 1 | 1 | 1 | | 1 | 1 | | 0 | 1 | 0 | 1 | | 1 | 1 |
| 115 | VA/DoD CLINICAL PRACTICE GUIDELINE FOR MANAGEMENT OF STROKE REHABILITATION | 1 | 0 | 1 | 1 | 1 | 1 | 1 | 1 | | 1 | 0 | 1 | 0 | 1 | 1 | 1 | | 1 | 1 | 1 | 1 | 1 | | 1 | 1 | 1 | 1 | 1 | 1 | 1 | | 1 | 1 | | 1 | 1 | 1 | 1 | | 1 | 1 |
| 116 | 2022 American College of Rheumatology Guideline for Exercise, Rehabilitation, Diet, and Additional Integrative Interventions for Rheumatoid Arthritis | 1 | 1 | 1 | 1 | 0 | 1 | 1 | 1 | | 1 | 0 | 0 | 0 | 1 | 1 | 1 | | 1 | 0 | 1 | 1 | 1 | | 0 | 1 | 1 | 1 | 0 | 0 | 0 | | 1 | 0 | | 0 | 1 | 1 | 1 | | 1 | 1 |
| 117 | Role of Physical Therapists in the Management of Individuals at Risk for or Diagnosed With Venous Thromboembolism: Evidence-Based Clinical Practice Guideline 2022 | 1 | 1 | 1 | 1 | 1 | 1 | 1 | 1 | | 1 | 0 | 1 | 0 | 1 | 1 | 1 | | 1 | 1 | 1 | 1 | 1 | | 0 | 1 | 1 | 1 | 1 | 1 | 1 | | 1 | 1 | | 1 | 1 | 1 | 1 | | 1 | 1 |
| 118 | Canadian Stroke Best Practice Recommendations Telestroke Best Practice Guidelines Update 2017 | 1 | 1 | 1 | 1 | 0 | 1 | 1 | 0 | | 1 | 0 | 1 | 0 | 1 | 1 | 0 | | 0 | 1 | 1 | 1 | 1 | | 0 | 1 | 0 | 1 | 0 | 1 | 1 | | 1 | 1 | | 1 | 1 | 1 | 1 | | 0 | 0 |
| 119 | Ottawa Panel Evidence-Based Clinical Practice Guidelines for Structured Physical Activity in the Management of Juvenile Idiopathic Arthritis | 1 | 0 | 1 | 1 | 1 | 1 | 1 | 1 | | 1 | 0 | 1 | 0 | 1 | 1 | 1 | | 1 | 1 | 1 | 1 | 1 | | 0 | 1 | 1 | 0 | 1 | 1 | 1 | | 0 | 1 | | 1 | 1 | 1 | 1 | | 1 | 1 |
| 120 | Clinical Practice Guideline for Physical Therapy Assessment and Treatment in Patients With Nonspecific Neck Pain | 1 | 0 | 1 | 0 | 0 | 1 | 1 | 1 | | 1 | 0 | 1 | 0 | 1 | 1 | 1 | | 1 | 1 | 0 | 1 | 1 | | 0 | 1 | 1 | 1 | 1 | 1 | 1 | | 1 | 1 | | 0 | 1 | 1 | 1 | | 1 | 1 |
| 121 | VA/DoD CLINICAL PRACTICE GUIDELINE FOR REHABILITATION OF INDIVIDUALS WITH LOWER LIMB AMPUTATION | 1 | 0 | 1 | 1 | 1 | 1 | 1 | 1 | | 1 | 1 | 1 | 0 | 1 | 1 | 1 | | 1 | 1 | 1 | 1 | 1 | | 1 | 1 | 1 | 1 | 1 | 1 | 1 | | 1 | 1 | | 0 | 1 | 1 | 1 | | 1 | 1 |
| 122 | VA/DoD CLINICAL PRACTICE GUIDELINE FOR THE MANAGEMENT OF UPPER LIMB AMPUTATION REHABILITATION | 1 | 0 | 1 | 1 | 1 | 1 | 1 | 1 | | 1 | 0 | 1 | 0 | 1 | 1 | 1 | | 1 | 1 | 1 | 1 | 1 | | 0 | 1 | 1 | 1 | 1 | 1 | 1 | | 1 | 1 | | 1 | 1 | 1 | 1 | | 1 | 1 |
| 123 | ISPAD Clinical Practice Consensus Guidelines 2022: Exercise in children and adolescents with diabetes | 1 | 1 | 1 | 1 | 1 | 1 | 1 | 1 | | 1 | 0 | 1 | 0 | 1 | 1 | 1 | | 1 | 1 | 1 | 1 | 1 | | 0 | 1 | 1 | 1 | 1 | 1 | 1 | | 1 | 1 | | 1 | 1 | 1 | 1 | | 1 | 1 |
| 124 | Exercise-based knee and anterior cruciate ligament injury prevention.2022 | 1 | 0 | 1 | 1 | 1 | 1 | 1 | 1 | | 1 | 0 | 1 | 1 | 1 | 1 | 1 | | 1 | 1 | 1 | 1 | 1 | | 0 | 1 | 1 | 1 | 1 | 1 | 1 | | 1 | 1 | | 0 | 1 | 0 | 1 | | 1 | 1 |
| 125 | VA/DoD CLINICAL PRACTICE GUIDELINE FOR THE MANAGEMENT AND REHABILITATION OF POST-ACUTE MILD TRAUMATIC BRAIN INJURY | 1 | 0 | 1 | 1 | 1 | 1 | 1 | 1 | | 1 | 0 | 1 | 0 | 1 | 1 | 1 | | 1 | 1 | 1 | 1 | 1 | | 1 | 1 | 1 | 1 | 1 | 1 | 1 | | 1 | 1 | | 1 | 1 | 1 | 1 | | 1 | 1 |
| 126 | JCS/JACR 2021 Guideline on Rehabilitation in Patients With Cardiovascular Disease | 1 | 1 | 1 | 1 | 1 | 1 | 1 | 1 | | 1 | 1 | 1 | 0 | 1 | 1 | 1 | | 1 | 1 | 1 | 1 | 1 | | 1 | 0 | 1 | 1 | 1 | 1 | 1 | | 1 | 1 | | 1 | 1 | 1 | 1 | | 1 | 1 |
| 127 | Canadian Stroke Best Practice Recommendations: Rehabilitation, Recovery, and Community Participation following Stroke | 1 | 1 | 1 | 0 | 0 | 1 | 1 | 1 | | 1 | 1 | 1 | 0 | 1 | 1 | 0 | | 0 | 1 | 0 | 1 | 1 | | 1 | 1 | 1 | 1 | 1 | 1 | 1 | | 1 | 1 | | 0 | 1 | 0 | 1 | | 1 | 0 |
| 128 | VA/DOD Clinical practice guideline for the management of stroke rehabilitation | 1 | 0 | 1 | 1 | 1 | 1 | 1 | 1 | | 1 | 0 | 1 | 0 | 1 | 1 | 1 | | 1 | 1 | 1 | 1 | 1 | | 0 | 1 | 1 | 1 | 1 | 1 | 1 | | 1 | 1 | | 1 | 1 | 1 | 1 | | 1 | 1 |
| 129 | Occupational therapy for epidermolysis bullosa: Clinical practice guidelines | 1 | 0 | 1 | 1 | 1 | 1 | 1 | 1 | | 1 | 0 | 1 | 0 | 1 | 1 | 1 | | 1 | 1 | 1 | 1 | 1 | | 1 | 0 | 1 | 0 | 0 | 1 | 0 | | 1 | 1 | | 1 | 1 | 1 | 0 | | 1 | 1 |
| 130 | Vision Rehabilitation Preferred Practice Pattern | 1 | 0 | 1 | 1 | 0 | 1 | 1 | 1 | | 1 | 0 | 1 | 0 | 1 | 1 | 1 | | 1 | 1 | 1 | 1 | 1 | | 1 | 1 | 0 | 1 | 1 | 1 | 1 | | 1 | 1 | | 1 | 1 | 1 | 1 | | 1 | 1 |
| 131 | ISPAD Clinical Practice Consensus Guidelines 2018: Exercise in children and adolescents with diabetes | 1 | 1 | 1 | 1 | 1 | 1 | 1 | 1 | | 1 | 0 | 0 | 0 | 1 | 0 | 0 | | 0 | 0 | 0 | 0 | 1 | | 1 | 0 | 0 | 0 | 1 | 0 | 0 | | 1 | 0 | | 0 | 0 | 0 | 0 | | 1 | 0 |
| 132 | Exercise-based knee and anterior cruciate ligament injury prevention 2018 | 1 | 1 | 1 | 1 | 0 | 1 | 1 | 1 | | 1 | 0 | 1 | 1 | 0 | 1 | 1 | | 0 | 1 | 1 | 1 | 0 | | 0 | 1 | 1 | 1 | 1 | 1 | 1 | | 0 | 1 | | 1 | 1 | 1 | 0 | | 0 | 0 |
| 133 | Canadian stroke best practice recommendations: Stroke rehabilitation practice guidelines, update 2015 of stroke, sixth edition practice guidelines, update 2017 | 1 | 1 | 1 | 1 | 0 | 1 | 1 | 1 | | 1 | 0 | 1 | 0 | 1 | 0 | 1 | | 1 | 1 | 1 | 1 | 1 | | 0 | 1 | 1 | 0 | 0 | 1 | 1 | | 1 | 1 | | 1 | 1 | 1 | 1 | | 1 | 0 |
| 134 | Vestibular rehabilitation for peripheral vestibular hypofunction: an evidence-based clinical practice guideline: from the American Physical Therapy Associa tion Neurology Section | 1 | 0 | 1 | 1 | 1 | 1 | 1 | 1 | | 1 | 0 | 1 | 0 | 1 | 1 | 1 | | 1 | 1 | 1 | 1 | 1 | | 1 | 1 | 1 | 1 | 1 | 1 | 1 | | 1 | 1 | | 1 | 1 | 1 | 1 | | 1 | 1 |
| 135 | Role of Physical Therapists in the Management of Individuals at Risk for or Diagnosed With Venous Thromboembolism: Evidence-Based Clinical Practice Guideline | 1 | 0 | 1 | 1 | 0 | 1 | 1 | 1 | | 1 | 0 | 1 | 0 | 1 | 1 | 1 | | 0 | 1 | 1 | 0 | 1 | | 1 | 1 | 1 | 1 | 1 | 1 | 1 | | 1 | 1 | | 1 | 1 | 1 | 1 | | 1 | 1 |
| 136 | VA/DoD CLINICAL PRACTICE GUIDELINE FOR THE MANAGEMENT OF CONCUSSION-MILD TRAUMATIC BRAIN INJURY | 1 | 0 | 1 | 1 | 1 | 1 | 1 | 1 | | 1 | 0 | 1 | 0 | 1 | 1 | 1 | | 1 | 1 | 1 | 1 | 1 | | 0 | 1 | 1 | 1 | 1 | 1 | 1 | | 1 | 1 | | 1 | 1 | 1 | 1 | | 1 | 1 |
| 137 | The CanPain SCI Clinical Practice Guideline for Rehabilitation Management of Neuropathic Pain after Spinal Cord: Recommendations for model systems of care | 1 | 0 | 1 | 0 | 0 | 1 | 1 | 1 | | 1 | 0 | 1 | 0 | 0 | 0 | 1 | | 0 | 0 | 1 | 0 | 1 | | 0 | 0 | 1 | 0 | 1 | 1 | 0 | | 1 | 1 | | 1 | 1 | 1 | 0 | | 0 | 0 |
| 138 | Speech and swallow rehabilitation in head and neck cancer: United Kingdom National Multidisciplinary Guidelines | 1 | 0 | 1 | 1 | 0 | 1 | 1 | 1 | | 1 | 0 | 0 | 0 | 1 | 0 | 0 | | 0 | 0 | 0 | 0 | 1 | | 1 | 0 | 0 | 0 | 1 | 0 | 0 | | 1 | 0 | | 0 | 0 | 0 | 0 | | 0 | 0 |
| 139 | Vestibular Rehabilitation for Peripheral Vestibular Hypofunction: An Evidence-Based Clinical Practice Guideline: FROM THE AMERICAN PHYSICAL THERAPY ASSOCIATION NEUROLOGY SECTION | 1 | 0 | 1 | 0 | 0 | 0 | 1 | 1 | | 1 | 0 | 0 | 0 | 0 | 1 | 0 | | 0 | 0 | 0 | 0 | 0 | | 0 | 0 | 0 | 0 | 0 | 0 | 0 | | 0 | 0 | | 0 | 0 | 0 | 0 | | 1 | 1 |
| 140 | Canadian stroke best practice recommendations: Stroke rehabilitation practice guidelines, update 2015 | 1 | 1 | 1 | 0 | 1 | 1 | 1 | 1 | | 1 | 0 | 1 | 0 | 1 | 1 | 0 | | 0 | 1 | 1 | 1 | 1 | | 0 | 1 | 0 | 0 | 0 | 1 | 1 | | 1 | 1 | | 0 | 1 | 0 | 0 | | 0 | 0 |
| 141 | Guidelines for Adult Stroke Rehabilitation and Recovery: A Guideline for Healthcare Professionals from the American Heart Association/American Stroke Association | 1 | 0 | 1 | 0 | 1 | 0 | 1 | 1 | | 1 | 0 | 0 | 0 | 1 | 1 | 0 | | 0 | 0 | 0 | 1 | 1 | | 0 | 1 | 0 | 0 | 0 | 0 | 1 | | 0 | 0 | | 0 | 0 | 1 | 0 | | 1 | 1 |
| 142 | KNGF Clinical Practice Guideline for Physical Therapy in patients with neck pain | 1 | 0 | 1 | 0 | 1 | 0 | 1 | 1 | | 1 | 0 | 1 | 0 | 0 | 0 | 0 | | 0 | 0 | 0 | 0 | 1 | | 0 | 0 | 0 | 0 | 0 | 0 | 1 | | 0 | 0 | | 0 | 0 | 0 | 1 | | 0 | 0 |
| 143 | The CanPain SCI Clinical Practice Guidelines for Rehabilitation Management of Neuropathic Pain after Spinal Cord: Recommendations for treatment | 1 | 0 | 1 | 0 | 1 | 1 | 1 | 1 | | 1 | 0 | 0 | 0 | 0 | 1 | 0 | | 0 | 0 | 0 | 1 | 1 | | 0 | 1 | 0 | 0 | 0 | 1 | 1 | | 1 | 1 | | 0 | 1 | 0 | 0 | | 1 | 1 |
| 144 | Evidence-based clinical practice update: practice guidelines for anterior cruciate ligament rehabilitation based on a systematic review and multidisciplinary consensus | 1 | 0 | 1 | 1 | 1 | 1 | 1 | 1 | | 1 | 0 | 0 | 0 | 0 | 1 | 1 | | 1 | 1 | 1 | 1 | 1 | | 0 | 1 | 0 | 0 | 0 | 1 | 1 | | 1 | 0 | | 0 | 1 | 0 | 0 | | 1 | 1 |
| 145 | The CanPain SCI Clinical Practice Guidelines for Rehabilitation Management of Neuropathic Pain after Spinal Cord: screening and diagnosis recommendations | 1 | 0 | 1 | 0 | 1 | 1 | 1 | 1 | | 1 | 0 | 1 | 0 | 0 | 1 | 0 | | 0 | 0 | 0 | 1 | 1 | | 0 | 1 | 0 | 0 | 0 | 0 | 0 | | 1 | 1 | | 0 | 1 | 0 | 0 | | 1 | 1 |
| 146 | Practice guidelines for program evaluation in community-based rehabilitation | 1 | 0 | 1 | 1 | 1 | 1 | 1 | 1 | | 1 | 0 | 0 | 0 | 0 | 1 | 0 | | 0 | 1 | 1 | 0 | 1 | | 0 | 0 | 0 | 0 | 0 | 0 | 1 | | 1 | 1 | | 0 | 0 | 0 | 1 | | 0 | 0 |
| 147 | Stroke rehabilitation: long-term rehabilitation after stroke | 0 | 0 | 1 | 0 | 1 | 0 | 1 | 1 | | 1 | 0 | 1 | 0 | 1 | 0 | 0 | | 0 | 0 | 0 | 0 | 1 | | 0 | 0 | 0 | 0 | 0 | 1 | 0 | | 0 | 0 | | 0 | 0 | 0 | 1 | | 1 | 0 |
| 148 | Ottawa Panel evidence-based clinical practice guidelines for therapeutic exercise in the management of hip osteoarthritis | 1 | 0 | 1 | 0 | 1 | 1 | 1 | 1 | | 1 | 0 | 1 | 0 | 0 | 1 | 0 | | 0 | 1 | 1 | 1 | 1 | | 0 | 1 | 0 | 0 | 0 | 1 | 0 | | 1 | 1 | | 0 | 1 | 0 | 1 | | 0 | 1 |
| 149 | Practice guidelines for cardiovascular fitness and strengthening exercise prescription after burn injury | 1 | 0 | 0 | 1 | 1 | 1 | 1 | 1 | | 1 | 0 | 0 | 1 | 0 | 1 | 0 | | 0 | 1 | 1 | 1 | 1 | | 0 | 0 | 0 | 0 | 0 | 0 | 1 | | 0 | 1 | | 0 | 0 | 0 | 0 | | 1 | 1 |
| 150 | Exercise and Habitual Physical Activity for People with Cystic Fibrosis: Expert Consensus, Evidence-Based Guide for Advising Patients | 1 | 0 | 0 | 0 | 1 | 1 | 1 | 1 | | 1 | 0 | 0 | 0 | 0 | 1 | 1 | | 0 | 0 | 0 | 0 | 1 | | 1 | 0 | 0 | 0 | 0 | 0 | 1 | | 0 | 1 | | 0 | 1 | 0 | 0 | | 0 | 0 |
| 151 | Exercise-based cardiac rehabilitation in patients with chronic heart failure: a Dutch practice guideline | 1 | 0 | 1 | 0 | 1 | 1 | 1 | 1 | | 1 | 0 | 0 | 0 | 0 | 1 | 0 | | 0 | 1 | 1 | 1 | 1 | | 0 | 1 | 0 | 0 | 0 | 0 | 0 | | 1 | 1 | | 0 | 1 | 0 | 0 | | 0 | 1 |
| 152 | VA/DoD CLINICAL PRACTICE GUIDELINE FOR THE MANAGEMENT OF UPPER LIMB AMPUTATION REHABILITATION | 1 | 0 | 1 | 1 | 1 | 0 | 1 | 1 | | 1 | 0 | 0 | 0 | 0 | 1 | 1 | | 1 | 1 | 1 | 1 | 1 | | 0 | 1 | 0 | 0 | 0 | 0 | 0 | | 1 | 0 | | 0 | 0 | 0 | 0 | | 1 | 0 |
| 153 | ISPAD Clinical Practice Consensus Guidelines 2014. Exercise in children and adolescents with diabetes | 1 | 1 | 0 | 0 | 1 | 1 | 1 | 1 | | 1 | 0 | 0 | 1 | 0 | 1 | 0 | | 0 | 1 | 0 | 0 | 1 | | 1 | 0 | 0 | 0 | 1 | 0 | 1 | | 0 | 1 | | 0 | 1 | 0 | 0 | | 0 | 1 |
| 154 | Clinical Competency Guidelines for Pulmonary Rehabilitation Professionals 2014 | 1 | 1 | 1 | 1 | 1 | 0 | 1 | 1 | | 1 | 0 | 1 | 0 | 0 | 1 | 0 | | 0 | 1 | 0 | 0 | 1 | | 0 | 0 | 0 | 0 | 0 | 0 | 1 | | 1 | 0 | | 0 | 0 | 0 | 0 | | 1 | 1 |
| 155 | Guidelines for rehabilitation in patients with cardiovascular disease (JCS 2012) | 1 | 1 | 1 | 0 | 1 | 1 | 1 | 1 | | 1 | 0 | 0 | 0 | 0 | 1 | 0 | | 0 | 1 | 0 | 1 | 1 | | 1 | 1 | 0 | 0 | 0 | 0 | 0 | | 1 | 0 | | 0 | 0 | 0 | 0 | | 1 | 1 |
| 156 | Physical activity exercise referral schemes | 1 | 0 | 1 | 0 | 1 | 0 | 1 | 1 | | 1 | 1 | 0 | 0 | 1 | 1 | 1 | | 0 | 1 | 1 | 1 | 1 | | 1 | 0 | 0 | 0 | 0 | 1 | 0 | | 1 | 0 | | 0 | 0 | 0 | 1 | | 1 | 1 |
| 157 | KNGF Clinical Practice Guideline for Physical Therapy in patients with stroke | 1 | 0 | 1 | 0 | 1 | 0 | 1 | 1 | | 1 | 0 | 0 | 1 | 0 | 0 | 0 | | 0 | 0 | 0 | 0 | 1 | | 0 | 0 | 0 | 0 | 0 | 0 | 0 | | 0 | 0 | | 0 | 0 | 0 | 1 | | 0 | 0 |
| 158 | Rehabilitation guidelines after rotator cuff repair | 1 | 0 | 1 | 0 | 1 | 1 | 1 | 1 | | 1 | 0 | 0 | 0 | 0 | 1 | 0 | | 0 | 0 | 0 | 0 | 1 | | 0 | 0 | 0 | 0 | 0 | 0 | 0 | | 0 | 0 | | 0 | 1 | 0 | 0 | | 1 | 1 |
| 159 | The 2013 Canadian Cardiovascular Society Heart Failure Management Guidelines Update: Focus on Rehabilitation and Exercise and Surgical Coronary Revascularization | 1 | 1 | 1 | 0 | 1 | 1 | 1 | 1 | | 1 | 1 | 0 | 0 | 0 | 1 | 0 | | 0 | 0 | 0 | 0 | 1 | | 1 | 0 | 0 | 0 | 0 | 0 | 0 | | 0 | 1 | | 0 | 0 | 0 | 1 | | 0 | 1 |
| 160 | Brain injury rehabilitation in adults | 1 | 0 | 1 | 0 | 1 | 0 | 1 | 1 | | 1 | 1 | 0 | 0 | 1 | 1 | 1 | | 0 | 1 | 1 | 1 | 1 | | 1 | 0 | 0 | 0 | 0 | 1 | 1 | | 1 | 0 | | 0 | 0 | 0 | 1 | | 1 | 1 |
| 161 | British Thoracic Society guideline on pulmonary rehabilitation in adults | 1 | 0 | 1 | 0 | 1 | 1 | 1 | 1 | | 1 | 1 | 0 | 1 | 0 | 1 | 0 | | 0 | 1 | 1 | 1 | 1 | | 1 | 0 | 1 | 0 | 0 | 1 | 1 | | 1 | 1 | | 0 | 0 | 0 | 1 | | 0 | 0 |
| 162 | Exercise-based cardiac rehabilitation in patients with chronic heart failure: a Dutch practice guideline | 1 | 0 | 1 | 0 | 1 | 1 | 1 | 1 | | 1 | 0 | 0 | 1 | 0 | 1 | 0 | | 0 | 1 | 1 | 1 | 1 | | 1 | 0 | 0 | 0 | 0 | 1 | 1 | | 0 | 1 | | 0 | 1 | 0 | 1 | | 1 | 0 |
| 163 | Ottawa panel evidence-based clinical practice guidelines for aerobic walking programs in the management of osteoarthritis | 1 | 0 | 1 | 0 | 1 | 1 | 1 | 1 | | 1 | 0 | 1 | 0 | 0 | 1 | 1 | | 1 | 1 | 1 | 1 | 1 | | 0 | 0 | 1 | 0 | 0 | 1 | 1 | | 1 | 0 | | 0 | 0 | 0 | 0 | | 0 | 1 |
| 164 | Ottawa Panel evidence-based clinical practice guidelines on therapeutic massage for neck pain | 1 | 0 | 1 | 0 | 1 | 1 | 1 | 1 | | 1 | 0 | 0 | 0 | 1 | 1 | 1 | | 1 | 1 | 1 | 1 | 1 | | 0 | 1 | 0 | 0 | 0 | 1 | 1 | | 1 | 1 | | 0 | 0 | 0 | 0 | | 1 | 1 |
| 165 | Ottawa Panel evidence-based clinical practice guidelines on therapeutic massage for low back pain | 1 | 0 | 1 | 0 | 1 | 1 | 1 | 1 | | 1 | 0 | 0 | 1 | 1 | 1 | 1 | | 1 | 1 | 1 | 1 | 1 | | 0 | 0 | 0 | 0 | 0 | 1 | 1 | | 1 | 1 | | 0 | 0 | 0 | 0 | | 0 | 1 |
| 166 | Expert opinion and key recommendations for the physical therapy and rehabilitation of patients with ankylosing spondylitis | 1 | 0 | 1 | 1 | 1 | 1 | 1 | 1 | | 1 | 0 | 0 | 0 | 0 | 1 | 0 | | 0 | 1 | 1 | 1 | 1 | | 0 | 0 | 0 | 0 | 0 | 1 | 0 | | 1 | 0 | | 0 | 0 | 0 | 0 | | 1 | 1 |
| 167 | French Society of Cardiology guidelines for cardiac rehabilitation in adults | 1 | 0 | 1 | 0 | 1 | 1 | 1 | 1 | | 1 | 0 | 0 | 0 | 0 | 1 | 0 | | 0 | 0 | 0 | 0 | 1 | | 1 | 1 | 0 | 0 | 0 | 0 | 0 | | 0 | 0 | | 0 | 1 | 0 | 0 | | 0 | 0 |
| 168 | 2011 SOSORT guidelines: Orthopaedic and rehabilitation treatment of idiopathic scoliosis during growth. Scoliosis. | 1 | 1 | 1 | 0 | 1 | 1 | 1 | 1 | | 1 | 0 | 0 | 0 | 1 | 1 | 1 | | 0 | 1 | 1 | 1 | 1 | | 0 | 0 | 0 | 0 | 0 | 0 | 1 | | 1 | 1 | | 1 | 1 | 0 | 0 | | 1 | 0 |
| 169 | In-hospital rehabilitation after multiple joint procedures of the lower extremities in haemophilia patients: clinical guidelines for physical therapists | 1 | 0 | 1 | 0 | 1 | 1 | 1 | 1 | | 1 | 0 | 0 | 0 | 0 | 1 | 0 | | 0 | 1 | 1 | 0 | 1 | | 0 | 0 | 0 | 0 | 0 | 1 | 0 | | 1 | 1 | | 0 | 1 | 0 | 0 | | 0 | 0 |
| 170 | Physiotherapy in rheumatoid arthritis: development of a practice guideline | 1 | 0 | 1 | 0 | 1 | 1 | 0 | 1 | | 1 | 0 | 1 | 0 | 0 | 1 | 1 | | 0 | 1 | 1 | 1 | 1 | | 0 | 0 | 0 | 0 | 0 | 1 | 1 | | 0 | 0 | | 0 | 0 | 0 | 0 | | 1 | 1 |
| 171 | KNGF Guideline for Physical Therapy in patients with Osteoporosis | 1 | 0 | 0 | 0 | 1 | 0 | 1 | 1 | | 1 | 0 | 1 | 0 | 0 | 0 | 0 | | 0 | 0 | 0 | 0 | 1 | | 0 | 0 | 0 | 0 | 0 | 0 | 0 | | 0 | 0 | | 0 | 0 | 0 | 0 | | 0 | 0 |
| 172 | KNGF Guideline for Physical Therapy in patients with Stress urinary incontinence | 1 | 0 | 1 | 0 | 1 | 0 | 1 | 1 | | 1 | 0 | 1 | 0 | 0 | 0 | 0 | | 0 | 0 | 0 | 0 | 1 | | 0 | 0 | 0 | 0 | 0 | 0 | 0 | | 0 | 0 | | 0 | 0 | 0 | 0 | | 0 | 1 |
| 173 | KNGF Clinical Practice Guideline for physical therapy in patients undergoing cardiac rehabilitation | 1 | 0 | 1 | 0 | 0 | 0 | 1 | 1 | | 1 | 0 | 1 | 0 | 0 | 0 | 0 | | 0 | 1 | 1 | 0 | 1 | | 0 | 0 | 0 | 0 | 0 | 0 | 1 | | 0 | 0 | | 0 | 0 | 0 | 1 | | 0 | 1 |
| 174 | S1 guideline: dermatological inpatient rehabilitation in adult atopic dermatitis | 1 | 0 | 1 | 0 | 0 | 1 | 0 | 1 | | 1 | 0 | 1 | 1 | 0 | 1 | 0 | | 0 | 0 | 0 | 0 | 1 | | 0 | 0 | 0 | 0 | 0 | 1 | 0 | | 0 | 1 | | 1 | 0 | 0 | 1 | | 0 | 0 |
| 175 | Optimizing pulmonary rehabilitation in chronic obstructive pulmonary disease--practical issues: a Canadian Thoracic Society Clinical Practice Guideline | 1 | 0 | 1 | 0 | 1 | 1 | 1 | 1 | | 1 | 0 | 1 | 1 | 0 | 1 | 1 | | 0 | 0 | 0 | 1 | 1 | | 0 | 0 | 0 | 1 | 0 | 1 | 1 | | 1 | 1 | | 1 | 1 | 1 | 1 | | 1 | 1 |
| 176 | The American Society of Shoulder and Elbow Therapists' consensus rehabilitation guideline for arthroscopic anterior capsulolabral repair of the shoulder | 1 | 0 | 1 | 0 | 1 | 1 | 1 | 1 | | 0 | 0 | 1 | 0 | 0 | 1 | 0 | | 0 | 0 | 0 | 0 | 1 | | 0 | 0 | 0 | 0 | 0 | 1 | 0 | | 1 | 0 | | 0 | 0 | 0 | 0 | | 1 | 1 |
| 177 | KNGF Guideline for Physical Therapy in patients with Osteoarthritis of the hip and knee | 1 | 0 | 1 | 1 | 0 | 0 | 1 | 1 | | 1 | 0 | 1 | 0 | 0 | 0 | 0 | | 1 | 1 | 1 | 0 | 1 | | 0 | 0 | 0 | 0 | 0 | 0 | 0 | | 0 | 0 | | 0 | 0 | 0 | 0 | | 0 | 0 |
| 178 | Management of patients with stroke: rehabilitation, prevention and management of complications, and discharge planning : a national clinical guideline | 1 | 0 | 1 | 1 | 1 | 0 | 1 | 1 | | 1 | 0 | 1 | 0 | 1 | 1 | 1 | | 0 | 0 | 0 | 0 | 1 | | 1 | 0 | 1 | 1 | 0 | 1 | 1 | | 1 | 0 | | 0 | 0 | 0 | 1 | | 0 | 0 |
| 179 | Rehabilitation after critical illness in adults | 1 | 0 | 1 | 1 | 1 | 0 | 1 | 1 | | 1 | 1 | 1 | 1 | 1 | 1 | 1 | | 0 | 1 | 1 | 1 | 1 | | 1 | 0 | 0 | 0 | 0 | 1 | 1 | | 1 | 0 | | 0 | 0 | 0 | 1 | | 1 | 1 |
| 180 | Hip pain and mobility deficits--hip osteoarthritis: clinical practice guidelines linked to the international classification of functioning, disability, and health from the orthopaedic section of the American Physical Therapy Association | 1 | 0 | 1 | 1 | 1 | 1 | 1 | 1 | | 1 | 0 | 1 | 0 | 0 | 1 | 0 | | 0 | 0 | 0 | 1 | 1 | | 0 | 1 | 0 | 0 | 0 | 1 | 1 | | 1 | 0 | | 0 | 0 | 0 | 1 | | 0 | 0 |
| 181 | Ottawa Panel evidence-based clinical practice guidelines for strengthening exercises in the management of fibromyalgia | 1 | 0 | 1 | 0 | 1 | 0 | 1 | 1 | | 1 | 0 | 1 | 0 | 1 | 1 | 1 | | 0 | 1 | 1 | 1 | 1 | | 0 | 1 | 0 | 0 | 0 | 1 | 0 | | 1 | 1 | | 0 | 0 | 0 | 0 | | 0 | 1 |
| 182 | Mental wellbeing in over 65s occupational therapy and physical activity interventions | 1 | 0 | 1 | 0 | 1 | 0 | 1 | 1 | | 1 | 1 | 1 | 1 | 1 | 1 | 1 | | 0 | 1 | 1 | 1 | 1 | | 1 | 0 | 0 | 0 | 0 | 1 | 0 | | 1 | 0 | | 0 | 0 | 0 | 1 | | 1 | 1 |
| 183 | Neck pain: Clinical practice guidelines linked to the International Classification of Functioning, Disability, and Health from the Orthopedic Section of the American Physical Therapy Association | 1 | 0 | 1 | 0 | 1 | 0 | 1 | 1 | | 1 | 0 | 1 | 0 | 0 | 1 | 0 | | 0 | 1 | 1 | 1 | 1 | | 0 | 1 | 0 | 0 | 0 | 1 | 1 | | 1 | 0 | | 0 | 0 | 0 | 1 | | 0 | 0 |
| 184 | KNGF Clinical Practice Guideline for physical therapy in patients with COPD | 1 | 0 | 1 | 1 | 1 | 0 | 1 | 1 | | 1 | 0 | 1 | 0 | 0 | 0 | 0 | | 0 | 0 | 0 | 0 | 1 | | 0 | 0 | 0 | 0 | 0 | 0 | 0 | | 0 | 0 | | 0 | 0 | 0 | 0 | | 1 | 1 |
| 185 | Internal impingement in the tennis player: rehabilitation guidelines | 1 | 0 | 1 | 1 | 1 | 0 | 1 | 1 | | 1 | 0 | 0 | 0 | 0 | 1 | 0 | | 0 | 0 | 0 | 0 | 1 | | 1 | 0 | 0 | 0 | 0 | 0 | 0 | | 0 | 0 | | 0 | 1 | 0 | 0 | | 0 | 0 |
| 186 | Clinical Competency Guidelines for Pulmonary Rehabilitation Professionals: American Association of Cardiovascular and Pulmonary Rehabilitation Position Statement | 1 | 0 | 1 | 0 | 1 | 0 | 0 | 1 | | 1 | 0 | 1 | 0 | 0 | 1 | 1 | | 0 | 1 | 0 | 0 | 1 | | 0 | 0 | 0 | 0 | 0 | 0 | 0 | | 0 | 0 | | 0 | 1 | 0 | 0 | | 0 | 1 |
| 187 | Pulmonary rehabilitation: Joint ACCP/AACVPR evidence-based clinical practice guidelines | 1 | 0 | 1 | 1 | 1 | 0 | 1 | 1 | | 1 | 0 | 1 | 1 | 0 | 1 | 0 | | 0 | 1 | 1 | 1 | 1 | | 0 | 1 | 0 | 0 | 0 | 1 | 0 | | 1 | 0 | | 0 | 1 | 1 | 1 | | 1 | 0 |
| 188 | Ottawa panel evidence-based clinical practice guidelines for post-stroke rehabilitation | 1 | 0 | 1 | 1 | 1 | 0 | 1 | 1 | | 1 | 0 | 1 | 0 | 1 | 1 | 1 | | 1 | 1 | 1 | 1 | 1 | | 0 | 1 | 0 | 0 | 0 | 1 | 1 | | 1 | 1 | | 0 | 0 | 0 | 0 | | 0 | 1 |
| 189 | KNGF Guideline for Physical Therapy in patients with acute ankle sprain | 1 | 0 | 1 | 1 | 1 | 0 | 1 | 1 | | 1 | 0 | 1 | 0 | 0 | 0 | 0 | | 0 | 0 | 0 | 0 | 1 | | 0 | 0 | 0 | 0 | 0 | 0 | 0 | | 0 | 0 | | 0 | 0 | 0 | 1 | | 0 | 0 |
| 190 | Ottawa Panel evidence-based clinical practice guidelines for therapeutic exercises and manual therapy in the treatment of osteoarthritis | 1 | 0 | 1 | 1 | 1 | 0 | 1 | 1 | | 1 | 0 | 1 | 0 | 1 | 1 | 1 | | 1 | 1 | 1 | 1 | 1 | | 0 | 1 | 0 | 0 | 0 | 1 | 1 | | 1 | 1 | | 0 | 0 | 0 | 0 | | 1 | 1 |
| 191 | Management of Adult Stroke Rehabilitation Care: a clinical practice guideline | 1 | 0 | 1 | 1 | 1 | 0 | 1 | 1 | | 1 | 0 | 1 | 1 | 0 | 1 | 1 | | 0 | 1 | 1 | 1 | 1 | | 1 | 0 | 0 | 0 | 0 | 0 | 0 | | 1 | 0 | | 0 | 0 | 0 | 0 | | 0 | 0 |
| 192 | Evidence-Based Clinical Practice Guidelines for Interdisciplinary Rehabilitation of Chronic Nonmalignant Pain Syndrome Patients | 1 | 1 | 1 | 1 | 1 | 1 | 1 | 1 | | 1 | 0 | 1 | 0 | 0 | 1 | 0 | | 0 | 1 | 0 | 0 | 1 | | 1 | 0 | 0 | 0 | 0 | 0 | 0 | | 0 | 0 | | 0 | 0 | 0 | 0 | | 1 | 1 |
| 193 | Italian guidelines on rehabilitation treatment of adolescents with scoliosis or other spinal deformities | 1 | 0 | 1 | 1 | 1 | 0 | 1 | 1 | | 1 | 0 | 1 | 0 | 1 | 1 | 0 | | 0 | 1 | 1 | 1 | 1 | | 1 | 1 | 0 | 0 | 0 | 1 | 1 | | 0 | 0 | | 0 | 0 | 0 | 1 | | 0 | 1 |
| 194 | EFNS guidelines on cognitive rehabilitation: report of an EFNS task force | 1 | 0 | 1 | 1 | 1 | 0 | 1 | 1 | | 1 | 1 | 0 | 0 | 0 | 1 | 0 | | 0 | 1 | 1 | 1 | 1 | | 1 | 0 | 0 | 0 | 0 | 0 | 0 | | 1 | 0 | | 0 | 0 | 0 | 0 | | 1 | 0 |
| 195 | Guidelines for rehabilitation of children with cerebral palsy | 1 | 0 | 1 | 1 | 1 | 1 | 1 | 1 | | 1 | 0 | 1 | 1 | 0 | 1 | 0 | | 0 | 1 | 1 | 1 | 1 | | 1 | 0 | 0 | 0 | 0 | 1 | 1 | | 1 | 0 | | 0 | 0 | 0 | 1 | | 0 | 0 |
| 196 | Ottawa Panel Evidence-Based Clinical Practice Guidelines for Electrotherapy and Thermotherapy Interventions in the Management of Rheumatoid Arthritis in Adults | 1 | 0 | 1 | 0 | 1 | 1 | 1 | 1 | | 1 | 0 | 1 | 0 | 0 | 1 | 1 | | 1 | 1 | 1 | 1 | 1 | | 0 | 1 | 0 | 0 | 0 | 1 | 1 | | 1 | 0 | | 0 | 0 | 0 | 0 | | 0 | 1 |
| 197 | EFNS guidelines on cognitive rehabilitation report of an EFNS task force | 1 | 0 | 1 | 0 | 1 | 1 | 1 | 1 | | 1 | 1 | 0 | 0 | 0 | 1 | 0 | | 0 | 1 | 1 | 1 | 1 | | 1 | 0 | 0 | 0 | 0 | 0 | 1 | | 1 | 0 | | 0 | 0 | 0 | 1 | | 1 | 0 |
| 198 | Health professional's guide to rehabilitation of the patient with osteoporosis | 1 | 0 | 1 | 0 | 1 | 1 | 1 | 1 | | 1 | 0 | 1 | 0 | 0 | 1 | 1 | | 0 | 0 | 0 | 0 | 1 | | 0 | 0 | 0 | 0 | 0 | 0 | 0 | | 0 | 0 | | 0 | 0 | 0 | 0 | | 1 | 0 |
| 199 | Occupational therapy and physiotherapy for the patient with burns: principles and management guidelines | 1 | 0 | 1 | 0 | 1 | 1 | 1 | 1 | | 1 | 0 | 0 | 0 | 0 | 1 | 0 | | 0 | 0 | 0 | 0 | 1 | | 0 | 0 | 0 | 0 | 0 | 1 | 0 | | 1 | 0 | | 0 | 0 | 0 | 0 | | 0 | 1 |
| 200 | Clinical practice guideline for the physiotherapy of patients with whiplash-associated disorders | 1 | 0 | 1 | 0 | 1 | 1 | 1 | 1 | | 1 | 1 | 0 | 0 | 0 | 1 | 0 | | 0 | 1 | 1 | 1 | 1 | | 1 | 1 | 0 | 0 | 0 | 1 | 1 | | 1 | 0 | | 0 | 0 | 0 | 0 | | 1 | 0 |
| 201 | Philadelphia Panel Evidence-Based Clinical Practice Guidelines on Selected Rehabilitation Interventions: Overview and Methodology | 1 | 0 | 1 | 0 | 1 | 1 | 1 | 1 | | 1 | 0 | 1 | 0 | 1 | 1 | 1 | | 1 | 1 | 1 | 1 | 1 | | 0 | 1 | 0 | 0 | 0 | 1 | 1 | | 0 | 1 | | 0 | 0 | 0 | 0 | | 0 | 1 |
| 202 | Philadelphia Panel evidence-based clinical practice guidelines on selected rehabilitation interventions for low back pain | 1 | 0 | 1 | 0 | 1 | 1 | 1 | 1 | | 1 | 0 | 1 | 0 | 1 | 1 | 0 | | 0 | 1 | 1 | 1 | 1 | | 1 | 1 | 0 | 0 | 0 | 1 | 1 | | 1 | 1 | | 0 | 0 | 0 | 0 | | 1 | 1 |
| 203 | Philadelphia Panel evidence-based clinical practice guidelines on selected rehabilitation interventions for knee pain | 1 | 0 | 1 | 0 | 1 | 1 | 1 | 1 | | 1 | 0 | 1 | 0 | 1 | 1 | 0 | | 0 | 1 | 1 | 1 | 1 | | 1 | 1 | 0 | 0 | 0 | 1 | 1 | | 0 | 1 | | 0 | 0 | 0 | 0 | | 1 | 1 |
| 204 | Upper Extremity Rehabilitation in Women with Breast Cancer after Axillary Dissection: Clinical Practice Guidelines | 1 | 0 | 1 | 0 | 1 | 1 | 0 | 1 | | 1 | 1 | 1 | 0 | 0 | 1 | 0 | | 0 | 1 | 0 | 0 | 1 | | 1 | 1 | 0 | 0 | 0 | 1 | 0 | | 1 | 0 | | 0 | 0 | 0 | 0 | | 1 | 1 |
| 205 | Pulmonary rehabilitation British Thoracic Society Standards of Care Subcommittee on Pulmonary Rehabilitation | 0 | 0 | 1 | 1 | 1 | 1 | 1 | 1 | | 1 | 0 | 1 | 0 | 0 | 1 | 0 | | 0 | 1 | 0 | 0 | 1 | | 1 | 0 | 0 | 0 | 0 | 0 | 0 | | 1 | 0 | | 0 | 0 | 0 | 0 | | 1 | 0 |
| 206 | Philadelphia panel evidence-based clinical practice guidelines on selected rehabilitation interventions for shoulder pain | 1 | 0 | 1 | 0 | 1 | 1 | 1 | 1 | | 1 | 0 | 1 | 0 | 1 | 1 | 0 | | 0 | 1 | 1 | 1 | 1 | | 1 | 1 | 0 | 0 | 0 | 1 | 1 | | 1 | 1 | | 0 | 0 | 0 | 0 | | 1 | 1 |
| 207 | Philadelphia Panel evidence-based clinical practice guidelines on selected rehabilitation interventions for neck pain | 1 | 0 | 1 | 0 | 1 | 1 | 1 | 1 | | 1 | 0 | 1 | 0 | 1 | 1 | 0 | | 0 | 1 | 1 | 1 | 1 | | 1 | 1 | 0 | 0 | 0 | 1 | 1 | | 1 | 1 | | 0 | 0 | 0 | 0 | | 1 | 1 |
| 208 | Therapeutic Exercise Guidelines for Rehabilitating Lumbar Spine Injuries in Athletes | 1 | 0 | 1 | 1 | 1 | 0 | 0 | 1 | | 1 | 0 | 1 | 0 | 0 | 1 | 0 | | 0 | 0 | 0 | 0 | 1 | | 0 | 0 | 0 | 0 | 0 | 0 | 0 | | 0 | 0 | | 0 | 0 | 0 | 0 | | 0 | 0 |
| 209 | Cardiac rehabilitation in the United Kingdom: guidelines and audit standards | 1 | 0 | 1 | 0 | 1 | 0 | 0 | 1 | | 1 | 1 | 0 | 0 | 0 | 1 | 0 | | 0 | 0 | 0 | 0 | 1 | | 1 | 0 | 0 | 0 | 0 | 1 | 0 | | 1 | 0 | | 0 | 0 | 0 | 1 | | 0 | 1 |
| 210 | Cardiac Rehabilitation as Secondary Prevention: Quick Reference Guide for Clinicians | 1 | 0 | 1 | 0 | 1 | 1 | 1 | 1 | | 1 | 0 | 1 | 0 | 1 | 1 | 1 | | 1 | 1 | 1 | 0 | 1 | | 1 | 0 | 0 | 0 | 0 | 1 | 0 | | 1 | 0 | | 0 | 0 | 0 | 1 | | 0 | 1 |
| 211 | Guidelines for Cognitive Rehabilitation | 1 | 0 | 1 | 0 | 1 | 0 | 1 | 1 | | 1 | 0 | 0 | 0 | 0 | 1 | 1 | | 0 | 0 | 0 | 0 | 1 | | 0 | 0 | 0 | 0 | 0 | 1 | 0 | | 1 | 0 | | 0 | 0 | 0 | 0 | | 0 | 0 |
| 212 | ASSOBRAFIR clinical practice guidelines in cardiovascular physical therapy: Exercise-based interventions in outpatient rehabilitation programs for heart failure | 1 | 0 | 1 | 1 | 1 | 1 | 1 | 1 | | 1 | 0 | 1 | 1 | 1 | 0 | 1 | | 1 | 1 | 1 | 1 | 1 | | 0 | 1 | 1 | 1 | 1 | 1 | 1 | | 1 | 1 | | 1 | 1 | 0 | 1 | | 1 |  |
| 213 | ASSOBRAFIR clinical practice guidelines in respiratory physical therapy: Exercise-based interventions in people with chronic obstructive pulmonary disease (COPD) | 1 | 0 | 1 | 1 | 1 | 1 | 1 | 1 | | 1 | 1 | 1 | 1 | 0 | 1 | 1 | | 1 | 1 | 1 | 1 | 1 | | 0 | 1 | 1 | 1 | 1 | 1 | 1 | | 1 | 1 | | 1 | 1 | 0 | 1 | | 1 |  |
| 214 | Australian clinical practice guideline for physical rehabilitation and mobilisation in adult intensive care units | 1 | 0 | 1 | 1 | 1 | 1 | 1 | 1 | | 1 | 0 | 1 | 1 | 1 | 1 | 1 | | 1 | 1 | 1 | 1 | 1 | | 1 | 1 | 1 | 1 | 1 | 1 | 1 | | 1 | 1 | | 1 | 1 | 0 | 1 | | 1 |  |
| 215 | Canadian Stroke Best Practice Recommendations Rehabilitation, Recovery and Community Participation Following Stroke. Part One Stroke Rehabilitation Planning for Optimal Care Delivery, 7th Edition Update 2025 | 1 | 1 | 1 | 1 | 1 | 1 | 1 | 1 | | 1 | 1 | 1 | 1 | 1 | 1 | 1 | | 1 | 1 | 1 | 1 | 1 | | 1 | 1 | 1 | 1 | 1 | 1 | 1 | | 1 | 1 | | 0 | 1 | 0 | 1 | | 1 |  |
| 216 | Canadian Stroke Best Practice Recommendations Rehabilitation, Recovery, and Community Participation Following Stroke, Part Three Optimizing Activity and Community Participation Following Stroke , 7th Edition Update | 1 | 1 | 1 | 1 | 1 | 1 | 1 | 1 | | 1 | 1 | 1 | 1 | 1 | 1 | 1 | | 1 | 1 | 1 | 1 | 1 | | 1 | 1 | 1 | 1 | 1 | 1 | 1 | | 1 | 1 | | 0 | 1 | 0 | 1 | | 1 |  |
| 217 | Canadian Stroke Best Practice Recommendations Rehabilitation, Recovery, and Community Participation Following Stroke, Part Two Delivery of Stroke Rehabilitation to Optimize Functional Recovery, 7th Edition Update | 1 | 1 | 1 | 1 | 1 | 1 | 1 | 1 | | 1 | 1 | 1 | 1 | 1 | 1 | 1 | | 1 | 1 | 1 | 1 | 1 | | 1 | 1 | 1 | 1 | 1 | 1 | 1 | | 1 | 1 | | 0 | 1 | 0 | 1 | | 1 |  |
| 218 | Chinese Guideline for the Rehabilitation Treatment of Osteoporosis (2024 Edition) | 1 | 1 | 1 | 1 | 1 | 1 | 1 | 1 | | 1 | 0 | 1 | 1 | 1 | 1 | 1 | | 1 | 1 | 1 | 1 | 1 | | 1 | 1 | 1 | 1 | 1 | 1 | 1 | | 1 | 1 | | 0 | 1 | 1 | 1 | | 1 |  |
| 219 | Clinical guideline on the open arthrolysis for post-traumatic elbow stiffness in adult patients | 1 | 0 | 1 | 1 | 1 | 1 | 1 | 1 | | 1 | 0 | 1 | 1 | 1 | 1 | 1 | | 1 | 1 | 1 | 1 | 1 | | 1 | 1 | 1 | 0 | 1 | 1 | 1 | | 1 | 1 | | 1 | 1 | 1 | 1 | | 1 |  |
| 220 | Clinical practice guidelines for rehabilitation following surgical management of chronic lateral ankle instability: enhancing recovery based on available evidence | 1 | 0 | 1 | 1 | 1 | 1 | 1 | 1 | | 1 | 0 | 1 | 0 | 1 | 0 | 1 | | 1 | 1 | 1 | 1 | 1 | | 1 | 1 | 0 | 0 | 0 | 1 | 1 | | 0 | 1 | | 0 | 1 | 0 | 0 | | 0 |  |
| 221 | Development of clinical practice guidelines for rehabilitation after diagnosis for primary bone and soft tissue tumours | 1 | 0 | 1 | 1 | 0 | 1 | 1 | 1 | | 1 | 0 | 1 | 0 | 1 | 0 | 1 | | 1 | 1 | 1 | 0 | 1 | | 0 | 0 | 0 | 0 | 0 | 0 | 1 | | 1 | 1 | | 0 | 1 | 0 | 0 | | 0 |  |
| 222 | European Stroke Organisation (ESO) guideline on aphasia rehabilitation | 1 | 0 | 1 | 1 | 1 | 1 | 1 | 1 | | 1 | 1 | 1 | 1 | 1 | 0 | 1 | | 1 | 1 | 1 | 1 | 1 | | 1 | 1 | 1 | 1 | 1 | 1 | 1 | | 1 | 1 | | 1 | 1 | 0 | 1 | | 0 |  |
| 223 | European Stroke Organisation (ESO) guideline on motor rehabilitation | 1 | 0 | 1 | 1 | 1 | 1 | 1 | 1 | | 1 | 0 | 1 | 1 | 1 | 0 | 1 | | 1 | 1 | 1 | 1 | 1 | | 1 | 1 | 1 | 0 | 0 | 1 | 1 | | 1 | 1 | | 0 | 1 | 0 | 0 | | 1 |  |
| 224 | Guidelines for Rehabilitation Interventions in Older Adults With Sarcopenia (2025 Edition) | 1 | 1 | 1 | 1 | 1 | 1 | 1 | 1 | | 1 | 1 | 1 | 1 | 1 | 0 | 1 | | 1 | 1 | 1 | 1 | 1 | | 1 | 1 | 1 | 1 | 1 | 1 | 1 | | 1 | 1 | | 0 | 1 | 1 | 1 | | 0 |  |
| 225 | National Institute for Health and Care Excellence Clinical GuidelinesIn Rehabilitation for chronic neurological disorders including acquired brain injury | 1 | 0 | 1 | 1 | 1 | 1 | 1 | 1 | | 1 | 1 | 1 | 1 | 1 | 0 | 0 | | 0 | 0 | 0 | 0 | 0 | | 0 | 0 | 1 | 1 | 1 | 1 | 1 | | 1 | 0 | | 0 | 0 | 0 | 1 | | 0 | 0 |
| 226 | Rehabilitation of the lower extremities, standing and walking function in people with spinal cord injury or disease Guideline of the German-Speaking Medical Society for Spinal Cord Injury | 1 | 0 | 1 | 1 | 1 | 1 | 1 | 1 | | 1 | 0 | 1 | 0 | 1 | 0 | 1 | | 1 | 1 | 1 | 1 | 1 | | 0 | 1 | 1 | 0 | 1 | 1 | 1 | | 1 | 1 | | 0 | 1 | 0 | 1 | | 1 | 0 |
| 227 | Rotator Cuff Tendinopathy Diagnosis, Nonsurgical Medical Care, and Rehabilitation: A Clinical Practice Guideline | 1 | 0 | 1 | 1 | 1 | 1 | 1 | 1 | | 1 | 1 | 1 | 1 | 1 | 0 | 1 | | 1 | 1 | 1 | 1 | 1 | | 1 | 1 | 1 | 1 | 1 | 1 | 0 | | 1 | 1 | | 0 | 1 | 0 | 1 | | 0 | 0 |

**Table S9. RIGHT reporting rates of is included Clinical Practice Guidelines.**

| RIGHT checklist | | **Reported** | | **Not reported** | |
| --- | --- | --- | --- | --- | --- |
|  |  | **N** | **(%)** | **N** | **(%)** |
| **Basic information** | | | | | |
| 1a | Identify the report as a guideline, that is, with “guideline(s)” or “recommendation(s)” in the title. | 217 | 95.6 | 10 | 4.4 |
| 1b | Describe the year of publication of the guideline. | 47 | 20.7 | 180 | 79.3 |
| 1c | Describe the focus of the guideline, such as screening, diagnosis, treatment, management, prevention, or others. | 220 | 96.9 | 7 | 3.1 |
| 2a | Provide a summary of the recommendations contained in the guideline. | 107 | 47.1 | 120 | 52.9 |
| 3a | Define new or key terms, and provide a list of abbreviations and acronyms if applicable. | 193 | 85.0 | 34 | 15.0 |
| 4a | Identify at least 1 corresponding developer or author who can be contacted about the guideline. | 176 | 77.5 | 51 | 22.5 |
| **Background** | | | | | |
| 5a | Describe the basic epidemiology of the problem, such as the prevalence/incidence, morbidity, mortality, and burden (including financial) resulting from the problem. | 200 | 88.1 | 27 | 11.9 |
| 6a | Describe the aim(s) of the guideline and specific objectives, such as improvements in health indicators (e.g., mortality and disease prevalence), quality of life, or cost savings. | 214 | 94.3 | 13 | 5.7 |
| 7a | Describe the primary population(s) that is affected by the recommendation(s) in the guideline. | 205 | 90.3 | 22 | 9.7 |
| 7b | Describe any subgroups that are given special consideration in the guideline. | 41 | 18.1 | 186 | 81.9 |
| 8a | Describe the intended primary users of the guideline (such as primary care providers, clinical specialists, public health practitioners, program managers, and policymakers) and other potential users of the guideline. | 164 | 72.2 | 63 | 27.8 |
| 8b | Describe the setting(s) for which the guideline is intended, such as primary care, low- and middle-income countries, or inpatient facilities. | 64 | 28.2 | 163 | 71.8 |
| 9a | Describe how all contributors to the guideline development were selected and their roles and responsibilities (e.g., steering group, guideline panel, external reviewers, systematic review team, and methodologists) | 131 | 57.7 | 96 | 42.3 |
| 9b | List all individuals involved in developing the guideline, including their title, role(s), and institutional affiliation(s). | 177 | 78.0 | 50 | 22.0 |
| **Evidence** | | | | | |
| 10a | State the key questions that were the basis for the recommendations in PICO (population, intervention, comparator, and outcome) or other format as appropriate. | 124 | 54.6 | 103 | 45.4 |
| 10b | Indicate how the outcomes were selected and sorted. | 86 | 37.9 | 141 | 62.1 |
| 11a | Indicate whether the guideline is based on new systematic reviews done specifically for this guideline or whether existing systematic reviews were used. | 159 | 70.0 | 68 | 30.0 |
| 11b | If the guideline developers used existing systematic reviews, reference these and describe how those reviews were identified and assessed (provide the search strategies and the selection criteria, and describe how the risk of bias was evaluated) and whether they were updated. | 147 | 64.8 | 80 | 35.2 |
| 12a | Describe the approach used to assess the certainty of the body of evidence. | 155 | 68.3 | 72 | 31.7 |
| **Recommendations** | | | | | |
| 13a | Provide clear, precise, and actionable recommendations. | 212 | 93.4 | 15 | 6.6 |
| 13b | Present separate recommendations for important subgroups if the evidence suggests that there are important differences in factors influencing recommendations, particularly the balance of benefits and harms across subgroups. | 152 | 67.0 | 75 | 33.0 |
| 13c | Indicate the strength of recommendations and the certainty of the supporting evidence. | 148 | 65.2 | 79 | 34.8 |
| 14a | Describe whether values and preferences of the target population(s) were considered in the formulation of each recommendation. If yes, describe the approaches and methods used to elicit or identify these values and preferences. If values and preferences were not considered, provide an explanation. | 86 | 37.9 | 141 | 62.1 |
| 14b | Describe whether cost and resource implications were considered in the formulation of recommendations. If yes, describe the specific approaches and methods used (such as cost-effectiveness analysis) and summarize the results. If resource issues were not considered, provide an explanation. | 80 | 35.2 | 147 | 64.8 |
| 14c | Describe other factors taken into consideration when formulating the recommendations, such as equity, feasibility, and acceptability. | 67 | 29.5 | 160 | 70.5 |
| 15a | Describe the processes and approaches used by the guideline development group to make decisions, particularly the formulation of recommendations (such as how consensus was defined and achieved and whether voting was used). | 140 | 61.7 | 87 | 38.3 |
| **Review and quality assurance** | | | | | |
| 16a | Indicate whether the draft guideline underwent independent review and, if so, how this was executed and the comments considered and addressed. | 126 | 55.5 | 101 | 44.5 |
| 17a | Indicate whether the guideline was subjected to a quality assurance process. If yes, describe the process. | 152 | 67.0 | 75 | 33.0 |
| **Funding and declaration and management of interests** | | | | | |
| 18a | Describe the specific sources of funding for all stages of guideline development. | 153 | 67.4 | 74 | 32.6 |
| 18b | Describe the role of funder(s) in the different stages of guideline development and in the dissemination and implementation of the recommendations. | 61 | 26.9 | 166 | 73.1 |
| 19a | Describe what types of conflicts (financial and nonfinancial) were relevant to guideline development. | 137 | 60.4 | 90 | 39.6 |
| 19b | Describe how conflicts of interest were evaluated and managed and how users of the guideline can access the declarations. | 49 | 21.6 | 178 | 78.4 |
| **Other information** | | | | | |
| 20a | Describe where the guideline, its appendices, and other related documents can be accessed. | 119 | 52.4 | 108 | 47.6 |
| 21a | Describe the gaps in the evidence and/or provide suggestions for future research. | 134 | 59.0 | 93 | 41.0 |
| 22a | Describe any limitations in the guideline development process (such as the development groups were not multidisciplinary or patients' values and preferences were not sought), and indicate how these limitations might have affected the validity of the recommendations. | 134 | 59.0 | 93 | 41.0 |

**Factors Associated with Guideline Quality**

**Table S10 The characteristics of CPGs by quality status.**

| **Categories** | | **High-quality CPGs No.%** | **Low-quality CPGs No.%** |
| --- | --- | --- | --- |
| Overall | | 32 (14.1) | 195 (85.9) |
| Year of publication | 1992-2010 | 0 | 39 (100) |
|  | 2011-2016 | 5 (10.9) | 41 (89.1) |
|  | 2017-2020 | 7 (12.1) | 51 (87.9) |
|  | 2021-2026 | 20 (23.8) | 64 (76.2) |
| Region | America | 23 (26.7) | 63 (73.3) |
|  | Europe | 2 (4.1) | 47 (95.9) |
|  | Oceania | 1 (20.0) | 4 (80.0) |
|  | Asia | 6 (8.1) | 68 (91.9) |
|  | Transcontinental | 0 | 3 (100) |
|  | Combination | 0 | 10(100) |
| Type of institution | Discipline-or disorder-focused professional organization | 18 (14.5) | 106 (85.5) |
|  | University or hospital | 4 (12.5) | 28 (87.5) |
|  | CPG creating specialist entity | 0 | 24 (100) |
|  | Provincial, state, or national health department | 2 (22.2) | 7 (77.8) |
|  | Not stated, combination, other, unclear | 8 (21.1) | 30 (78.9) |
| Funding | Government | 9 (13.4) | 58 (86.6) |
|  | Industry | 0 | 2 (100) |
|  | Guideline society | 18 (30.0) | 42 (70.0) |
|  | Combination | 1 (11.1) | 8 (88.9) |
|  | Not reported | 4 (4.5) | 85 (95.5) |
| Type of guideline | Standard | 32 (14.4) | 190 (85.6) |
|  | Consolidated | 0 | 1 (100) |
|  | Guidelines produced in response to an emergency or urgent need | 0 | 4 (100) |
|  | Adapted | 0 | 0 |
| Publication platform | Journal only | 0 | 0 |
|  | online only | 2 (10.5) | 17 (89.5) |
|  | Journal and online | 30 (14.4) | 178 (85.6) |
| No. of authors | >20 | 11 (27.5) | 29 (72.5) |
|  | 11-20 | 4 (10.8) | 33 (89.2) |
|  | 6-10 | 6 (11.8) | 45 (88.2) |
|  | ≤5 | 11 (11.1) | 88 (88.9) |
| No. of panel members | >60 | 0 | 14 (100) |
|  | 31-60 | 11 (23.4) | 36 (76.6) |
|  | 11-30 | 7 (21.9) | 25 (78.1) |
|  | ≤10 | 7 (21.2) | 26 (78.8) |
|  | Not listed | 7 (6.9) | 94 (93.1) |
| Disease topic | Cardiac disorders | 3 (15.8) | 16 (84.2) |
|  | Pulmonary disorders | 1 (5.3) | 18 (94.7) |
|  | Spinal cord injury | 2 (16.7) | 10 (83.3) |
|  | Stroke | 7 (21.9) | 25 (78.1) |
|  | Arthritis | 5 (20.8) | 19 (79.2) |
|  | Others | 14 (11.6) | 107 (88.4) |
| Evidence grading system | GRADE system | 21 (22.6) | 72 (77.4) |
|  | Oxford system | 2 (20.0) | 8 (80.0) |
|  | Other system | 9 (11.7) | 68 (88.3) |
|  | None | 0 | 47 (100) |
| Method for guideline development | Systematic review | 32 (18.1) | 145 (81.9) |
|  | Consensus or narrative review | 0 | 11 (100) |
|  | Not mentioned | 0 | 38 (100) |
|  | Adapted or adopted | 0 | 1 (100) |
| Recommendation methods | Informal consensus | 7 (11.7) | 53 (88.3) |
|  | Formal consensus | 18 (23.1) | 60 (76.9) |
|  | Not mentioned | 7 (7.9) | 82 (92.1) |
| No. of recommendations | 1-10 | 17 (19.3) | 71 (80.7) |
|  | 11-20 | 7 (9.5) | 67 (90.5) |
|  | 21-30 | 4 (13.8) | 25 (86.2) |
|  | 31-40 | 2 (16.7) | 10 (83.3) |
|  | ≥41 | 2 (8.3) | 22 (91.7) |
| Quality tool referral | AGREEII | 11 (27.5) | 29 (72.5) |
|  | RIGHT | 0 | 5 (100) |
|  | Combination | 4 (40.0) | 6 (60.0) |
|  | Not reported | 17 (9.9) | 155 (90.1) |
| External review | Yes | 30 (28.3) | 76 (71.7) |
|  | No | 2 (1.7) | 119 (98.3) |
| Defined time to update | Yes | 22 (28.2) | 56 (71.8) |
|  | No | 10 (6.7) | 139 (93.3) |
| Language | English | 28 (17.2) | 135 (82.8) |
|  | Chinese | 4 (6.2) | 60 (93.8) |
| Version | Old | 5 (22.7) | 17 (77.3) |
|  | New | 27 (13.2) | 178 (86.8) |
| Publication of AGREE | After | 32 (15.2) | 179 (84.8) |
|  | Before | 0 | 16 (100) |
| Publication of AGREE Ⅱ | After | 32 (17.0) | 156 (83.0) |
|  | Before | 0 | 39 (100) |

**Table S11. Variables related to quality of Clinical Practice Guidelines.**

|  | **Domain 1**  **Scope and Purpose** | **Domain 2**  **Stakeholder Involvement** | **Domain 3**  **Rigor of Development** | **Domain 4**  **Clarity of Presentation** | **Domain 5**  **Applicability** | **Domain 6**  **Editorial Independence** |
| --- | --- | --- | --- | --- | --- | --- |
| **Language** | | | | | | |
| Chinese | 68.88±13.904 | 49.96±16.026 | 43.98±20.385 | 72.87±12.684 | 27.54±16.730 | 43.49±26.539 |
| English | 74.64±15.439 | 52.80±17.661 | 51.37±20.978 | 72.60±16.625 | 39.14±18.615 | 48.47±28.324 |
| *P*-value | 0.004 | 0.242 | 0.062 | 0.952 | 0.000 | 0.290 |
| **Year of Publication** | | | | | | |
| 1992-2010 | 66.38±10.495 | 39.79±11.043 | 40.56±12.977 | 58.62±11.889 | 33.97±11.389 | 41.87±19.476 |
| 2011-2016 | 71.37±18.818 | 45.43±20.209 | 40.35±20.481 | 71.33±15.699 | 34.41±19.07 | 32.65±30.113 |
| 2017-2020 | 72±13.376 | 54.33±15.579 | 49.16±20.731 | 79.26±12.852 | 32.38±17.896 | 49.93±30.876 |
| 2021-2026 | 77.64±14.756 | 59.70±14.385 | 58.42±20.900 | 75.32±14.650 | 40.15±21.382 | 55.50±24.313 |
| *P*-value | 0.002 | 0.000 | 0.000 | 0.000 | 0.201 | 0.000 |
| **Region** | | | | | | |
| North America | 75.63±14.546 | 57.11±15.219 | 56.33±17.923 | 73.43±16.800 | 40.82±17.846 | 58.01±26.953 |
| Europe | 71.57±16.069 | 45.33±20.344 | 43.04±21.218 | 67.27±16.734 | 39.29±18.246 | 31.27±25.555 |
| Oceania | 71.80±15.255 | 53.80±22.906 | 48.00±25.475 | 82.20±13.217 | 28.40±32.230 | 59.00±23.822 |
| Asia | 69.91±14.631 | 50.97±15.838 | 45.32±21.464 | 73.69±13.247 | 28.38±16.458 | 45.05±26.559 |
| Transcontinental | 62.00±12.530 | 42.67±13.868 | 43.67±5.508 | 87.00±8.185 | 20.33±15.373 | 52.67±45.611 |
| South America | 82.67±16.166 | 61.67±12.702 | 61.33±31.501 | 66.00±1.732 | 62.67±11.150 | 54.33±21.939 |
| Combination | 82.00±15.312 | 49.30±15.195 | 50.30±25.202 | 77.70±12.193 | 35.50±19.208 | 39.60±14.462 |
| *P*-value | 0.057 | 0.001 | 0.001 | 0.159 | 0.000 | 0.000 |
| **Type of institution** | | | | | | |
| Discipline-or disorder-focused professional organization | 71.66±14.706 | 50.77±14.869 | 48.70±20.131 | 73.16±15.851 | 33.31±17.708 | 51.30±26.629 |
| University or hospital | 72.75±18.660 | 47.78±17.372 | 47.50±23.367 | 69.75±17.129 | 35.09±21.754 | 39.50±27.144 |
| CPG creating specialist entity | 76.92±13.410 | 53.04±21.908 | 42.96±19.387 | 67.58±14.337 | 48.46±13.654 | 20.33±20.025 |
| Provincial, state, or national health department | 77.56±13.258 | 56.11±16.097 | 60.22±14.67 | 73.44±11.348 | 47.78±7.513 | 46.44±25.353 |
| Not stated, combination, other, unclear | 74.00±15.010 | 58.05±20.218 | 54.32±22.892 | 76.42±14.107 | 34.53±20.813 | 56.89±26.702 |
| *P*-value | 0.425 | 0.599 | 0.418 | 0.818 | 0.001 | 0.000 |
| **Foundation** | | | | | | |
| Government | 74.70±14.904 | 54.46±15.948 | 52.46±20.864 | 75.54±13.950 | 33.22±19.285 | 52.03±24.461 |
| Industry | 51.50±17.678 | 41.50±12.021 | 36.00±9.899 | 60.00±9.899 | 39.50±9.192 | 46.00±5.657 |
| Guideline society | 78.48±14.725 | 59.27±16.024 | 57.40±20.429 | 76.85±14.998 | 39.22±20.349 | 57.22±30.449 |
| Combination | 72.89±11.570 | 53.44±13.087 | 54.11±14.769 | 74.89±23.105 | 40.89±14.903 | 64.00±16.140 |
| Not reported | 68.98±14.696 | 45.38±17.172 | 41.33±19.555 | 67.70±15.051 | 35.20±17.746 | 34.90±25.178 |
| *P*-value | 0.008 | 0.000 | 0.001 | 0.004 | 0.267 | 0.000 |
| **Type of guideline** | | | | | | |
| Standard | 73.10±15.297 | 52.08±17.351 | 49.69±20.843 | 72.64±15.709 | 36.23±18.922 | 47.36±27.849 |
| Consolidated | 78.00 | 58.00 | 59.00 | 72.00 | 23.00 | 46.00 |
| Guidelines produced in response to an emergency or urgent need | 66.00±7.071 | 47.25±12.285 | 26.25±21.654 | 73.50±3.873 | 23.50±4.123 | 33.25±34.131 |
| *P*-value | 0.562 | 0.764 | 0.108 | 0.892 | 0.202 | 0.765 |
| **Disease topic** | | | | | | |
| Cardiac disorders | 65.37±17.683 | 48.26±20.121 | 44.47±19.262 | 74.74±12.101 | 37.89±17.515 | 40.58±22.851 |
| Pulmonary disorders | 73.95±13.677 | 55.32±15.213 | 51.63±15.809 | 71.00±13.728 | 38.05±20.980 | 56.37±20.734 |
| Spinal cord injury | 78.08±12.916 | 56.58±15.126 | 52.17±21.775 | 81.33±15.096 | 34.83±15.349 | 56.08±23.558 |
| Stroke | 75.56±12.941 | 54.59±18.635 | 49.69±24.639 | 74.94±12.376 | 38.59±21.531 | 52.12±29.369 |
| Arthritis | 73.46±11.444 | 54.00±15.134 | 50.67±18.091 | 71.13±12.996 | 37.38±18.415 | 46.29±30.840 |
| Others | 72.77±16.176 | 50.56±17.277 | 49.07±21.648 | 71.41±17.314 | 34.43±18.496 | 44.62±28.701 |
| *P*-value | 0.289 | 0.473 | 0.344 | 0.240 | 0.831 | 0.517 |
| **No. of panel members** | | | | | | |
| >60 | 73.14±15.689 | 51.07±15.204 | 47.71±21.058 | 73.93±15.385 | 32.07±22.054 | 47.57±22.034 |
| 31-60 | 72.68±18.110 | 51.70±19.035 | 55.11±19.416 | 74.21±16.935 | 39.89±21.001 | 47.79±25.739 |
| 11-30 | 81.12±11.670 | 61.44±15.756 | 61.25±19.724 | 82.84±14.258 | 39.41±17.413 | 60.03±21.830 |
| ≤10 | 72.15±12.988 | 51.39±17.764 | 47.79±23.262 | 71.76±13.870 | 37.00±18.400 | 49.48±29.587 |
| Not listed | 70.82±14.684 | 49.51±16.169 | 44.41±19.426 | 68.80±14.448 | 33.20±17.610 | 41.85±29.625 |
| *P*-value | 0.036 | 0.029 | 0.001 | 0.001 | 0.267 | 0.069 |
| **Publication platform** | | | | | | |
| Journal only | - | - | - | - | - | - |
| online only | 81.47±11.843 | 59.42±16.929 | 55.21±18.453 | 69.79±15.263 | 50.42±11.350 | 33.84±24.047 |
| Journal and online | 72.22±15.239 | 51.34±17.141 | 48.78±21.176 | 72.91±15.576 | 34.62±18.820 | 48.32±27.943 |
| *P*-value | 0.021 | 0.077 | 0.265 | 0.467 | 0.000 | 0.083 |
| **Level of Evidence Classification** | | | | | | |
| GRADE system | 79.24±13.380 | 59.81±14.378 | 58.60±18.093 | 78.47±13.740 | 40.17±19.182 | 56.75±23.541 |
| Oxford system | 78.60±15.813 | 59.80±15.274 | 62.90±19.319 | 73.10±16.319 | 37.10±24.223 | 37.90±33.017 |
| Other system | 68.27±14.53 | 47.64±17.4 | 47.4±18.452 | 69.43±14.884 | 34.75±17.51 | 45.32±29.719 |
| None | 67.19±14.807 | 42.13±15.224 | 31.21±17.920 | 66.30±16.240 | 29.28±17.241 | 32.89±24.900 |
| *P*-value | 0.000 | 0.000 | 0.001 | 0.001 | 0.238 | 0.025 |
| **No. of Authors** | | | | | | |
| >20 | 79.20±15.525 | 60.23±14.935 | 58.25±21.133 | 78.80±13.346 | 41.20±21.855 | 63.03±25.580 |
| 11—20 | 77.73±14.327 | 52.16±15.443 | 51.70±20.919 | 74.57±14.984 | 40.46±18.594 | 42.14±27.430 |
| 6—10 | 71.88±13.249 | 51.20±14.876 | 48.33±17.448 | 74.63±13.977 | 34.14±19.288 | 45.35±27.803 |
| ≤5 | 69.29±15.230 | 49.07±18.945 | 45.33±21.726 | 68.42±16.342 | 33.06±16.738 | 43.43±27.083 |
| *P*-value | 0.001 | 0.007 | 0.020 | 0.026 | 0.076 | 0.002 |
| **Method for guideline development** | | | | | | |
| Systematic review | 75.92±13.773 | 54.86±16.542 | 54.77±18.319 | 74.47±15.160 | 38.28±18.486 | 52.08±26.626 |
| Consensus or narrative review | 60.64±7.606 | 40.45±6.743 | 30.18±16.308 | 60.45±10.976 | 29.36±10.327 | 46.55±22.625 |
| Not mentioned | 63.37±17.390 | 42.63±17.927 | 30.39±19.310 | 67.68±16.248 | 27.32±19.684 | 25.32±23.903 |
| Adapted or adopted | 58.00 | 33.00 | 14.00 | 72.00 | 23.00 | 0.00 |
| *P*-value | 0.000 | 0.000 | 0.000 | 0.003 | 0.005 | 0.000 |
| **Recommendation methods** | | | | | | |
| Informal consensus | 68.38±11.830 | 48.06±15.088 | 49.91±16.211 | 67.73±15.112 | 33.96±14.723 | 50.07±24.698 |
| Formal consensus | 78.88±15.065 | 58.62±16.367 | 58.99±19.019 | 77.56±15.539 | 38.19±21.208 | 57.37±24.517 |
| Not mentioned | 71.01±15.902 | 48.85±17.771 | 40.36±21.889 | 71.72±14.862 | 35.11±19.020 | 36.00±28.897 |
| *P*-value | 0.000 | 0.000 | 0.000 | 0.002 | 0.505 | 0.000 |
| **No. of recommendations** | | | | | | |
| 1-10 | 72.98±16.905 | 50.77±19.310 | 47.83±23.275 | 71.64±17.695 | 34.65±20.016 | 46.30±28.889 |
| 11-20 | 74.08±14.280 | 53.22±17.185 | 49.05±21.304 | 73.93±14.042 | 36.18±19.494 | 46.74±27.562 |
| 21-30 | 73.10±13.571 | 52.03±14.625 | 54.76±15.445 | 72.97±15.244 | 34.93±15.955 | 47.62±26.014 |
| 31-40 | 76.08±14.582 | 59.00±12.307 | 54.92±16.429 | 72.33±16.708 | 42.33±16.104 | 57.75±26.626 |
| ≥41 | 68.04±13.275 | 49.38±13.928 | 46.25±18.772 | 72.17±11.702 | 38.00±16.983 | 45.25±29.012 |
| *P*-value | 0.749 | 0.477 | 0.517 | 0.586 | 0.818 | 0.800 |
| **Quality tool referral** | | | | | | |
| AGREE II | 79.25±10.779 | 61.83±14.302 | 60.45±16.742 | 76.88±15.391 | 45.22±18.954 | 59.95±28.667 |
| RIGHT | 73.20±16.932 | 58.20±20.969 | 55.80±24.652 | 72.00±17.986 | 46.60±25.265 | 65.00±12.629 |
| Combination | 82.80±10.337 | 67.80±19.412 | 69.10±14.738 | 88.70±7.103 | 41.10±13.127 | 71.60±10.658 |
| Not reported | 70.97±15.712 | 48.64±16.311 | 45.40±20.609 | 70.75±15.234 | 33.17±18.140 | 42.17±26.884 |
| *P*-value | 0.001 | 0.000 | 0.000 | 0.001 | 0.005 | 0.000 |
| **Defined time to update** | | | | | | |
| No | 70.28±15.766 | 47.97±16.649 | 43.79±19.725 | 70.64±14.562 | 32.06±18.110 | 41.84±25.975 |
| Yes | 78.19±12.533 | 59.76±15.696 | 59.90±19.339 | 76.49±16.690 | 43.36±17.985 | 57.17±28.785 |
| *P*-value | 0.000 | 0.000 | 0.000 | 0.006 | 0.000 | 0.000 |
| **External review** | | | | | | |
| No | 68.37±15.495 | 45.58±14.411 | 39.55±17.722 | 69.02±15.022 | 30.21±16.946 | 38.38±25.924 |
| Yes | 78.27±12.998 | 59.37±17.306 | 60.48±18.828 | 76.79±15.147 | 42.48±18.777 | 57.07±26.781 |
| *P*-value | 0.000 | 0.000 | 0.000 | 0.000 | 0.000 | 0.000 |
| **Version** | | | | | | |
| New | 72.38±15.333 | 51.35±17.560 | 48.57±21.644 | 72.19±15.556 | 34.97±19.312 | 46.25±28.174 |
| Old | 78.77±12.566 | 58.27±12.453 | 56.32±11.773 | 76.95±15.063 | 45.05±9.464 | 55.05±24.12 |
| *P*-value | 0.041 | 0.075 | 0.061 | 0.185 | 0.005 | 0.147 |
| **Publication of AGREE** | | | | | | |
| After | 73.93±15.056 | 53.02±17.202 | 49.85±21.384 | 74.15±14.688 | 36.02±19.234 | 46.85±28.568 |
| Before | 60.69±11.182 | 38.75±11.375 | 42.31±13.661 | 52.87±13.038 | 34.94±12.31 | 50.5±16.533 |
| *P*-value | 0.000 | 0.001 | 0.094 | 0.000 | 0.881 | 0.600 |
| **Publication of AGREE Ⅱ** | | | | | | |
| After | 74.37±15.658 | 54.55±17.219 | 51.14±21.897 | 75.56±14.615 | 36.35±20.009 | 48.19±29.249 |
| Before | 66.38±10.495 | 39.79±11.043 | 40.56±12.977 | 58.62±11.889 | 33.97±11.389 | 41.87±19.476 |
| *P*-value | 0.001 | 0.000 | 0.000 | 0.000 | 0.536 | 0.169 |
| **High quality (Domain 1, 2, 3, 4, 6 ≥ 60%)** | | | | | | |
| No | 70.65±14.715 | 48.57±15.768 | 44.68±18.655 | 70.81±15.373 | 33.31±17.044 | 41.81±26.237 |
| Yes | 87.28±8.942 | 73.03±8.855 | 77.59±8.776 | 83.84±11.459 | 52.00±21.269 | 79.38±10.392 |
| *P*-value | 0.000 | 0.000 | 0.000 | 0.000 | 0.000 | 0.000 |

**Table S12. Characteristics Associated with High-Quality Clinical Practice Guidelines.**

**Panel A. Primary standard maximum-likelihood logistic regression**

| **Characteristics** | | **Multivariable Logistic Regression** | |
| --- | --- | --- | --- |
|  |  | **Odds Ratio (95% CI)** | ***P* value** |
| **Recommendation methods** | **Informal consensus** | **1.17 [0.36-3.82]** | **0.795** |
|  | **Formal consensus** | **1.78 [0.65-4.87]** | **0.264** |
|  | **Not mentioned** | **1 [Reference]** |  |
| **External review** | **Yes** | **20.39 [4.66-89.27]** | **<0.001** |
|  | **No** | **1 [Reference]** |  |

**Panel B. Firth-penalized logistic regression**

| **Characteristics** | | **Firth-Penalized Logistic Regression** | |
| --- | --- | --- | --- |
|  |  | **Odds Ratio (95% CI)** | ***P* value** |
| **Recommendation methods** | **Informal consensus** | **1.17 [0.37-3.74]** | **0.716** |
|  | **Formal consensus** | **1.72 [0.66-4.81]** | **0.273** |
|  | **Not mentioned** | **1 [Reference]** |  |
| **External review** | **Yes** | **16.24 [5.08-82.10]** | **<0.001** |
|  | **No** | **1 [Reference]** |  |

**Table S13. 19-domain likelihood-ratio screening and diagnostic results**

| **Characteristics** | **Multivariable Logistic Regression** | | |
| --- | --- | --- | --- |
|  | **Likelihood-ratio** | **P value** | |
| **Year of publication** | **18.28** | **0.000** | |
| **Region** | **21.62** | **0.000** | |
| **Type of institution** | **10.66** | **0.031** | |
| **Funding** | **17.22** | **0.000** | |
| **Disease topic** | **4.14** | **0.529** | |
| **Type of guidelines** | **1.54** | **0.215** | |
| **Publication platform** | **0.23** | **0.628** | |
| **No. of authors** | **6.22** | **0.101** | |
| **No. of panel members** | **14.91** | **0.005** | |
| **Evidence grading system** | **19.25** | | **0.000** |
| **Method for guideline development** | **17.35** | **0.000** | |
| **Recommendation methods** | **8.12** | **0.017** | |
| **No. of recommendations** | **2.13** | **0.712** | |
| **Quality tool referral** | **13.19** | **0.004** | |
| **Defined time to update** | **18.51** | | **0.000** |
| **Publication of AGREE Ⅱ** | **0.03** | **0.864** | |
| **External review** | **37.97** | **0.000** | |
| **Version** | **1.32** | **0.250** | |
| **Language** | **5.19** | **0.023** | |

**Table S14. Sensitivity logistic regression analysis using lower 50% cutoffs.**

| **Characteristics** | | **Multivariable Logistic Regression** | |
| --- | --- | --- | --- |
|  |  | **Odds Ratio (95% CI)** | ***P* value** |
| **Recommendation methods** | **Informal consensus** | **1.38 [0.58-3.27]** | **0.471** |
|  | **Formal consensus** | **2.99 [1.38-6.46]** | **0.005** |
|  | **Not mentioned** | **1 [Reference]** |  |
| **External review** | **Yes** | **6.40 [3.20-12.79]** | **0.000** |
|  | **No** | **1 [Reference]** |  |

**Figure S1. Funnel Plots of Meta-analysis on Reporting Quality. (a) Comparisons between Chinese-language and English-language CPGs. (b) Comparisons between CPGs published before and after the RIGHT checklist release.**
