# Supplementary material for: Improvement of Clinical Practice Guideline Appraisal by Human Experts and AI Agents by Using Structured Guidance: Systematic Review, Meta-Analysis, and Validation Study
Source: J Med Internet Res. 2026 Aug 26;28:e96002. doi: 10.2196/96002 (PMC13559151; doi:10.2196/96002)
Supplement: Supplementary file 6 [file jmir_v28i1e96002_app6.docx]

**Multimedia Appendix 6**

**Large Language Model Evaluation**

[**AI Pipeline Technical Specifications**	3](#_Toc233740745)

[**General Technical Architecture**	3](#_Toc233740746)

[**Table S1. Shared Infrastructure Components**	3](#_Toc233740747)

[**Table S2. Model and Evaluation Conditions by Instrument**	5](#_Toc233740748)

[**Table S2A. Retrieval and LLM Inference Parameters**	5](#_Toc233740749)

[**RIGHT Checklist Pipeline**	7](#_Toc233740750)

[**Table S3. RIGHT Pipeline: Document Processing Parameters**	7](#_Toc233740751)

[**Table S4. RIGHT Pipeline: Scoring Item Dictionary**	10](#_Toc233740752)

[**eBox 1. RIGHT Checklist System Prompt**	12](#_Toc233740753)

[**eBox 2. RIGHT Checklist User Prompt Template**	13](#_Toc233740754)

[**AGREE II Pipeline**	14](#_Toc233740755)

[**Table S5. AGREE II Pipeline: Document Processing Parameters**	14](#_Toc233740756)

[**Table S6. AGREE II Pipeline: Scoring Item Dictionary**	17](#_Toc233740757)

[**eBox 3. AGREE II System Prompt (With Workbook)**	19](#_Toc233740758)

[**eBox 4. AGREE II System Prompt (Without Workbook)**	20](#_Toc233740759)

[**eBox 5. AGREE II User Prompt Template**	20](#_Toc233740760)

[**Large Language Model Evaluation Results**	22](#_Toc233740761)

[**Workbook Effects on Human–Agent Agreement**	22](#_Toc233740762)

[**Table S7. CPGs excluded from the LLM investigation (n=6) with reasons for exclusion.**	22](#_Toc233740763)

[**Table S8. Comparison of Agent-Human Scoring Agreement Under Two Knowledge Conditions.**	24](#_Toc233740764)

[**Enhanced Explainability with Workbook-Guided Justifications**	25](#_Toc233740765)

[**Table S9. Likert scores for textual justifications.**	25](#_Toc233740766)

[**Performance Plateau in RIGHT Checklist Evaluation**	26](#_Toc233740767)

[**Table S10. Intraclass Correlation Coefficients (ICC) for RIGHT Checklist Assessment.**	26](#_Toc233740768)

[**Cross-domain Generalizability and Efficiency Assessment**	28](#_Toc233740769)

[**Table S11. Comparison of Agent-Human Scoring Agreement in the Validation beyond Rehabilitation.**	28](#_Toc233740770)

[**Table S12. Comparison of External scoring - Human and External scoring - Agent Scoring Agreement.**	29](#_Toc233740771)

[**Table S13. Comparison of Agent-Human Appraisal Time.**	30](#_Toc233740772)

**AI Pipeline Technical Specifications**

**General Technical Architecture**

Both pipelines follow a retrieval-augmented generation (RAG) architecture implemented in Python using the LangChain framework. The system processes clinical practice guideline (CPG) documents in PDF format, retrieves relevant passages using semantic search, and generates structured appraisal scores through large language model (LLM) inference.

**Table S1. Shared Infrastructure Components**

| **Component** | **Specification** | **Details** |
| --- | --- | --- |
| Embedding Model | text-embedding-ada-002 (OpenAI) | 1536-dimensional dense vectors; industry standard for medical document retrieval |
| Orchestration Framework | LangChain (Python) | Provides modular components for document loading, text splitting, embedding, retrieval, and LLM interaction |
| Vector Store | FAISS (Facebook AI Similarity Search) | In-memory vector database used for fixed top-k nearest-neighbor retrieval |
| PDF Loader | PyPDFLoader (LangChain) | Extracts text with page-level metadata from PDF guideline documents |
| Document Loader (Criteria) | python-docx / Docx2txtLoader | Loads scoring criteria documents in DOCX format |

**Table S2. Model and Evaluation Conditions by Instrument**

| **Instrument** | **LLM model identifiers** | **Evaluation condition(s)** |
| --- | --- | --- |
| RIGHT Checklist | deepseek-r1 (DeepSeek-R1; April 2025 stable provider version); o1-mini (OpenAI o1-mini) | RIGHT Checklist reference-standard scoring; binary item-level classification. |
| AGREE II | deepseek-r1 (DeepSeek-R1; April 2025 stable provider version); o1-mini (OpenAI o1-mini) | AGREE II-only and AGREE II + Workbook conditions; EXP1 and EXP2 were independent full re-runs. |

**Table S2A. Retrieval and LLM Inference Parameters**

| **Parameter** | **RIGHT Checklist pipeline** | **AGREE II pipeline** |
| --- | --- | --- |
| API interface | OpenAI-compatible ChatOpenAI interface | OpenAI-compatible ChatOpenAI interface |
| LLM model identifier/version | deepseek-r1 (DeepSeek-R1; April 2025 stable provider version); o1-mini (OpenAI o1-mini) | deepseek-r1 (DeepSeek-R1; April 2025 stable provider version); o1-mini (OpenAI o1-mini) |
| Embedding model | text-embedding-ada-002 | text-embedding-ada-002 |
| Vector store | FAISS | FAISS |
| Similarity threshold | No minimum similarity-score threshold; fixed top-k retrieval only | No minimum similarity-score threshold; fixed top-k retrieval only |
| PDF chunking | 800-character chunks; 100-character overlap | 500-character chunks; 50-character overlap |
| Criteria processing | RIGHT Checklist DOCX split into 300-character chunks with 50-character overlap | AGREE II and Workbook DOCX documents loaded at paragraph level |
| PDF retrieval | Top-k = 4 | Top-k = 5 |
| Criteria retrieval | Top-k = 2 | Top-k = 5 |
| Decoding temperature | Provider default | Provider default |
| Maximum output tokens | Provider default | Provider default |
| Seed/determinism setting | Provider default; no seed specified | Provider default; no seed specified |
| Number of inference runs per item | One LLM call per item within each script execution | One LLM call per item within each script execution; EXP1 and EXP2 were independent full re-runs |

**RIGHT Checklist Pipeline**

The RIGHT Checklist pipeline evaluates CPGs against 35 sub-items across 7 domains, producing binary scores (0 = criterion not met; 1 = criterion met) with confidence estimates.

**Table S3. RIGHT Pipeline: Document Processing Parameters**

| **Parameter** | **Value** | **Rationale** |
| --- | --- | --- |
| PDF Chunk Size | 800 characters | Balances context preservation with retrieval precision for guideline documents |
| PDF Chunk Overlap | 100 characters | Maintains semantic continuity across chunk boundaries |
| Criteria Chunk Size | 300 characters | Smaller segments for precise retrieval of scoring criteria definitions |
| Criteria Chunk Overlap | 50 characters | Minimal overlap for criteria documents with self-contained paragraphs |
| Text Splitter | RecursiveCharacterTextSplitter | Hierarchically splits at paragraph, sentence, then character boundaries to minimize mid-sentence truncation |
| PDF Top-k Retrieval | k = 4 | Retrieves 4 most relevant passages from the guideline document |
| Criteria Top-k Retrieval | k = 2 | Retrieves 2 most relevant passages from the criteria document |
| Scoring Scale | Binary (0 or 1) | 0 = criterion absent; 1 = criterion present |
| Confidence Output | 0-100% | Model-reported confidence for each scoring decision |

**Table S4. RIGHT Pipeline: Scoring Item Dictionary**

The following 35 items are defined as a Python dictionary (right_items) and scored sequentially for each guideline:

| **Item** | **Description (Chinese / English Translation)** |
| --- | --- |
| 1a | Report whether the title identifies the document as a guideline. / 能够通过题目判断为指南。 |
| 1b | Report the year of publication. / 报告指南的发表年份。 |
| 1c | Report the type of guideline (e.g., screening, diagnosis, treatment, management, prevention). / 报告指南的分类。 |
| 2a | Provide a summary of recommendations (e.g., executive summary). / 对指南推荐意见进行汇总呈现。 |
| 3a | Define new or important terms and list abbreviations with full names. / 对新术语或重要术语进行定义。 |
| 4a | Provide contact information for at least one corresponding author. / 确定至少一位通讯作者的联系方式。 |
| 5a | Describe the basic epidemiology (prevalence, incidence, mortality, disease burden). / 描述问题的基本流行病学。 |
| 6a | Describe the overall objectives and specific aims of the guideline. / 描述指南的总目标和具体要达到的目的。 |
| 7a | Describe the primary target population. / 描述指南拟实施的主要目标人群。 |
| 7b | Describe subgroups requiring special consideration. / 描述需特别考虑的亚组人群。 |
| 8a | Describe the primary users (primary care providers, specialists, etc.). / 描述指南的主要使用者。 |
| 8b | Describe the specific settings (primary care, LMICs, inpatient). / 描述指南针对的具体环境。 |
| 9a | Describe all contributors and their roles (steering group, panel, external reviewers). / 描述参与指南制订的所有贡献者及其作用。 |
| 9b | Describe all individuals with titles, positions, and affiliations. / 报告所有参与者的头衔、职务、工作单位。 |
| 10a | Describe key questions underlying recommendations in PICO format. / 描述指南推荐意见所基于的关键问题（PICO格式）。 |
| 10b | Describe the method for selecting and classifying outcomes. / 描述结局遴选和分类的方法。 |
| 11a | Describe whether systematic reviews were newly conducted or existing. / 描述系统评价是新制作的还是使用现有已发表的。 |
| 11b | If using existing reviews, provide references and describe search/assessment; if new, describe search strategy. / 描述检索和评价过程。 |
| 12a | Describe the method for assessing and grading evidence quality. / 描述对证据质量评价和分级的方法。 |
| 13a | Provide clear, accurate, and implementable recommendations. / 提供清晰、准确且可实施的推荐意见。 |
| 13b | Provide separate recommendations for subgroups with significant differences. / 为重要亚组人群单独提供推荐意见。 |
| 13c | Describe recommendation strength and supporting evidence quality. / 描述推荐意见的强度及证据质量。 |
| 14a | Describe whether patient preferences and values were considered. / 是否考虑了目标人群的偏好和价值观。 |
| 14b | Describe whether costs and resource use were considered. / 是否考虑了成本和资源利用。 |
| 14c | Describe whether equity, feasibility, and acceptability were considered. / 是否考虑了公平性、可行性和可接受性。 |
| 15a | Describe the decision-making process and methods (consensus, voting). / 描述决策过程和方法。 |
| 16a | Describe whether independent external review was conducted. / 描述是否进行了独立评审。 |
| 17a | Describe quality control procedures. / 描述是否经过了质量控制程序。 |
| 18a | Describe funding sources at all stages. / 描述资金来源情况。 |
| 18b | Describe the role of funders at each stage. / 描述资助者在不同阶段中的作用。 |
| 19a | Describe types of conflicts of interest (financial and non-financial). / 描述利益冲突的类型。 |
| 19b | Describe COI evaluation/management methods and accessibility. / 描述利益冲突的评价和管理方法。 |
| 20a | Describe where the guideline and related documents can be accessed. / 描述指南的可及性。 |
| 21a | Describe gaps between current practice and evidence, or future research recommendations. / 描述实践与证据之间的差异及未来研究建议。 |
| 22a | Describe all limitations in the guideline development process and their impact. / 描述指南制订过程中的所有局限性。 |

**eBox 1. RIGHT Checklist System Prompt**

\# Role You are a medical guideline scoring expert proficient in the RIGHT Checklist framework. You independently evaluate 35 items across medical guidelines, ensuring each score is based on scoring criteria, standards, and reasonable rationale. Your scoring process must be completely fair, transparent, and free from external influence. # Skills ### Skill 1: Document Knowledge Parsing Extract the 35 scoring dimensions across 7 domains and 22 items of the RIGHT Checklist. ### Skill 2: Structured Scoring Workflow **Step 1: Evidence Extraction** Perform paragraph-level semantic analysis on the input medical guideline, automatically annotating potentially relevant evidence segments. **Step 2: Item Matching** For each scoring item, perform a presence judgment: determine whether the guideline contains specific content meeting the requirements of that RIGHT Checklist item. **Step 3: Result Output (Critical)** Output confidence-weighted scoring results based on the presence of each item. - **1 point**: The guideline **contains** the information required by this item. - **0 points**: The guideline **does not contain** the information required by this item. ### Skill 3: Validation Mechanism 1. **Consistency Check**: Verify that individual item scoring meets RIGHT Checklist requirements. 2. **Overall Validation**: Ensure scoring objectively reflects the guideline's reporting quality. # Constraints - The scoring process must be independent, transparent, and free from external interference. - After each scoring, you must "forget" the specific guideline content to maintain independence. - Information retrieval is limited to content directly relevant to the current scoring task. - **Output requirement**: No reasoning or evidence location needed; **output score and confidence only**. - All output must be in Chinese.

**eBox 2. RIGHT Checklist User Prompt Template**

Please score RIGHT Checklist item **{item_code}**. **Item Description**: {item_description} **RIGHT Checklist Reference Standard**: {criteria_context} **Guideline Excerpts**: {pdf_context} **Task Instructions**: Please apply your expert skills to analyze whether the guideline excerpts above meet the requirements of the item description. Although you need to perform rigorous evidence extraction and matching, please **output only** the final scoring result. **Output Format (strict compliance required)**: Score: [0 or 1] | Confidence: [0-100]%

**AGREE II Pipeline**

The AGREE II pipeline evaluates CPGs against 23 items across 6 domains, producing scores on a 7-point Likert scale (1 = strongly disagree to 7 = strongly agree) with detailed reasoning. The pipeline operates under two conditions: (1) with the Workbook (dual-document scoring), and (2) without the Workbook (AGREE II framework alone).

**Table S5. AGREE II Pipeline: Document Processing Parameters**

| **Parameter** | **Value** | **Rationale** |
| --- | --- | --- |
| PDF Chunk Size | 500 characters | Smaller chunks enable more granular evidence retrieval for nuanced 7-point scoring |
| PDF Chunk Overlap | 50 characters | Minimal overlap; fine-grained chunks preserve precise evidence boundaries |
| Criteria Processing | Paragraph-level (python-docx) | AGREE II criteria and Workbook paragraphs loaded individually without further splitting |
| Text Splitter (PDF) | RecursiveCharacterTextSplitter | Hierarchical splitting at natural language boundaries |
| PDF Top-k Retrieval | k = 5 | Higher retrieval count to support comprehensive evidence review for qualitative assessment |
| Criteria Top-k Retrieval | k = 5 | Retrieves from combined AGREE II + Workbook vector store |
| Criteria Sources | AGREEII.docx + Workbook.docx | Two documents concatenated and embedded into a single vector store |
| Scoring Scale | 1-7 Likert scale | 1 = strongly disagree; 7 = strongly agree; 4 = neutral |
| Conflict Resolution | Workbook takes precedence | When AGREE II and Workbook interpretations diverge, Workbook guidance is prioritized |

**Table S6. AGREE II Pipeline: Scoring Item Dictionary**

| **Item** | **Description (Chinese / English Translation)** |
| --- | --- |
| 1 | The overall objective(s) of the guideline is(are) specifically described. / 指南的总体目标是否具体描述。 |
| 2 | The health question(s) covered by the guideline is(are) specifically described. / 指南涵盖的健康问题是否具体描述。 |
| 3 | The population (patients, public, etc.) to whom the guideline is meant to apply is specifically described. / 指南适用的目标人群是否具体描述。 |
| 4 | The guideline development group includes individuals from all relevant professional groups. / 指南开发小组是否包括所有相关专业群体。 |
| 5 | The views and preferences of the target population (patients, public, etc.) have been sought. / 是否征求了目标人群的意见和偏好。 |
| 6 | The target users of the guideline are clearly defined. / 指南的目标用户是否明确定义。 |
| 7 | Systematic methods were used to search for evidence. / 是否使用系统方法搜索证据。 |
| 8 | The criteria for selecting the evidence are clearly described. / 证据选择标准是否清楚描述。 |
| 9 | The strengths and limitations of the body of evidence are clearly described. / 证据的强弱和局限性是否清楚描述。 |
| 10 | The methods for formulating the recommendations are clearly described. / 制定推荐的方法是否清楚描述。 |
| 11 | The health benefits, side effects, and risks have been considered in formulating the recommendations. / 是否考虑了健康益处、副作用和风险。 |
| 12 | There is an explicit link between the recommendations and the supporting evidence. / 推荐与支持证据之间是否有明确联系。 |
| 13 | The guideline has been externally reviewed before its publication. / 指南在发布前是否由专家外部审查。 |
| 14 | A procedure for updating the guideline is provided. / 是否提供了更新指南的程序。 |
| 15 | The recommendations are specific and unambiguous. / 推荐是否具体且明确。 |
| 16 | The different options for management of the condition or health issue are clearly presented. / 不同管理选项是否清楚呈现。 |
| 17 | Key recommendations are easily identifiable. / 关键推荐是否易于识别。 |
| 18 | The guideline describes facilitators and barriers to its application. / 是否描述了应用的促进因素和障碍。 |
| 19 | The guideline provides advice and/or tools on how the recommendations can be put into practice. / 是否提供将推荐付诸实践的建议或工具。 |
| 20 | The potential resource implications of applying the recommendations have been considered. / 是否考虑了应用推荐的潜在资源影响。 |
| 21 | The guideline presents monitoring and/or auditing criteria. / 是否呈现监测和/或审计标准。 |
| 22 | The views of the funding body have not influenced the content of the guideline. / 资助方的观点是否未影响指南内容。 |
| 23 | Competing interests of guideline development group members have been recorded and addressed. / 小组成员的竞争利益是否记录并处理。 |

**eBox 3. AGREE II System Prompt (With Workbook)**

\# Role You are a dual-standard medical guideline scoring expert proficient in both the AGREE II framework and the Workbook operational guidelines. You possess dual-knowledge fusion capability. You independently evaluate 23 scoring items of medical guidelines, ensuring each score is based on scoring criteria, standards, and reasonable rationale. Your scoring process must be completely fair, transparent, and free from external influence. During scoring, you automatically align the scoring logic of both documents, prioritizing the Workbook's specific case analyses as the scoring basis, and cross-validating with both documents for ambiguous areas. ## Skills ### Skill 1: Dual-Document Knowledge Fusion 1. Layered parsing mechanism: - Layer 1: Extract AGREE II's 6 domains, 23 core scoring dimensions, and their weight framework. - Layer 2: Convert the Workbook content into an executable scoring rule library: - Positive/negative evidence examples for each item (e.g., "three scenarios for Item 3 scoring 4 points")	- Ruling standards for common controversial scenarios (e.g., "weighted calculation for partial fulfillment")	- Applicability instructions for special clauses (e.g., "not applicable (NA) determination threshold") 2. Knowledge graph construction: - Establish an association matrix between both documents, marking: - Direct correspondence (e.g., Workbook Chapter X corresponds to AGREE II Item Y)	- Supplementary relationships (e.g., Workbook provides 5 scenario explanations for AGREE II Item Z's ambiguous clause)	- Conflict handling principles (when the two documents conflict, prioritize the latest Workbook version) ### Skill 2: Independent Scoring 1. Score 23 AGREE II items independently according to learned scoring standards. 2. For each item: first clearly state scoring criteria, then give a specific score, and provide detailed, reasonable, and convincing rationale. ### Skill 3: Structured Scoring Workflow 1. Step 1: Evidence extraction (paragraph-level semantic analysis with automatic evidence annotation). 2. Step 2: Item matching (three-level judgment: basic compliance, detailed compliance, exception handling). 3. Step 3: Dynamic scoring using the Workbook's evidence-weight calculation method. ### Skill 4: Dual Verification Mechanism 1. Consistency checks (horizontal and vertical validation). 2. Traceability verification (dual-document source marking required for each scoring conclusion). ## Constraints - When Workbook details conflict with AGREE II base clauses, the latest Workbook interpretation prevails. - Scoring must be independent, transparent, and free from external interference. - After each scoring, "forget" the guideline content to maintain independence. - Each item must simultaneously cite both documents: "Scoring basis: AGREE-II Sec.X + Workbook Sec.Y". - Information retrieval limited to directly relevant content. - For uncovered special cases, use the Workbook's "analogy reasoning rules" within the AGREE II framework. - Generate dual-perspective analysis reports for controversial items.

**eBox 4. AGREE II System Prompt (Without Workbook)**

\# Role You are a medical guideline scoring expert proficient in the AGREE II framework. You independently evaluate 23 scoring items of medical guidelines, ensuring each score is based on scoring criteria, standards, and reasonable rationale. Your scoring process must be completely fair, transparent, and free from external influence. ## Skills ### Skill 1: Document Knowledge Parsing - Extract the AGREE II 6 domains, 23 core scoring dimensions, and their weight framework. ### Skill 2: Independent Scoring 1. Score 23 AGREE II items independently according to AGREE II standards (1-7 points). 2. For each scoring item, provide: - Specific score	- Scoring confidence (percentage)	- Detailed, reasonable, and convincing rationale	- Key evidence extracted from the guideline ### Skill 3: Structured Scoring Workflow 1. Step 1: Evidence extraction (paragraph-level semantic analysis with automatic evidence annotation). 2. Step 2: Item matching (two-level judgment): - Basic compliance: whether AGREE II minimum requirements are met	- Detailed compliance: whether AGREE II additional requirements for this item are met 3. Step 3: Dynamic scoring using evidence-weight calculation: - Base score (AGREE II standard requirements) + adjustment score (AGREE II additional requirements for this item)	- Output confidence-weighted scoring results ### Skill 4: Verification Mechanism 1. Consistency checks: - Horizontal validation: compare current item score with related items for logical consistency	- Vertical validation: verify individual item scores satisfy AGREE II framework requirements 2. Traceability verification: - Each scoring conclusion must include document source marking: - AGREE II clause number (e.g., Domain 3.2) ## Constraints - Scoring must be independent, transparent, and free from external interference. - After each scoring, "forget" the guideline content to maintain independence. - Each item must cite AGREE II document basis: "Scoring basis: AGREE-II Sec.X". - Information retrieval limited to directly relevant content. - Final output must include: score, confidence, rationale, and evidence.

**eBox 5. AGREE II User Prompt Template**

Please score AGREE II item {item_number} based on the following information: Item Description: {item_description} Scoring Basis (extracted from AGREE II [and Workbook]): {criteria_context} Guideline Excerpts: {pdf_context} Based on the scoring criteria and guideline content, assign a score of 1-7 and provide detailed, reasonable rationale. [If AGREE II and Workbook conflict, Workbook takes precedence.] In the rationale, please cite specific sources (e.g., "according to AGREE II Section X" or "according to Workbook Section Y"). Output Format: Score: [1-7] Confidence: [0-100]% Reasoning: [detailed justification with cited sources] Evidence: [key excerpts from the guideline supporting this score]

**Large Language Model Evaluation Results**

**Workbook Effects on Human–Agent Agreement**

**Table S7. CPGs excluded from the LLM investigation (n=6) with reasons for exclusion.**

| **Title** | **Reason for Exclusion** |
| --- | --- |
| Ottawa Panel evidence-based clinical practice guidelines for strengthening exercises in the management of fibromyalgia | The guideline is divided into Part 1 and Part 2. The agent has limited capability to retain and integrate information across multiple sections, which makes it difficult to consistently read the guideline and may lead to confusion during interpretation. |
| Stroke rehabilitation in adults | Due to the excessive number of tokens in the guideline, the agent is unable to process the entire document in a single pass. |
| Women-Focused Cardiovascular Rehabilitation: An International Council of Cardiovascular Prevention and Rehabilitation Clinical Practice Guideline | When the guideline is provided in a file-plus-nearby-text format, the agent may experience context fragmentation, resulting in confusion and an inability to perform accurate scoring. |
| 中国脑性瘫痪康复指南(2015) | The guideline is divided into Part 1, Part 2, and Part 3. The agent’s limited memory capacity for cross-part integration makes comprehensive reading difficult and increases the risk of misinterpretation. |
| 中国脑性瘫痪康复指南(2022) | The guideline is divided into Part 1, Part 2, and Part 3. The agent’s restricted ability to merge information across multiple sections may lead to reading inconsistencies and confusion. |
| Cardiac Rehabilitation as Secondary Prevention Quick Reference Guide for Clinicians.pdf | The document is provided in a scanned format, which results in garbled or unrecognizable text during extraction, preventing reliable reading and analysis. |

**Table S8. Comparison of Agent-Human Scoring Agreement Under Two Knowledge Conditions.**

| Experimental Condition | Metric | Human Experts | DeepSeek-R1 | | O1-mini | |
| --- | --- | --- | --- | --- | --- | --- |
|  |  |  | EXP1 | EXP2 | EXP1 | EXP2 |
| AGREE II Only | Default ICC | 0.752 | 0.625 | 0.616 | 0.629 | 0.636 |
|  | Modified ICC | 0.761 | 0.617 | 0.608 | 0.628 | 0.629 |
|  | Cohen's Kappa | 0.366 | 0.248 | 0.146 | 0.126 | 0.138 |
| AGREE II + Workbook | Default ICC | 0.803 | 0.716 | 0.712 | 0.685 | 0.702 |
|  | Modified ICC | 0.807 | 0.713 | 0.705 | 0.681 | 0.692 |
|  | Cohen's Kappa | 0.413 | 0.303 | 0.322 | 0.248 | 0.247 |

Note. To avoid selectively reporting different human raters, summary text in the Abstract and Results uses the arithmetic mean of the modified ICCs across EXP1 and EXP2 for both models: DeepSeek-R1, 0.613 without the Workbook and 0.709 with the Workbook; O1-mini, 0.629 and 0.687, respectively.

**Enhanced Explainability with Workbook-Guided Justifications**

**Table S9. Likert scores for textual justifications.**

|  | | Likert Scores | | | | | |
| --- | --- | --- | --- | --- | --- | --- | --- |
|  |  | 1(%) | 2(%) | 3(%) | 4(%) | 5(%) | Mean±SD |
| O1-mini + Workbook | EXP1 | 0 | 0 | 7.0 | 33.7 | 59.3 | 4.52±0.63 |
|  | EXP2 | 0 | 0 | 8.5 | 23.6 | 67.8 | 4.59±0.64 |
| O1-mini | EXP1 | 0 | 0 | 10.6 | 20.6 | 68.8 | 4.58±0.68 |
|  | EXP2 | 0 | 0 | 10.1 | 22.6 | 67.3 | 4.57±0.67 |
| DeepSeek-R1 + Workbook | EXP1 | 0.5 | 6 | 9.5 | 34.2 | 49.7 | 4.27±0.90 |
|  | EXP2 | 0.5 | 5.5 | 12.1 | 34.7 | 47.2 | 4.23±0.90 |
| DeepSeek-R1 | EXP1 | 0 | 2.5 | 25.6 | 24.6 | 47.2 | 4.17±0.90 |
|  | EXP2 | 0 | 3.0 | 22.6 | 24.6 | 49.7 | 4.21±0.90 |

**Performance Plateau in RIGHT Checklist Evaluation**

**Table S10. Intraclass Correlation Coefficients (ICC) for RIGHT Checklist Assessment.**

| Domain / Group | Overall | D1 | D2 | D3 | D4 | D5 | D6 | D7 |
| --- | --- | --- | --- | --- | --- | --- | --- | --- |
| Human Consensus | 0.851 | 0.849 | 0.882 | 0.802 | 0.829 | 0.818 | 0.871 | 0.845 |
| O1-mini | 0.623 | 0.425 | 0.540 | 0.724 | 0.712 | 0.673 | 0.594 | 0.559 |
| DeepSeek-R1 | 0.621 | 0.467 | 0.540 | 0.697 | 0.712 | 0.681 | 0.624 | 0.529 |

**Cross-domain Generalizability and Efficiency Assessment**

**Table S11. Comparison of Agent-Human Scoring Agreement in the Validation beyond Rehabilitation.**

| Experimental Condition | Metric | Human Experts | DeepSeek-R1 | | O1-mini | |
| --- | --- | --- | --- | --- | --- | --- |
|  |  |  | EXP1 | EXP2 | EXP1 | EXP2 |
| AGREE II + Workbook | Default ICC | 0.755 | 0.887 | 0.785 | 0.881 | 0.746 |
|  | Modified ICC | 0.736 | 0.879 | 0.779 | 0.868 | 0.738 |
|  | Cohen's Kappa | 0.356 | 0.273 | 0.214 | 0.268 | 0.241 |

**Table S12. Comparison of External scoring - Human and External scoring - Agent Scoring Agreement.**

| Metric | EXP1 | EXP2 | DeepSeek-R1 | O1-mini |
| --- | --- | --- | --- | --- |
| Default ICC | 0.734 | 0.651 | 0.711 | 0.680 |

**Table S13. Comparison of Agent-Human Appraisal Time.**

|  | Overall Appraisal Time (min) | Appraisal Time per CPG (min) |
| --- | --- | --- |
| EXP1 | 89.80 | 14.97 |
| EXP2 | 44.32 | 7.39 |
| HUMAN AVERAGE | 67.06 | 11.18 |
| DeepSeek-R1 | 32.62 | 5.44 |
| O1-mini | 21.08 | 3.51 |
| AGENT AVERAGE | 26.85 | 4.48 |
